# Polyketide synthase-based controlled synthesis of polycyclopropanated fuel molecules

**DOI:** 10.1038/s41467-026-73172-3

**Published:** 2026-05-27

**Authors:** Kevin Yin, Alexander Landera, Namil Lee, Anthony T. Iavarone, Suzanne M. Kosina, Thomas D. Young, Kai Deng, Justin Baerwald, Yan Chen, Jennifer W. Gin, Riley Benedict, Yan Chiu, Ezechinyere Ukabiala, Methun Kamruzzaman, Kunal Poorey, Trent R. Northen, Christopher J. Petzold, Anthe George, Pablo Cruz-Morales, Qingyun Dan, Jay D. Keasling

**Affiliations:** 1 Joint Bioenergy Institute, Emeryville, CA USA; 2 Biological Systems and Engineering Division, Lawrence Berkeley National Laboratory, Berkeley, CA USA; 3 Department of Plant and Microbial Biology, University of California, Berkeley, CA USA; 4 Department of Chemical and Biomolecular Engineering, University of California, Berkeley, CA USA; 5 Biomass Science and Conversion Technology Department, Sandia National Laboratories, Livermore, CA USA; 6 QB3 Institute, University of California, Berkeley, CA USA; 7 Graduate School of Engineering Biology, Korea Advanced Institute of Science and Technology (KAIST), Daejeon, Republic of Korea; 8 Environmental Genomics and Systems Biology Division, Lawrence Berkeley National Laboratory, Berkeley, CA USA; 9 Department of Biomaterials and Biomanufacturing, Sandia National Laboratories, Livermore, CA USA; 10 Department of Chemistry, University of California, Berkeley, Berkeley, CA USA; 11 Department of Molecular and Cell Biology, University of California, Berkeley, CA USA; 12 Department of Bioengineering, University of California, Berkeley, CA USA; 13 Department of Bioengineering, University of California San Francisco, San Francisco, CA USA; 14 Department of Material Lifecycle Management, Sandia National Laboratories, Livermore, CA USA; 15 The Novo Nordisk Foundation Center for Biosustainability, Technical University of Denmark, Kemitorvet 220, Kongens, Lyngby, Denmark; 16 The Novo Nordisk Foundation Biotechnology Research Institute for the Green Transition, Technical University of Denmark, Kongens Lyngby, Denmark; 17 Department of Biotechnology and Biomedicine, Technical University of Denmark, Kongens Lyngby, Denmark

**Keywords:** Biocatalysis, Metabolic engineering, Applied microbiology

## Abstract

Reducing carbon emissions from aviation and long-distance transportation sectors requires the development of sustainable biofuels with suitable energy density, freezing point, and other physical properties. We previously demonstrated biological production of high energy polycyclopropanated fatty acids (POP-FAs, class I) using an iterative polyketide synthase (iPKS) pathway in a *Streptomyces* host. Here, we used a computational model of fuel properties to identify chain length and cyclopropanation control as critical steps to engineer this iPKS for biofuel applications. We next explored the natural diversity of POP biosynthesis by investigating homologous pathways. Then, by in vivo gene exchange, we determined cyclopropanase (CP) catalysis to be key for POP-FA engineering. Leveraging both natural and engineered pathway product diversity, we demonstrate targeted production of improved POP-FAs, namely shortened POP-FAs with predicted superior freezing point properties for aviation, as well as fully cyclopropane-saturated POP-FAs which should have superior energy-density. These precise and controllable modifications to POP-FA structure open the door for bioproduction of designer POP fuels.

## Abstract (web summary)

To be practically useful, sustainable aviation fuels need a suitable energy density and freezing point. Here the authors explore an iterative polyketide synthase family, leading to engineered biosynthesis of medium chain, fully cyclopropanated fatty acids as jet fuel precursors with ideal properties.

## Introduction

Decarbonization of all sectors is urgently needed to mitigate human-made climate change. In tandem with the development of low-emission technologies (solar, wind, and lithium-ion batteries), electric vehicles (EVs) offer significant decarbonization potential for short-range land-based transport^1^. However, long-range or heavy duty transport requires higher power-to-weight ratios: the energy density of lithium ion batteries is only ~1 MJ/kg^2^ whereas jet fuels must have a heat of combustion of at least 42.8 MJ/kg^3^. Furthermore, aviation emissions are projected to increase from 1.03 GtCO_2_ in 2019 to up to 1.9 GtCO_2_ by 2050, and the pathway to emission reduction relies heavily on replacing fossil fuels with biofuels^4,5^. Thus, the development of energy-dense biofuels remains important for mitigating CO_2_ emissions from aviation and other sectors that are challenging to electrify.

In addition to increased energy density (e.g., via strained bond angles), the next generation of biofuels will need customizable carbon skeleton design to meet the combustion energy, freezing point, and boiling point demands of aviation^6^. Of all hydrocarbons, the strained cyclopropane ring is among the most energy-dense moieties. Energy-dense cyclopropanes were first demonstrably leveraged for fuel purposes with syntin, which was used with oxygen in Soyuz and Proton rockets^7–10^. However, organic synthesis of syntin was toxic, costly, and derived from petroleum. To address these drawbacks, synthesis of a polycyclopropanated, high performance bio-kerosene^11^ has recently been demonstrated via the cyclopropanation of myrcene, which can be derived from tree sap^12^. Additionally, a recyclable and more efficient catalyst for Simmons-Smith cyclopropanation, HPW/MCM-41, has been recently demonstrated by the same group^13^. Compared to existing syntin synthesis, this new catalytic method is more sustainable; however, both routes still require a stoichiometric amount of diethylzinc. Biological conversion on the other hand can generate fuels from inexpensive, low-energy carbon sources such as lignocellulosic biomass, providing much greater opportunity for economically viable fuels that are carbon neutral or negative^14,15^. Our approach therefore was to search polycyclopropanated molecules in nature, design plausible polycyclopropanated structures for biofuel applications based on energy property computation, and engineer their biosynthetic pathways for target biofuel synthesis in a microorganism.

**Fig. 1:**
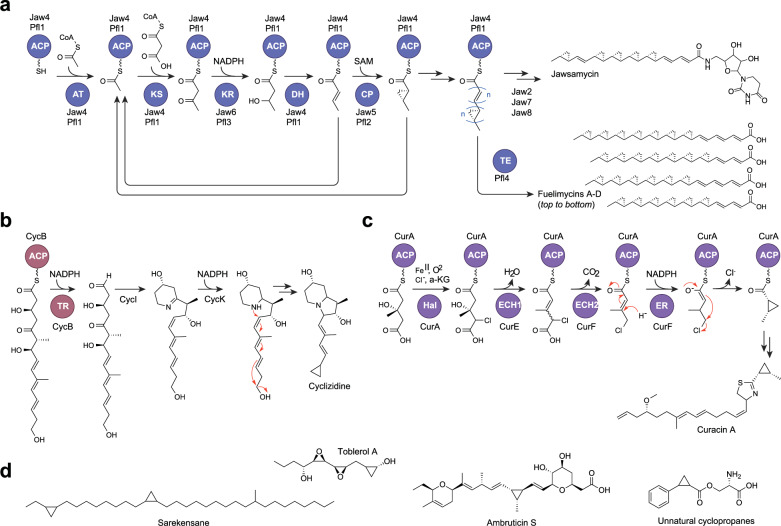
Cyclopropane containing natural products and their pathways. **a** The iterative PKS pathways (jawsamycin, Jaw; fuelimycin, Pfl) for polycyclopropanated (POP) polyketides. **b** The cyclizidine pathway. **c** The curacin pathway. ACP acyl carrier protein, AT acyl transferase KS ketosynthase, KR ketoreductase, DH dehydratase, CP cyclopropanase, TR terminal reductase, Hal halogenase, ECH1 dehydratase, ECH2 decarboxylase, ER enoyl reductase. **d** Sarekensane, toblerol A, ambruticin S, and unnatural cyclopropanes formed via carbene transfer. Despite the diversity of cyclopropanated structures, none of these molecules have suitable energy density for aviation biofuel applications.

A major way of synthesizing structurally complex natural products is through multidomain enzymatic complexes called polyketide synthases (PKSs). Owing to their large design space^16^, PKSs are a promising platform for production of designer biomolecules^17–19^ including biofuels^20–23^, and have the potential to meet the carbon chain length, functional groups, and structural requirements for the energy density, freezing point, and other desired properties of an aviation biofuel. In particular, we recently demonstrated production of polycyclopropanated fatty acids (POP-FAs) by leveraging an iterative PKS (iPKS) pathway which we named fuelimycin (Fig. 1a)^24^. The polycyclopropanating iPKS (POP-iPKS) contains a multidomain iPKS with ketosynthase (KS), acyltransferase (AT), dehydratase (DH), and acyl-carrier protein (ACP) domains complexed to three standalone domains: ketoreductase (KR), cyclopropanase (CP), and thioesterase (TE). While the POP-iPKS produced highly cyclopropanated fatty acids, their chain length and the number of cyclopropane rings were not ideal for jet fuels. To approach the properties desired in aviation fuels, control of POP-iPKS pathways must be engineered.

A key factor of POP-iPKS engineering lies in controlled synthesis of cyclopropanes, a canonical topic in organic chemistry. In fact, cyclopropane biosynthesis is widespread in nature and serves a variety of evolutionary purposes. Within the membrane lipids of a broad range of bacteria including *Escherichia coli*, *Salmonella*, and *Streptococcus*, cyclopropane rings improve tolerance to environmental stressors including high osmotic pressure, low pH, and high temperatures^25,26^. In the mycolic acids of *Mycobacterium tuberculosis*, cyclopropanes are notably associated with increased pathogenesis^27–29^. Additionally, several bioactive polyketides contain a single cyclopropane ring^30^, including cyclizidine^31,32^ (Fig. 1b), curacin^33^ (Fig. 1c), ambruticin S^34^, and toblerol A^35^. Bioproduction of unnatural cyclopropanes has also been demonstrated via carbene transfer^36^. Recently, a long chain hydrocarbon containing two cyclopropane rings, named sarekensane, has even been reported from the cuticle of *Vertagopus sarekensis*, a springtail (Collembola) arthropod (Fig. 1d)^37,38^. Finally, formation of more than two cyclopropane rings in a single biomolecule remains unique to POP-iPKS pathways including that of jawsamycin (FR-900848), U-106305, and fuelimycin^24,39–42^ (Fig. 1a). We named these POP-FAs using a “C[x]:CP[y]” nomenclature, whereby x represents the number of carbons in the “main chain” (i.e., excluding the carbons added by the cyclopropanase) and y represents the number of cyclopropane rings. Because POP-FA biosynthesis is iterative, the determining factors for the main-chain length as well as the number and position of cyclopropane rings in the final product(s) are not readily apparent. Without characterized product control mechanisms, design rules cannot be established for engineering POP-iPKS pathways. As such, POP-iPKSs must be further studied to match the engineering potential of assembly-line PKSs with known collinearity between domain architecture and product structure^43^. Fortuitously, differences in main-chain length and cyclopropanation exist among the fuelimycins (C22:CP7, C20:CP7, C20:CP6, C18:CP6), jawsamycin (C18:CP5), and U-106305 (C18:CP6). Our characterization of the fuelimycin biosynthetic gene cluster (BGC) and its products thus highlights an opportunity to expand genome mining efforts for further discovery of novel POP biochemistry. Subsequently, rational domain exchanges between homologous synthases^44–48^ may reveal the role of each domain in determining the end products and finally open the possibility for programmable biosynthesis of POP molecules.

Here we report diversified natural and chimeric POP-iPKS pathways and demonstrate controllable modifications to the POP-FA structure towards various fuel property targets. First, we identify sequence- and structure-level evidence that POP CP divergence leads to the diversification of POP product chain length and cyclopropane saturation. Then, we demonstrate production of shortened (C12:CP4, C14:CP5) POP-FAs. With shorter chain lengths than fuelimycins and distinct cyclopropane positions, we predict decarboxylated hydrocarbon derivatives of these molecules to be fuels with energy density improvements and comparable freezing points to cyclopropanated myrcene^11^. Next, we demonstrate production of cyclopropane-saturated POP-FAs (C14:CP6, C16:CP7, C18:CP8, C20:CP9, C22:CP10 and C24:CP11), which are among the most energy-dense molecules that can be produced by a reported biological system. Taken together, our results expand the diversity of POP structures that can be biosynthesized, and lay the groundwork for further rational engineering of POP-iPKS pathways to achieve designer biofuel synthesis and global aviation emissions reduction.

## Results

### Determining target POP molecules

Upon our first discovery of fuelimycins A-D (Fig. 1a), one critical feature that curtails these molecules from becoming acceptable jet-fuel chemicals is the existence of two or three alkenes in their structures. Being susceptible to oxidative or thermal breakdown^49,50^, alkenes generally do not meet the stability requirements of sustainable aviation fuels. Furthermore, each double bond represents a missed opportunity for energy-dense cyclopropane ring formation. Based on our previous computational model, the predicted energy density as volumetric net heat of combustion (NHOC) of fuelimycin A-derived fatty acyl methyl ester (FAME), which contains three double bonds (C22:CP7-FAME), is 40.6 MJ/L. In comparison, C12:CP5-FAME’s predicted volumetric NHOC is 45.14 MJ/L, despite having ten fewer main-chain carbons and two fewer cyclopropane rings^24^. Thus, increasing cyclopropane ratio, via replacement of unsaturated hydrocarbons with cyclopropane rings, is a major and necessary fuel upgrade that we sought to pursue biosynthetically.

Furthermore, as aircraft frequently encounter low operating temperatures, aviation fuels must strictly have freezing points below −47 °C^3^ to be suitable for intercontinental flights and avoid problems associated with crystallization^51^. While the freezing point of a structurally similar bio-kerosene (cyclopropanated myrcene) has been characterized^11^, the estimated titers of fuelimycins have been too low for direct measurement of freezing point and other POP-FA properties (Supplementary Table 1). Instead, we calculated the specific energy, energy density, and freezing point properties of POP molecules theoretically. As our previous model predicted POP-alkanes and -alkenes to have higher energy densities than POP-FAMEs^24^, we focused on POP-alkanes and -alkenes for our new calculations.

To predict the freezing points of fuel molecules, a machine learning model^52^ trained on a dataset of 19,811 data points^53^ using 2D PaDel descriptors^54^ demonstrated high accuracy with a mean absolute error (MAE) of 23.69 °C after outlier removal refinement. However, the model’s performance degraded for cyclopropane-containing oligomers, and particularly for fully cyclopropane-saturated POP molecules. Retraining the model with 37 additional reference freezing points significantly improved its accuracy for these challenging molecules, reducing errors in key examples such as 1,1’:2’,1”-tercyclohexane and syntin (Supplementary Table 2, Supplementary Data 1). As a result, we observed a positive correlation between the density and freezing point of the POP fuels whose physical properties were calculated. The highest freezing points were found to belong to cyclopropane oligomers without a missing cyclopropane unit. The lowest freezing points were found to occur when a cyclopropane unit is missing and is replaced by a single bond. However, when replaced by a double bond, the freezing point does still decrease considerably relative to the cyclopropane oligomers without a missing cyclopropane unit. The opposite trend occurs when looking at specific energy or energy density. The stereochemistry of a molecule can have profound effects on its melting point. Rotational isomers without bulky chemical groups are able to effectively access all rotational degrees of freedom. These molecules can have lower melting points because it can be harder to adopt a preferred melting point orientation until the temperature drops low enough that molecules are able to align and form crystals. Those with bulky groups can only sample a restricted range of rational angles and thus can more easily conform to a melting point orientation at higher temperature. A simple example of this can be seen from analyzing the trend in melting points for halo-ethanes. The melting points of fluoroethane, chloroethane, bromoethane, and iodoethane are −142.8 °C, −138.7 °C, −119 °C, and −111.1 °C, respectively. As the halo group becomes bulkier, the melting point increases because steric hindrance does not allow for free rotation of the CH_2_-X group, and the preferred orientation for melting is more easily obtained. Of course, there are many other factors that can affect melting point, like atomic charge and dipole moments, and this trend in melting point as a function of bulky chemical groups is only meant to illustrate one factor that affects the melting point of rotational isomers. Our melting point model is trained on a large dataset with a variety of rotational isomers. However, when making predictions it takes in a SMILES notation, which does not include rotational isomer data, and so only takes into account rotational isomerism implicitly.

**Fig. 2:**
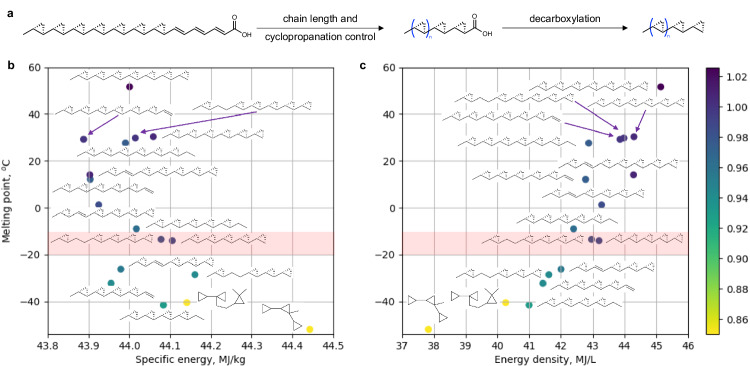
Predicted freezing point and energy properties of POP alkanes and other fuels. **a** Proposed route for POP alkane synthesis. While not the focus of this work, chemical and enzymatic decarboxylation methods may be explored in the future. **b** A plot of freezing point as a function of specific energy (MJ/kg), **c** A plot of the freezing point as a function of energy density (MJ/L). Scatter points are color-coded based on density at 25 °C (g/cc) based on the colorbar located to the far right of the figure. Source data for this figure are available in Supplementary Data 2.

Our modeled freezing points for syntin and cyclopropanated myrcene were 20–30 °C higher than the experimentally determined freezing points of these reference molecules^11^. To account for this 20–30 °C error in our model and align with the freezing point requirements of kerosene-based aviation fuels (e.g., Jet-A at −40 °C, Jet-A1 at −47 °C), we selected −10 °C to −20 °C as a conservative freezing point cutoff range (red) for our study^3^. As expected, our results suggest that the freezing points of POP-FA derivatives decrease as their chain length or total carbon number decreases: alkanes derived from C14-length POP-FAs are acceptable, and C12-length POP-alkanes optimally approach the modeled freezing points of syntin and cyclopropanated myrcene (Fig. 2).

Lastly, to reduce substrate and energy consumption associated with production of sustainable POP fuels while maintaining comparable predicted energy properties to fuelimycins A-D, we reasoned that POP-FAs in the C12–C14 range would lead to more efficient fuel biosynthesis. Based on our previous model, C12:CP3-FAME’s predicted volumetric NHOC (38.9 MJ/L) is quite similar to that of fuelimycin B-FAME (C20:CP7-FAME, 39.3 MJ/L)^24^, despite that fuelimycin B biosynthesis requires input of four more NADPH and four more *S*-adenosylmethionine (SAM) cofactors. Further, our new model predicted similarly that C11:CP5 alkane’s (derived from C12:CP5 POP-FA) gravimetric NHOC (44.1 MJ/kg) is nearly identical to that of C15:CP7 alkane (44.0 MJ/kg) despite that C16:CP7 POP-FA biosynthesis requires an input of two more NADPH and two more SAM cofactors (Fig. 2). Together with the mentioned benefit of decreasing freezing point, our energy property computation further confirmed our target POP-FAs to be C12–C14 main-chain length.

### Bioprospecting POP-iPKS diversity via phylogenetic and structural analysis

As we explored additional POP-iPKS pathway homologs, we renamed the fuelimycin pathway genes in *S. albireticuli* NRRL B-1670 from *pop* to *pfl*—e.g., *pfl1* (iPKS), *pfl2* (CP), *pfl3* (KR), and *pfl4* (TE)—to better distinguish fuelimycin gene/protein homologs from other homologous POP pathways that may also produce POP-FAs. The jawsamycin pathway correspondingly contains core PKS homologs *jaw4* (iPKS)*, jaw5* (CP), and *jaw6* (KR), yet with no homologous TE gene.

**Fig. 3:**
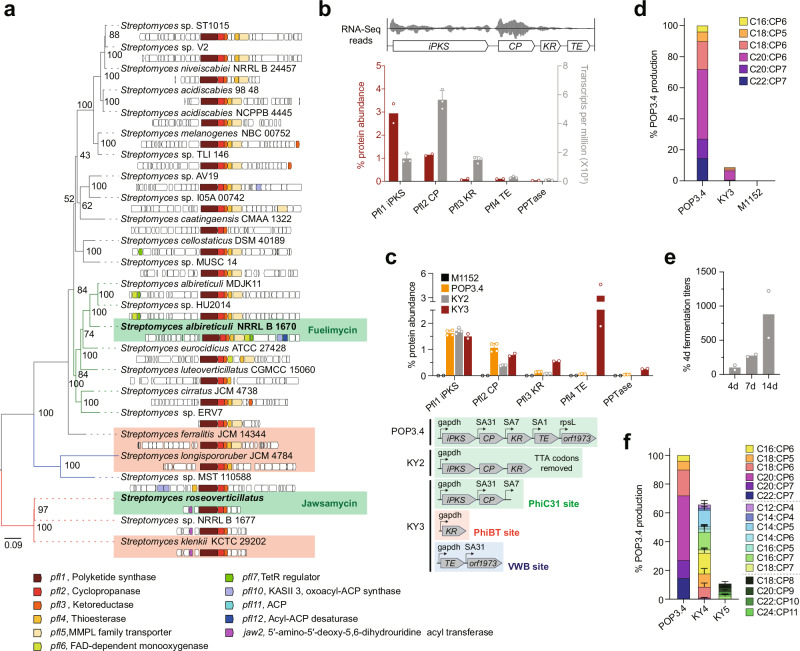
Strategies to improve POP-FA production. **a** Diversity and conservation of POP-iPKS BGCs. Pathways highlighted in green have characterized products; genes from pathways highlighted in orange were selected for characterization in this study. **b** Transcriptomic (gray, *n* = 3) and proteomic (red, *n* = 2) analysis of *pfl1-pfl4* and *orf1973* expression in POP3.4 strain. **c** Genetic reorganization of Pfl pathway and their effect on protein expression levels (M1152 and KY3, *n* = 2; POP3.4 and KY2, *n* = 4), and on **d** POP-FA production (*n* = 2). **e** Effect of fermentation time on POP-FA production (*n* = 2). **f** Production titers and diversity of POP-FAs from fuelimycin (POP3.4, *n* = 2), jawsamycin (KY4, *n* = 5) and longispororuber (KY5, *n* = 4) POP pathways. All data are presented as mean values; error bars indicate the s.d. where *n =* biological replicates and two experimental repeats, with the exception of transcriptomics (no repeats). All strain information can be found in Supplementary Data 4. Source data for this figure is available in the Source Data file; the full transcriptomics data is available in GSA under accession code CRA039551.

To diversify POP-FA chemistry and to produce our target molecules, we performed an updated phylogenomic search for pathway homologs. While our previous search covered a broader range of bacterial genomes^24^, here we used the amino acid sequences of Pfl1 and Pfl2 as queries to search through a more specialized database of 3868 actinobacterial genomes and 2502 MiBiG^55^ entries. From this approach we identified 13 additional candidate BGCs, opening further possibilities for POP biochemical diversity. While we previously identified two major clades including (1) the fuelimycin BGC and its close homologs, and (2) the jawsamycin BGC and its close homologs, our updated search revealed a third clade that includes (3) the *S. longispororuber* BGC and one other homolog (Fig. 3a). We named the core PKS homologs from the *S. longispororuber* BGC *log1* (iPKS), *log2* (CP), *log3* (KR), and *log4* (TE).

To further probe for structural clues indicating POP natural product diversification, we generated AlphaFold 3^56^ models of truncated KS-AT didomains, stand-alone CPs and KRs, and iPKS-CP-KR complexes from the Pfl, Jaw and Log pathways (Supplementary Figs. 1-2). Because of the need to investigate cyclopropanation control, we focused on AlphaFold-based structural analysis of the cyclopropanases. Phylogenetic analysis and AlphaFold 3 prediction revealed that the stand-alone CP enzyme resembles HemN, the *E. coli* oxygen-independent coproporphyrinogen III oxidase^57^ as well as a two-component system involving radical SAM enzyme C10P and methyltransferase C10Q^58^. In agreement with the HemN crystal structure, AlphaFold 3-predicted POP CP structures reveal a highly conserved N-terminal catalytic subdomain featuring an invariant “Cx_7_CxxC” motif^59^ (Jaw5 C68, C76 and C79) coordinating the 4Fe-4S cluster, and a less conserved smaller C-terminal subdomain. Interestingly however, these iPKS CPs are generally 20–40 residues longer than HemN at the C-terminus, with varying lengths across different CPs (Supplementary Fig. 3). Moreover, the diversity on both the structure and sequence (Supplementary Fig. 4)^60^ level within the POP CP family suggests that pathway-to-pathway variations in POP product programming may be at least partially encoded in the CP domain, which we sought to test in vivo.

### Characterizing natural POP product diversity by heterologous expression

#### Analyzing Pfl protein abundance effect on fuelimycin titers

In our previous study, we successfully expressed *pfl1-4* genes together with a phosphopantetheinyl transferase (PPTase) gene encoded in ORF.1973 *in Streptomyces coelicolor* M1152, making the fuelimycin-producing POP3.4 strain. However, measuring differences between POP pathways requires sufficiently stable product titers. To improve and stabilize the POP-FA titers, we first probed heterologous expression levels in strain POP3.4. While we observed expression of Pfl1 and Pfl2 at around 1–3% of the total protein abundance, proteomics and RNAseq analysis revealed much lower expression of Pfl3, Pfl4 and ORF.1973 PPTase (Fig. 3b). Natively, the *pfl1-3* genes in *S. albireticuli* NRRL B-1670 strain are likely in a single operon. As the refactoring of the pathway in strain POP3.4 may have altered the native ratio of the Pfl proteins, we decided to re-establish native protein abundance and evaluate the expression of each gene in this cryptic operon, resulting in *S. coelicolor* strain KY2. After measuring similar expression levels of *pfl1-pfl3* in both POP3.4 and KY2 (Fig. 3c), we continued with the POP3.4 refactoring strategy of pairing each gene with its own dedicated promoter, for the ease of further engineering.

Based on typical PKS domain stoichiometry^43^, we hypothesize that Pfl1 (iPKS), Pfl2 (CP), Pfl3 (KR) and Pfl4 (TE) together should form a protein complex in a 1:1:1:1 ratio. Thus, we reasoned that increasing *pfl3* and *pfl4* expression levels may lead to more functional Pfl complexes and ultimately higher POP-FA titers. Moreover, as a trans-acting TE, higher protein abundance of Pfl4 may lead to early termination of polyketide biosynthesis and shorter-chain POP-FAs. To test these hypotheses, we increased *pfl3* and *pfl4* gene copy numbers by utilizing additional integration sites and replaced underperforming promoters (Fig. 3c). Many *pfl* expression ratios were achieved with the aforementioned strategies, making *pfl3/pfl4*-overexpressing KY3 and KY6, *pfl3*-overexpressing KY7 and *pfl4*-overexpressing KY8. We then checked the POP-FA product profiles of these engineered strains.

To our surprise, we observed a universal decrease of POP-FA titers compared to POP3.4 strain (Fig. 3d; Supplementary Fig 5). This observation suggested that matching the native *pfl* expression ratio may be important for optimal heterologous POP-FA production. Furthermore, *pfl4* overexpression did not significantly alter the main-chain length distribution of POP-FAs. Alongside its distinctive feature of being a Hotdog-fold TE^24^, this result reinforces that Pfl4 behaves very differently from common stand-alone thioesterases^61^, motivating us to test alternative *pop* pathways for shortened C12-C14 POP-FA production.

#### Testing alternative hosts and cultivation media optimization

Before moving on to homologous *pop* pathway investigation, we decided to investigate common metabolic engineering strategies for POP-FA titer and production stability improvement. We recently reported that exogenous addition of pyrroloquinoline quinone (PQQ) improves production of secondary metabolites in a broad range of *Streptomyces*, as well as activates previously cryptic BGCs^62^. However, exogenous addition of PQQ^62^ did not activate *pfl1-4* in the native *S. albireticuli* NRRL B-1670 strain (Supplementary Fig 6). Furthermore, media supplementation of PQQ, glucose, and malonate^63–65^ each did not improve fuelimycin production in POP3.4 (Supplementary Fig 7).

We also tested other fast-growth *Streptomyces* chassis strains, including *S. venezuelae* ATCC 15439 and *S. albus* J1074. We observed expression of all heterologous proteins in *S. venezuelae* ATCC 15439^66,67^ (Supplementary Fig 8) yet only ~ 10% of fuelimycin production compared to POP3.4 (Supplementary Fig 9). Additionally, *pfl2, pfl3* and ORF.1973 expressed poorly in *S. albus* J1074 (Supplementary Fig 8), confirming *S. coelicolor* to be the preferred host for our study.

Finally, synthesis of polyketides and other natural products primarily occurs during a late phase of growth, after *Streptomyces* has switched to secondary metabolism^68–70^. We thus extended the cultivation time from previously reported 4 days to 7 days, and observed 1.9- to 4.4-fold increased and more consistent product titers. Further extending cultivation time to 14 days reduced consistency, but presented an additional increase in titers by 1.8- to 4.8-fold, to a total increase of 4- to 18-fold as compared to 4 day cultivations (Fig. 3e). We determined that together with optimized POP-FA extraction methods^71^, a 7-day cultivation time enabled a balance of consistent, increased titers and rapid Design-Build-Test cycles to explore other *pop* gene clusters in *S. coelicolor*, detect a wide range of POP-FA products with diverse chain length, and systematically compare product profiles despite a potentially limited titer for minor POP-FA products. Given the increased yet inconsistent titers, we reserved scaled-up 14-day cultivations for compound isolation and more detailed analysis of products from selected pathways.

#### Identifying product profiles of homologous pop gene clusters

Besides fuelimycins, POP-containing jawsamycins were also reported in *Streptomyces roseoverticillatus*^42^. Compared to major fuelimycin products, which range from C18 to C22 in main-chain length and contain 6 to 7 cyclopropane rings, the jawsamycin pathway has a maximum reported main-chain length of C18 and maximum reported cyclopropane number of 5^42^. However, the relative amounts of the jawsamycin major and minor products have not been described. Furthermore, lacking a native TE, the jawsamycin pathway does not produce any POP-FA. Therefore, to directly compare the profile of POP-FA products between jawsamycin and fuelimycin pathways, we introduced the core jawsamycin PKS genes *jaw4* (iPKS)*, jaw5* (CP), and *jaw6* (KR) together with *pfl4* (TE) and ORF.1973 (PPTase) from *S. albireticuli* into *S. coelicolor* M1152, creating strain *S. coelicolor* KY4. We refer to the polycyclopropane PKS in KY4 as JJJP with the first letter designating the iPKS, the second letter the CP, the third letter the KR, and the fourth letter the TE. J refers to *jaw* subunits, and P refers to *pfl* subunits. We then compared the POP-FA products from each *pop* pathway by LC-MS analysis.

The POP3.4 strain (PPPP) produced fuelimycins ranging from 16 to 22 carbons in main-chain length, with at least one double bond. We named them class I POP-FAs. In comparison, the jawsamycin core pathway in strain KY4 produced a shorter POP-FA profile, ranging from 12 to 18 carbons long, achieving our major engineering goal of shortening POP-FA main-chain length. The chain length differences between POP3.4 and KY4 also opened the possibility of POP-FA chain length control via a hybrid *pop* system. Moreover, unlike fuelimycins, jawsamycin has a carbon-carbon double bond between two cyclopropane rings as a result of a cyclopropanation skip in its second iterative cycle^39,42,72^ (Fig. 1a). We name the products of this modified jawsamycin pathway (JJJP), which we expect would share the skipped-cyclopropane pattern, class II POP-FAs.

We noticed that, out of all 13 POP-FAs produced by POP3.4 and KY4, only 2 from KY4 were fully cyclopropanated; these cyclopropane-saturated POP-FAs comprised less than 20% of total KY4 products, driving us to search for PKSs that would produce a higher cyclopropane saturation ratio. From our phylogenetic analysis, we identified a highly divergent POP-iPKS pathway from *Streptomyces longispororuber* (Fig. 3a). We therefore introduced the core *S. longispororuber* POP-iPKS pathway—*log1* (iPKS), *log2* (CP), *log3* (KR), and *log4* (TE)—into *S. coelicolor* M1152 creating strain KY5, harboring the LLLL PKS (L refers to *log* subunits). As a result, KY5 (LLLL) produced class III POP-FAs between 16 and 24 carbons long, all of which were fully saturated with cyclopropane rings (Fig. 3f; Supplementary Fig 10), presenting a distinct class of molecules compared to the larger number of double bonds in both fuelimycin (POP3.4) and jawsamycin (KY4) pathway products.

The cyclopropane position changes proposed in these different classes of POP-FAs were previously unreported to the best of our knowledge, and may offer a novel strategy for cyclopropane synthesis and engineering in a biological system. Our theoretical calculations suggest that alkanes derived from class II and III POP-FAs have increased specific energy compared to alkanes derived from class I POP-FAs. Additionally, class III POP alkanes achieve a comparable predicted freezing point as class II POP alkanes in one fewer round of CO_2_-emitting chain extension (Fig. 2; Supplementary Data 2). Control over the placement of alkene group(s) may also enable fine-tuning of properties, like freezing point, with downstream chemical modifications such as methylation or hydrogenation. However, class I POP-FAs in the POP3.4 strain remained the most productive: KY4 (JJJP) and KY5 (LLLL) achieved only 66% and 10% of the total product titers of POP3.4, respectively. With these diverse pathways available and with three classes of POP-FA products characterized, we next sought to investigate as well as combine the product titer, chain length, and/or cyclopropane saturation advantages of each pathway.

### Investigating POP-iPKS programming with chimeric pathways

The recombining of domains or modules from different type I multimodular PKS pathways into a chimeric PKS is a proven approach for producing novel or desired polyketides^20,22,23^. For iterative synthases, in vivo gene exchange serves the additional purpose of elucidating product programming mechanisms due to the nature of the pathway architecture^44–46,48^.

#### Engineering control of chain length

**Fig. 4:**
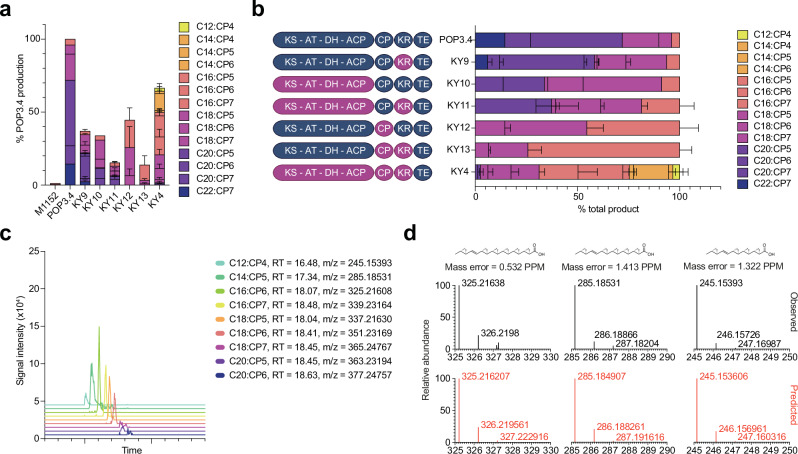
Diversification of POP-FA chain lengths. Comparisons of **a** total production and **b** chain length profile of POP-FAs from chimeric strains. Data are presented as mean values; for *n* > 2, error bars indicate the s.d. of biological replicates (*n* = 1 for M1152; *n* = 2 for POP3.4 and KY10; *n* = 3 for KY11, KY12, and KY13; *n* = 4 for KY9; *n* = 5 for KY4); two experimental repeats. **c** Extracted ion chromatograms for strain KY4 (*jaw4-jaw6*, *pfl4*; JJJP); three experimental repeats. **d** Observed (black) and predicted (red) ions of C16:CP6, C14:CP5, and C12:CP4, two experimental repeats. All strain information can be found in Supplementary Data 4. Source data for this figure is available in the Source Data file.

The fuelimycin products have on average 3–6 more total carbons than the jawsamycin products, thus opening an interesting opportunity to investigate PKS main-chain length control. To investigate the effect of each PKS domain on the chain length profile of its products, we constructed chimeric pathways in which 1-2 domains from the fuelimycin PKS (PPPP) were substituted with the corresponding jawsamycin domain (J)(Fig. 4a, b).

We first observed that KR substitution from Pfl3 to Jaw6 (strain KY9 harboring PPJP) did not significantly impact the chain length distribution of POP-FA products; similar to the native fuelimycin pathway (POP3.4), 60–70% of total products had a main-chain length of 20–22 carbons, while 20–35% of products had a main-chain length of 18 carbons.

Next, we tested replacement of the fuelimycin iPKS with the jawsamycin iPKS (strain KY10 harboring JPPP) and observed a decrease in the proportion of POP-FAs with 20 carbons (main-chain length) from 50–65% to 35–40%, an increase in abundance of C18 main chain products from 20–25% to 40–55%, and virtually no detection of C22 main chain products.

Finally, substitution of the fuelimycin CP domain with that from jawsamycin (strain KY12 harboring PJPP) resulted in a more significant decrease in main-chain length profile: approximately 55% of total POP-FAs were C18 (main chain), and the remaining 45% were C16. By comparison, the core jawsamycin pathway in KY4 (JJJP) produced the shortest profile: only 30% of total products were C18 (main chain), 45% were C16, and 25% were C14 and C12 (Fig. 4). These results support that the CP, and to a lesser extent the iPKS, are primary determinants, and thus engineering targets, for POP-FA chain length.

#### Engineering control of cyclopropane ratio

**Fig. 5:**
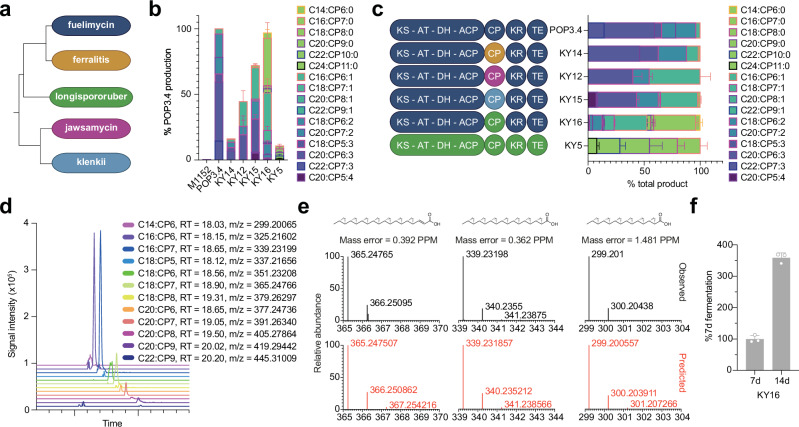
Diversification of POP-FA cyclopropanation levels. **a** Diversity of CP homologs selected for chimeric pathways. Comparisons of **b** total production and **c** cyclopropanation profile of POP-FAs from chimeric strains. To highlight cyclopropane ratios, an alternative “C[x]:CP[y]:[z]” nomenclature is used for POP-FAs, where x represents main chain carbon length, y represents the number of cyclopropane rings, and z represents the number of C = C double bonds. The data are presented as mean values; for *n* > 2, error bars indicate the s.d. of biological replicates (*n* = 1 for M1152; *n* = 2 for POP3.4 and KY14; *n* = 3 for KY12, KY15 and KY16; *n* = 4 for KY5); two experimental repeats **d** Extracted ion chromatograms for strain KY16 (*pfl1, log2, pfl3, pfl4;* PLPP); three experimental repeats. **e** Observed (black) and predicted (red) ions of C18:CP7, C16:CP7, and C14:CP6, two experimental repeats. The position of CP-rings for C18:CP7 is not determined. **f** Effect of fermentation time on POP-FA production in strain KY16 (PLPP). The data are presented as mean values; error bars indicate the s.d. of biological replicates (*n* = 3) with two experimental repeats. All strain information can be found in Supplementary Data 4. Source data for this figure is available in the Source Data file.

Having found modifications that alter POP-FA main-chain length, we next sought to engineer cyclopropane ratio. An increase in the cyclopropane ratio is desirable, as each cyclopropane ring has a strain energy of 27 kcal/mol^73^, and thus additional cyclopropane rings increase the energy density of the POP-FA molecule while replacing energetically unfavorable, oxidatively unstable double bonds. Given that the CP domain performs the cyclopropanation reaction, we reasoned that it would play a major role in determining cyclopropane ratio, alongside its demonstrated role in determining main-chain length. To test this hypothesis, from our earlier phylogenetic analysis, we selected three CP genes from *S. ferralitis* (*ferCP*), *S. klenkii* (*kleCP*), and *S. longispororuber* (*log2*) (Fig. 5a) based on evolutionary distance, substituted them into POP3.4, and confirmed their expression with proteomics analysis (Supplementary Fig. 11).

Notably in line with their evolutionary proximity, the *ferCP* substitution in strain KY14 (PFPP) resulted in a product profile resembling that of the full fuelimycin pathway (POP3.4 with PPPP), while the *kleCP* substitution in strain KY15 (PKPP) resulted in a product profile resembling that of the jawsamycin CP substitution in KY12 (PJPP). While approximately 65% of total POP-FA products from strains POP3.4 (PPPP) and KY14 (PFPP) contained 3 double bonds and 25-30% of products contained 2 double bonds, KY15 (PKPP) and KY12 (PJPP) produced a higher cyclopropane ratio, with 40% of total products containing 3 double bonds and 40-45% of total products containing 1 double bond. Finally, the *log2* CP substitution in strain KY16 (PLPP) resulted in a further increase to the cyclopropane ratio of POP-FA products, with 40% of total products containing 1 double bond and 45% being fully cyclopropanated POP-FAs (C16:CP7, C14:CP6) (Fig. 5).

Notably, KY16 (PLPP) matched the higher product titers of POP3.4, and compared to KY5 (LLLL), produced a shorter POP-FA profile with 60–70% of products having 16 carbons in the main chain. KY16 (PLPP) had a > 400% increased class III POP-FA titer (and 900% increased total titer) compared to KY5 (LLLL), further illustrating in vivo gene exchange as an efficient strategy for PKS-based POP-FA engineering. In addition to its role in chain length determination, these results demonstrate the CP domain to be a key engineering target for cyclopropanation control.

Given its combined titer, cyclopropanation, and chain length advantages, we selected strain KY16 (PLPP) for 14-day cultivations (Fig. 3f), scaled up to 3 L, for the purpose of HPLC purification and structural characterization of products. In bioreactor format, we again observed a late-stage production increase corresponding to glucose depletion^74^ (Supplementary Fig. 12); however, relative production titers remained higher when strains were cultivated in flasks. Based on our standard curves (Supplementary Table 1; Supplementary Figs. 13–14), strain KY16 produced an estimated 5.40–6.24 mg/L total POP-FAs in 50 mL flask cultures and 1.37–1.58 mg/L total POP-FAs in 1 L bioreactor cultures. Subsequent LC-MS/MS analysis resulted in fragmentation spectra that confirm the cyclopropane-saturated structures of class III POP-FAs (Supplementary Figs. 15–18); however, our MS/MS analysis confirmed only the number and not the position of carbon-carbon double bonds in unsaturated products from KY16 (PLPP) (Supplementary Figs. 19–22). In total, our engineered strains present an increase to POP-FA diversity from 6 to at least 18 distinct molecules (Table 1; Supplementary Data 3), without including potential variations in carbon-carbon double bond position.

**Table 1:** Cyclopropane-saturated (class III) POP-FAs produced in this study

Chemical Structure	Name	KY4 (JJJP)	KY5 (LLLL)	KY16 (PLPP)
	C24:CP11	–	+	–
	C22:CP10	–	+	–
	C20:CP9	–	+	+
	C18:CP8	–	+	+
	C16:CP7	+	+	+
	C14:CP6	+	–	+
	C12:CP5	–	–	+

#### POP-iPKS thioesterase does not play a major role in product programming

Next, to investigate the role of POP-TE on POP product profile, we built and tested a chimeric POP complex with fuelimycin *pfl1-pfl3* (iPKS, CP, and KR genes) and *log4* (TE), resulting in strain KY17 (PPPL). Although we previously observed that KY5 (LLLL) strictly produced fully cyclopropane-saturated POP-FAs, the KY17 (PPPL) product profile instead resembled that of POP3.4, suggesting that POP-iPKS TE domains are promiscuous and do not significantly control POP-FA product profiles (Supplementary Fig 23). This further supports that the addition of a POP-TE into the jawsamycin pathway, e.g., strain KY4 (JJJP), should produce class II POP-FAs and not modify the distinct cyclopropanation pattern of jawsamycins.

#### N-terminal subdomain of Log2 cyclopropanase exhibits product programming activity

As described above, comparison of predicted cyclopropanase structures revealed a conserved N-terminal subdomain and a variable C-terminal helix (Supplementary Fig 3). To investigate the role of each subdomain on product profile, we constructed chimeric *pfl2*-*log2* and *log2-pfl2* cyclopropanases using two different subdomain boundaries (Supplementary Fig 24), which we named L-L-P, L-P-P, P-L-L, and P-P-L, and substituted them for the cyclopropanase in the fuelimycin pathway, creating strains KY20-KY23, respectively. However, all four chimeric cyclopropanases exhibited poor protein expression, with only KY20 [P(L-L-P)PP] expressing a cyclopropanase to at least 0.35–0.5% total protein abundance (Supplementary Fig 12). Correspondingly, only KY20 produced detectable titers of POP-FAs, 55–80% of which contained a single double bond, while the remaining 20–45% were fully cyclopropanated, and all detected POP-FAs were C18 or shorter (Supplementary Fig 25). This product profile, compared to that of POP3.4, suggests that the *log2* N-terminal subdomain alone was sufficient to increase cyclopropane-saturation over the *pfl* pathway. Thus, variable residues within the otherwise conserved catalytic N-terminal subdomain of the cyclopropanase may influence the programming of this iterative pathway.

## Discussion

We previously demonstrated bioproduction of energy-dense class I POP fuels. However, the chain length and cyclopropane ratio of these POP-FAs were not ideal for aviation or rocketry applications^24^. To develop competitive biojet fuels, the iterative PKS mechanisms of POP-FA biosynthesis would need to be understood and engineered. Generally, type I iPKSs have been shown to exhibit a diversity of catalytic programming mechanisms involving the substrate or intermediate specificity of AT, KS, CP, KR, ER, TE, or other domains as well as gatekeeping activity or domain-domain interactions^75^. Our research represents a pioneering investigation into the product programming function of an iPKS CP: we discovered sequence- and structure-level diversity in the CP, and subsequently demonstrated the potential for this domain to be engineered to control the profile of released POP polyketides. We report production of the shortest and most aviation-compatible POP-FAs to date, as well as production of more energy-dense class III POP-FAs for the first time, thus diversifying POP-FAs to be more customizable towards being energy efficient, thermally stable, energy dense, and/or having a lower freezing temperature.

After comparing POP-FA product profiles between chimeric strains with different CP homologs, we discovered that similarities and differences in chain length and cyclopropanation profiles aligns with CP phylogeny. We can thus predict that uncharacterized CP homologs with closer evolutionary distance to *pfl2* and *ferCP* will likely favor longer POP-FAs with a higher degree of unsaturation. By contrast, CP homologs with closer evolutionary distance to *jaw5* or *kleCP* may favor shorter POP-FAs while those closer to *log2* CP may favor high degrees of cyclopropane saturation. Finally, as bioprospection efforts expand to mine more non-*Streptomyces* genomes for POP-iPKS BGCs, particularly divergent CPs can be prioritized for characterization, and our POP3.4-derived host microbes can serve as a suitable engineering platform.

**Fig. 6:**
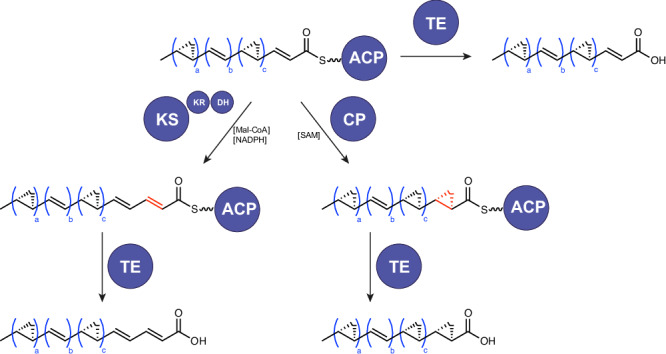
Proposed competition-driven product programming mechanism. During each extension cycle, three reactions compete for the ACP-bound intermediate. Depending on the specificity and kinetics of competing enzymes, as well as on substrate flux and likely other factors, each intermediate may undergo either a cyclopropanation-skipping extension (in which the KS outcompetes the CP and TE), a cyclopropanation (in which the CP outcompetes the KS and TE), or chain release (in which the TE outcompetes the KS and CP).

It was previously hypothesized^42^ that product programming involves a balance between condensation (KS) and cyclopropanation (CP) reaction rates (Fig. 6). The interplay between KS and CP may explain the shorter product profile of chimeric KY16 (PLPP) strain as compared to both of its native LLLL (KY5) and fuelimycin (POP3.4, PPPP) counterparts. After the 6th or 7th iterative polyketide extension, fuelimycin Pfl PKS begins to favor (i.e., exhibit faster reaction rates on) unsaturated intermediates while the *S. longispororuber* Log PKS continues to favor fully cyclopropane-saturated intermediates. The Pfl1 KS outcompetes the Pfl2 CP at those steps where unsaturations are favored. Conversely, the Log2 CP outcompetes the Log1 KS at the corresponding 6th and 7th extensions by the Log PKS, which favors cyclopropanation. In the chimeric PKS, both the Pfl1 KS and Log2 CP would act relatively fast on the 6th and 7th extensions and would no longer consistently form the same intermediates. As the intermediate preferences of a chimeric pathway diverge and conflict, condensation by the KS may stall when cyclopropane-saturated extensions occur and cyclopropanation reactions may stall when unsaturated extensions occur. These stalled intermediates could then be released as shorter POP-FAs. Intriguingly however, when increasing cultivation time to 14 days, the product profile of KY16 (PLPP) shifted with a notable increase in the longer C20:CP9 product (Supplementary Fig 26). Here, late-stage mobilization of cellular triacylglycerol (TAG)^74^ increases carbon flux towards polyketide biosynthesis; this may improve the kinetics of the Pfl1 KS reaction to outcompete the TE and perform additional extensions. Finally, the specificity of Pfl1 KS may strictly disfavor substrates longer than C20-length intermediates, as a similar increase in cultivation time to 14 days did not significantly shift the product profile of the native fuelimycin (POP3.4, PPPP) pathway (Supplementary Fig 27). If purification of these enzymatic subunits is achieved and an in vitro POP-FA production platform is established, this complex interplay of competing enzyme kinetics and substrate flux can be more deeply explored. Additionally, as more efficient protein engineering methods^76,77^ become applicable to megasynthases, the redesign of KS and CP specificities toward shorter substrates may provide the framework for stricter programming of POP-iPKS products.

Extensive pathway and host engineering must be pursued to achieve the titers, rates, and yields necessary for economically viable fuels. In particular, at least a 1000-fold increase in titers is necessary to reach g/L scale production. Although this is a significant metabolic engineering challenge, such 1000-fold titer increases^78–80^ have been previously achieved. Furthermore, heterologous production on the g/L scale has been demonstrated in *Streptomyces*^81^ as well as in other production hosts^82^.These improvements to POP production titers may also enable fuels property testing, NMR structural elucidation, and in vitro investigation and engineering of downstream enzymatic activity on purified POP-FAs. Next, strategies for enzymatic or chemical decarboxylation of POP-FAs into POP-alkenes and the following reduction to eventually give POP-alkanes, which we predict to have superior fuel properties to the current aviation fuels^24^, must be investigated.

As the light-duty transportation sector has rapidly electrified^83^, challenges remain for the development of sustainable yet energy-dense aviation, rocketry and long-haul transportation fuels. The continued development of cyclopropanated fuel molecules demonstrates that cyclopropane rings provide sought-after energy properties. Compared to organic synthesis, bioproduction of cyclopropanated fuels will minimize expenses, hazards, and greenhouse gas emissions. Additionally, organic synthesis approaches to polycyclopropane formation either depend entirely on the structure of existing olefin substrates^11,13^, or involve extensive considerations for precise structure manipulation, including the addition and removal of protecting groups, conversion or removal of *cis* vs *trans* isomers or diastereomers, and identification of chain elongation routes that do not result in cyclopropane loss^84–86^. By contrast, we have systematically shown that a POP-iPKS pathway can be engineered towards targeted chain lengths, cyclopropane saturation levels, and potentially cyclopropane positions. With the ability to precisely design cyclopropane chemistry, our work lays the foundation for the development of the highest performance jet fuels that can be biosynthesized from a sustainable carbon source.

## Methods

To identify POP BGCs, 3868 actinobacterial genomes and 2502 MiBiG^55^ entries were analyzed with a modified CORASON pipeline^24,71,87^ using the amino acid sequences of Pfl1 and Pfl2 as queries. As previously described^24^, these modifications to CORASON optimized the processing of large genome datasets, eliminated deprecated dependencies, and eliminated the trimming of sequence alignments and reformatting. The code for this modified pipeline is freely available at [https://github.com/pablo-genomes-to-vials-cruz]. *Streptomyces coelicolor* M1152 was obtained from the John Innes Centre and *S. albireticuli* NRRL B-1670 from the NRRL collection. *S. venezuelae* ATCC 15439 was obtained from ATCC. The pfl1, 2, 3, 4 and ORF.1973 genes were cloned from previously described plasmids^24^ or from genomic DNA. The *jaw4*, *5*, *6*, *ferCP*, *kleCP*, and *log2* genes were codon optimized for *Streptomyces coelicolor* using GenSmart™ Optimization and/or BaseBuddy^88,89^, and synthesized (GenScript, USA). Promoter elements were cloned from previously described plasmids^24^ and sequences are provided in Supplementary Data 6.

### Cloning and integration of plasmids

All primers and genes used in this study can be found in Supplementary Data 5-6. All plasmids were assembled using NEBuilder® HiFi DNA Assembly. The assembled plasmids were transformed into XL1-Blue (Agilent) or 5-alpha (NEB) competent *E. coli*, purified and sequenced (Plasmidsaurus, Azenta) to verify their integrity. For integration into *Streptomyces* hosts, a standard conjugation host *E. coli* ET12567 carrying a helper plasmid pUZ8002 was transformed with the verified plasmid and incubated with *Streptomyces*. Ex-conjugants were selected with the appropriate antibiotics, and propagated in SFM media. Successful plasmid integrations were confirmed by polymerase chain reaction (PCR). Additional method details can be found in previously described protocols^24,71^.

### Transcriptomics and proteomics analyses

To investigate transcription levels of the genes in the fuelimycin pathway, we lysed *Streptomyces coelicolor* M1152 POP3.4 with lysozyme at 37 °C for 45 min and extracted RNA using QIAGEN RNeasy Kit. The extracted RNA was then used for whole transcriptome sequencing with rRNA depletion (Azenta, USA). The RNA sequencing reads were mapped to the *Streptomyces coelicolor* M1152 pPOP3.4 reference genome sequence (BioProject PRJNA774980) using CLC Genomics Workbench software (CLC Bio, Aarhus, Denmark). The mapped read data were exported in BAM file format, then converted to a GFF file format to include read counts for each genomic position. The GFF file was subsequently visualized using SignalMap (v2.0.0.5; Roche NimbleGen, Basel, Switzerland).

For proteomics analysis, *Streptomyces* strains were cultured for 4 days in R5 medium with nalidixic acid (30 μg/ml) and spectinomycin (100 μg/ml) selection with 4.5 mm glass beads for mycelial dispersion. Cells were harvested and stored at −80 °C until further processing. Protein was extracted from cell pellets and tryptic peptides were prepared by following established proteomic sample preparation protocol^90^. Briefly, cell pellets were resuspended in Qiagen P2 Lysis Buffer (Qiagen, Germany) to promote cell lysis. Proteins were precipitated with addition of 1 mM NaCl and 4 x vol acetone, followed by two additional washes with 80% acetone in water. The recovered protein pellet was homogenized by pipette mixing with 100 mM ammonium bicarbonate in 20% methanol. Protein concentration was determined by the detergent compatible (DC) protein assay (BioRad, USA). Protein reduction was accomplished using 5 mM tris 2-(carboxyethyl)phosphine (TCEP) for 30 min at room temperature, and alkylation was performed with 10 mM iodoacetamide (IAM; final concentration) for 30 min at room temperature in the dark. Overnight digestion with trypsin was accomplished with a 1:50 trypsin:total protein ratio. The resulting peptide samples were analyzed on an Agilent 1290 UHPLC system coupled to a Thermo Scientific Orbitrap Exploris 480 mass spectrometer for discovery proteomics^91^. Briefly, peptide samples were loaded onto an Ascentis® ES-C18 Column (Sigma–Aldrich, USA) and were eluted from the column by using a 10 minute gradient from 98% solvent A (0.1% formic acid in water) and 2% solvent B (0.1% formic acid in acetonitrile) to 65% solvent A and 35% solvent B. Eluting peptides were introduced to the mass spectrometer operating in positive-ion mode and were measured in data-independent acquisition (DIA) mode with a duty cycle of 3 survey scans from m/z 380 to m/z 985 and 45 MS2 scans with precursor isolation width of 13.5 m/z to cover the mass range. DIA raw data files were analyzed by an integrated software suite DIA-NN^92^. The databases used in the DIA-NN search (library-free mode) was *Streptomyces coelicolor* latest Uniprot proteome FASTA sequences plus the protein sequences of the heterologous proteins and common proteomic contaminants. DIA-NN determines mass tolerances automatically based on first pass analysis of the samples with automated determination of optimal mass accuracies. The retention time extraction window was determined individually for all MS runs analyzed via the automated optimization procedure implemented in DIA-NN. Protein inference was enabled, and the quantification strategy was set to Robust LC = High Accuracy. Output main DIA-NN reports were filtered with a global FDR = 0.01 on both the precursor level and protein group level. The Top3 method, which is the average MS signal response of the three most intense tryptic peptides of each identified protein, was used to plot the quantity of the targeted proteins in the samples^93,94^. The generated mass spectrometry proteomics data have been deposited to the ProteomeXchange Consortium via the PRIDE partner repository with the dataset identifier PXD057441^95^. DIA-NN is freely available for download from https://github.com/vdemichev/DiaNN.

### Streptomyces cultivation, POP-FA extraction, LC-MS and LC-MS/MS analysis

Routine cultivation, spore collection, conjugation, and/or protoplast transformation of *Streptomyces* were performed using standard methods^96^. A comprehensive protocol for POP-FA production, extraction, purification, LC-MS analysis has been previously reported^24,71^; protocol modifications are highlighted in the following sections.

For POP-FA production, *Streptomyces* strains were first cultivated in 3 mL TSB medium^71^ with nalidixic acid (30 μg/ml) and spectinomycin (100 μg/ml) selection in 24-deep-well plates with 4.5 mm glass beads for mycelial dispersion for 3–4 days. 1 mL of each seed culture was then inoculated into 50 mL R5 medium with nalidixic acid (30 μg/ml) and spectinomycin (100 μg/ml) selection in 250 mL Erlenmeyer baffled flasks with 4.5 mm glass beads and cultivated for 4–7 days. For bioreactor experiments, three 2 L (1.8 L max working volume) Eppendorf DASGIP bioreactors were used, each at a starting volume of 1 L. A 50% Dissolved Oxygen cascade setpoint was used, utilizing agitation stir rate changes from 300 to 1300 rpm with a Rushton Impeller. An airflow rate of 30 SL/h was maintained. The DASGIP MX4×4 module controlled the airflow of sterile dry air. Sampling involved discarding the dead-space of the dip-tube sample port, followed by drawing a 30 mL sample. The setpoint for pH was 7 with a deadband range of 0.2; both acid (H_2_SO_4_ 15%) and base (NH_4_OH 15%) were controlled using the DASGIP MP8 modules.

The POP-FA products were obtained from the *Streptomyces* mycelium, which was lysed by sonication. The sonication pulse settings were modified to 15 s on/15 s off. The pH of the lysate was adjusted to 4, and methanol and chloroform were added to the lysate in a 1:1:1 ratio. The methanol:chloroform:lysate mixture was then vortexed for 10 min, followed by 5 min of centrifugation at <1000 ×* g* to ensure complete separation of fractions. The chloroform fraction was recovered, and the solvent evaporated. The remaining dense liquid product was resuspended in methanol, vortexed for 10 min and incubated at 20 °C for 8–12 h before solids were separated by centrifugation and removed. These crude extracts were analyzed using the previously reported LC-MS method^24^; in brief, a 1200 series LC system (Agilent) with a Viva C4 column (Restek, catalog number 9512511) was connected to an LTQ-Orbitrap-XL mass spectrometer with an electrospray ionization source (Thermo Fisher Scientific) and operated in negative ion mode. The resulting data was analyzed using Thermo Xcalibur 2.0.6 Qual Browser software. Using culture extracts of *S. coelicolor* M1152 (no POP-FA production) as a negative control, the abundance of each POP-FA product was calculated by their extracted-ion chromatogram (EIC) peak area; because minor products do not impact total titer nor product profile estimates as significantly as major products, we accepted a rough estimate of minor product abundances when their EICs were more complex or contained split peaks.

For LC-MS/MS analysis of KY16 (PLPP) products, POP-FA were separated using reverse phase chromatography and detected using a Thermo Orbitrap Exploris 120 mass spectrometer; all optimized parameters are available in Supplementary Data 7. Raw datafiles were converted to mzmL format using ThermoRawFileParser (version 1.4.2)^97^. MS1 and MS2 data were extracted in R using the RaMS package^98^.

### Physical property prediction methods

The physical properties predicted in this paper are density, enthalpy of vaporization, specific energy, energy density, and freezing point. Density and enthalpy of vaporization were calculated using the thermo python module^99^, which contains an ordered list of methods for enthalpy of vaporization and density, respectively (ordered based on accuracy). Many of the methods available require thermodynamic parameters, such as Tc, Pc, Vc, and ⍵. A script was written to calculate these parameters using the Joback method^100^. The values of these parameters were then made available to the methods which require them. The ordered list of methods were called iteratively using a loop, until a method was found that had available all the necessary parameters to calculate the target thermodynamic property (either enthalpy of vaporization or density).

In order to calculate the specific energy or the energy density, the enthalpy of combustion must first be calculated. This was done by first optimizing the geometry of each molecule using the B3LYP/6-311 G** method^101,102^. Subsequent frequency calculations were then carried out at the same level and theory so as to ensure that the optimized geometry is a minimum (a requirement fulfilled by ensuring that all frequencies are real and positive). B3LYP was developed in 1993 and has enjoyed wide success in predicting accurate molecular geometries, even for strained ring systems^103–106^. While the basis set chosen can have an effect on molecular geometry, research indicates that, in most cases, the choice of basis set has only a small impact on molecular geometry^107,108^. The 6-311 G** basis set is a split valence triple ζ basis set, and was chosen as a compromise between efficiency and accuracy.

A single point calculation was then carried out using the composite method CBS-QB3^109^, which provides enthalpies that are accurate to within a few kilojoules/mole^110^. Similar calculations were performed for O_2_(^3^Σ^-^_g_), CO_2_, and H_2_O. With all enthalpies calculated, and knowledge of the coefficients of the balanced combustion equation, the enthalpy of combustion was obtained. A small correction is required to account for the vaporization of each target molecule, which can be included by calculating the enthalpy of vaporization for each target molecule. Aside from the enthalpy of vaporization, all of the calculations necessary to obtain the enthalpy of combustion were performed using the Gaussian 09 quantum chemistry software^111^. Using this method, the enthalpy of combustion is obtained in units of kJ/mol. In order to obtain the specific energy in units of MJ/kg, the enthalpy of combustion must be divided by the molecular weight of the target molecule in units of kg/mol, and the result must be divided by 1000 (the moles cancel out, and kJ are converted to MJ). From the specific energy (units in MJ/kg), the energy density can be obtained by multiplying the specific energy by the density in units of kg/L (which is equivalent to units of g/cc). For syntin, the literature density of 0.851 g/cc was used^11^, although the calculated density of 0.905 g/cc represents an error of only 6.34%. For cyclopropanated myrcene, there is only a literature density at 20 °C, so a calculated density at 25 °C was used.

### Freezing point model

A freezing point model was previously obtained by training a machine learning (ML) model^52^ using a database of 19,811 freezing points^53^. Here, a brief description of how the model was developed is given. Chemical descriptors were obtained using 2D PaDel descriptors^54^. 2D descriptors provide topological information, and in the training of the freezing point model, this resulted in 1263 features. Several methods were used for training, including linear regression, ensemble-based regression, gradient boosted regression, extreme gradient boosted regression, and deep learning. The best initial performance was obtained when no feature engineering was performed, and when the extreme gradient boosted regression method was used to train the model. Performance was evaluated by calculating the mean absolute error using fivefold cross validation. The best performing model was refined by removing outliers from the training set. Outliers were identified by using the z-score method, which removed 844 freezing points from our training set. The best performing model was one with a mean absolute error of 23.69 °C.

### Evaluating the performance of the initial freezing point model against cyclopropane oligomers

Although the initial freezing point model has an excellent mean absolute error, it is important to evaluate its performance against the type of molecules produced in this work. Supplementary Table 1 shows some notable comparisons of the initial freezing point model against data taken from the available literature. The model performs well when a cycloalkane unit is skipped in the polymerization step. This is shown in entry one of the table, where the measured freezing point is 11.5 °C, and the initial freezing point model yields an estimate of 11.7 °C. However, the model’s performance is severely degraded when there are no cycloalkane units skipped. This is shown in entry 2 of the table, where the measured freezing point is 18.85 °C, and the initial freezing point model yields an estimate of 52.08 °C . Furthermore, entry 3 and 4 of the table, which are examples of cyclopropane trimers, clearly shows a degraded ability for the initial freezing point model to provide reasonable estimates of the freezing point for the molecules being produced in this work.

In order to improve the performance of our freezing point model, we retrained the model, inserting 37 additional freezing points into the training set. The structures and freezing points of the additional freezing points are available in Supplementary Data 1. The retrained model performs much better than the original freezing point model, as seen from the last column of Supplementary Table 2. The retrained model predicts a freezing point for 1,1’:2’,1”-tercyclohexane (entry 2 in Supplementary Table 2) of 5.5 °C vs. 52.08 °C using the initial freezing point model. The retrained model also does better at estimating the freezing point of syntin (entry 3 in Supplementary Table 2), as well as the cyclopropanated myrcene molecule (entry 4 in Supplementary Table 2).

### Statistics & reproducibility

No experimental repeats were performed for transcriptomics analysis or bioreactor experiments. Two or more experimental repeats were performed for proteomics analysis and for comparisons of POP-FA production titers and profiles. The quantity of the targeted proteins in the cell culture samples was plotted using the average mass spectrometry signal response of the three most intense tryptic peptides of each identified protein (Top3 method); less intense peptides were not included in protein quantitation analysis. No other data were excluded from the analyses. No statistical method was used to predetermine sample size.

### Reporting summary

Further information on research design is available in the Nature Portfolio Reporting Summary linked to this article.

## Supplementary information

Supplementary Information

Description of Additional Supplementary Files

Supplementary Data 1

Supplementary Data 2

Supplementary Data 3

Supplementary Data 4

Supplementary Data 5

Supplementary Data 6

Supplementary Data 7

Supplementary Data 8

Reporting Summary

Transparent Peer Review file

## Source data

Source Data

## Data Availability

The DNA sequences of the fuelimycin BGC from *Streptomyces albireticuli* NRRL-B1670 are available at MiBig as BGC0002138 with Protein IDs PAU45552.1 (*pfl1*), PAU45553.1 (*pfl2*), PAU45554.1 (*pfl3*), and PAU45555.1 (*pfl4*). Strains and plasmids used in this study, and listed with JPUB_IDs in Supplementary Data 4, are available upon request through the Joint BioEnergy Institute Strain Registry [https://public-registry.jbei.org/folders/946]. The RNAseq data generated in this study have been deposited in the Genome Sequence Archive^112^ in National Genomics Data Center^113^, China National Center for Bioinformation / Beijing Institute of Genomics, Chinese Academy of Sciences, under the GSA accession code CRA039551. The protein mass spectrometry data have been deposited to the ProteomeXchange Consortium via the PRIDE partner repository with the dataset identifier PXD057441. The processed LC-MS data generated in this study are provided in the Source Data file and Supplementary Data 8. Protein structures used in this work include PDB IDs 4MZ0, 5BP1, 7CPX, 1OLT. Source data are provided with this paper.

## Code availability

The ML model^52^ for freezing point predictions will be detailed in the final published version of the referenced manuscript, which is currently a preprint at the time of writing [10.26434/chemrxiv-2024-0jfmb], while data used to re-train this model is provided in Supplementary Data 1. The code for this model is accessible at DOE CODE repository^114^: [10.11578/dc.20260318.3].
