# Supplementary material for: Polyketide synthase-based controlled synthesis of polycyclopropanated fuel molecules
Source: Nat Commun. 2026 May 27;17:6904. doi: 10.1038/s41467-026-73172-3 (PMC13389473; doi:10.1038/s41467-026-73172-3)
Supplement: Supplementary file 1 — Supplementary Information [file 41467_2026_73172_MOESM1_ESM.pdf]

Table of Contents

Supplementary Figure 1. Comparison and alignment of KS-AT domains ___________________ 3
Supplementary Figure 2. Predicted structure of Jawsamycin iPKS-CP-KR complex __________ 4
Supplementary Figure 3. Predicted structures of POP CPs 4 _____________________________ 5
Supplementary Figure 4. Profile of evolutionarily conserved CP amino acid residues _________ 6
Supplementary Figure 5. Effect of genetic organization of Pfl pathway on POP-FA production _ 7
Supplementary Figure 6. Effect of exogenous pyrroloquinoline quinone (PQQ) addition on native pop protein expression ____ 8
Supplementary Figure 7. Effect of exogenous additives on fuelimycin production ____________ 9
Supplementary Figure 8. Effect of host strain on fuelimycin protein expression ______________ 9
Supplementary Figure 9. Effect of host strain on fuelimycin production ___________________ 10
Supplementary Figure 10. Mass spectra of cyclopropane-saturated POP-FAs______________ 10
Supplementary Figure 11. Relative abundance of POP-iPKS proteins from chimeric strains__ 11
Supplementary Figure 12. POP-FA production and glucose consumption in bioreactors _____ 11
Supplementary Figure 13. Arachidonic acid standard curve ____________________________ 12
Supplementary Figure 14. DCP-LA standard curve ___________________________________ 12
Supplementary Figure 15. Structural prediction of C20:CP9 using LC-MS/MS _____________ 13
Supplementary Figure 16. Structural prediction of C18:CP8 using LC-MS/MS _____________ 13
Supplementary Figure 17. Structural prediction of C16:CP7 using LC-MS/MS _____________ 14
Supplementary Figure 18. Structural prediction of C14:CP6 using LC-MS/MS _____________ 14
Supplementary Figure 19. Partial structural prediction of C20:CP8 using LC-MS/MS________ 15
Supplementary Figure 20. Partial structural prediction of C18:CP7 using LC-MS/MS________ 15
Supplementary Figure 21. Partial structural prediction of C16:CP6 using LC-MS/MS________ 16
Supplementary Figure 22. Partial structural prediction of C14:CP5 using LC-MS/MS________ 16
Supplementary Figure 23. Role of POP-TE on pathway product profile ___________________ 16
Supplementary Figure 24. Alignment of Pfl2 and Log2 and design of chimeric cyclo-propanases ____ 17
Supplementary Figure 25. POP-FA production profile from KY20 P(L-L-P)PP strain ________ 18
Supplementary Figure 26. Extracted ion chromatograms for PLPP (KY16) products at 7- and 14-day fermentations ____18
Supplementary Figure 27. Extracted ion chromatograms for PPPP (POP3.4) products at 7- and 14-day fermentations ____19
Supplementary Figure 28. POP-FA production titers and profile from JLJP (KY18) strain ____ 19
Supplementary Figure 29. Extracted ion chromatograms for LLLL (KY5) products __________ 20
Supplementary Figure 30. Extracted ion chromatograms for PPJP (KY9) products _________ 20
Supplementary Figure 31. Extracted ion chromatograms for JPPP (KY10) products ________ 20
Supplementary Figure 32. Extracted ion chromatograms for JPJP (KY11) products _________ 21
Supplementary Figure 33. Extracted ion chromatograms for PJPP (KY12) products ________ 21
Supplementary Figure 34. Extracted ion chromatograms for PJJP (KY13) products _________ 21
Supplementary Figure 35. Extracted ion chromatograms for PPPL (KY17) products ________ 22
Supplementary Figure 36. Extracted ion chromatograms for JLJP (KY18) products _________ 22
Supplementary Figure 37. Extracted ion chromatograms for P(L-L-P)PP (KY20) products ___ 22

Supplementary Table 1. Estimation of POP-FA titers using reference molecules ___________ 23
Supplementary Table 2. Evaluation of the performance of the initial freezing point model against measured freezing points ____ 23
Supplementary References ____ 24

Supplementary figures

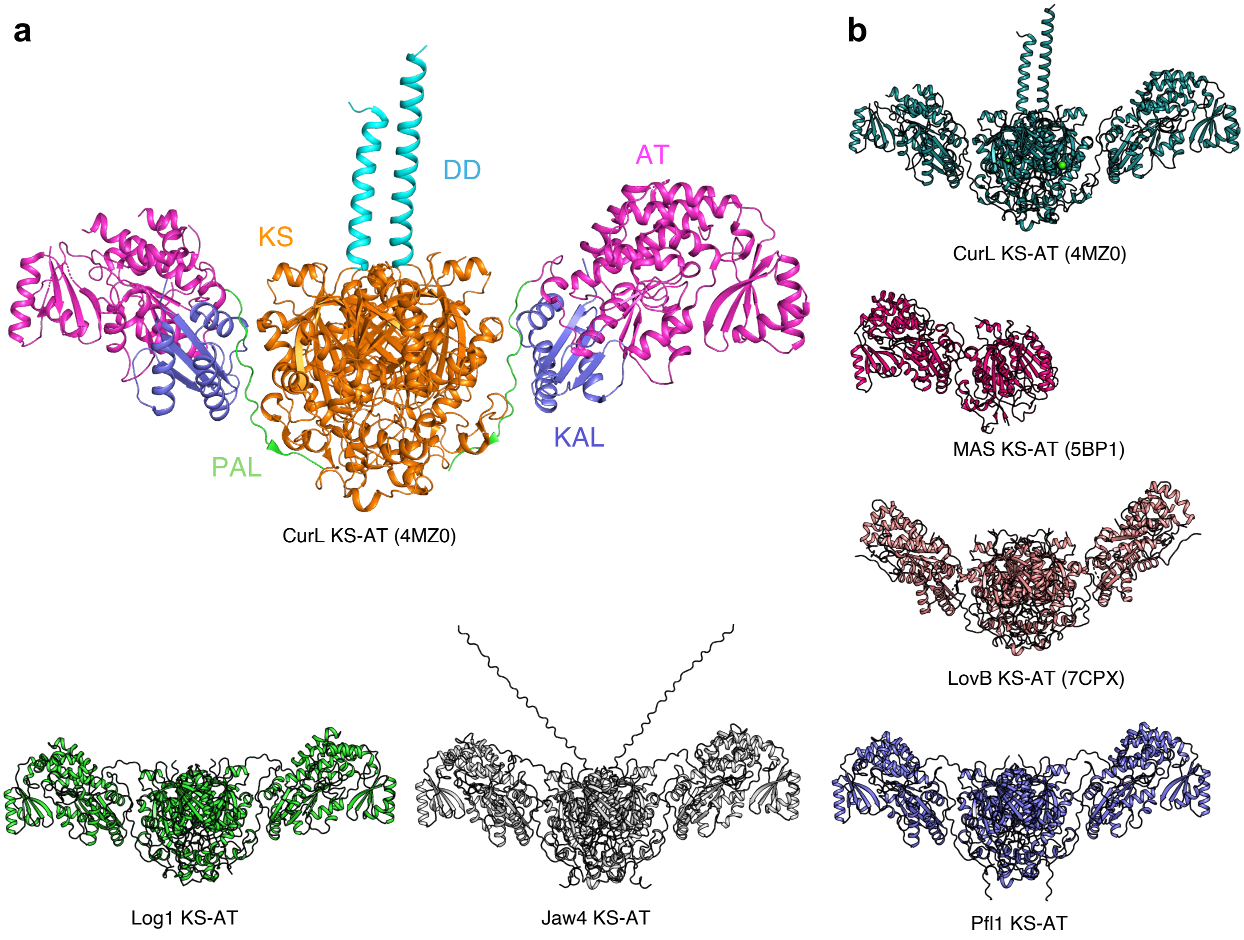

Supplementary Figure 1. Comparison and alignment of KS-AT domains. a, KS-AT didomain structure from the curacin (CurL) PKS pathway, with ketosynthase (KS), acyltransferase (AT), KS-AT linker (KAL), post-AT linker (PAL), and docking (DD) domains/regions color coded and labelled. **b,** AlphaFold 3 predictions of the Pfl1, Jaw4, and Log1 KS-AT domains were aligned and compared with KS-AT domains from the CurL, mycocerosic acid (MAS), and lovastatin (LovB) PKS pathways. All three predicted iPKS KS-AT didomain structures have a canonical extended conformation (Herbst et al. 2016), in agreement with observed relatively high conformational conservation across different clades of type I PKSs. Notably, the first 30 residues of Jaw4 iPKS are predicted to resemble a docking domain (DD, MDSLGRIHPAGGDDVRRHRRRRTRRNHRRS sequence in Jaw4) absent in the Pfl or Log pathway, suggesting possible evolution from a modular PKS ancestor.

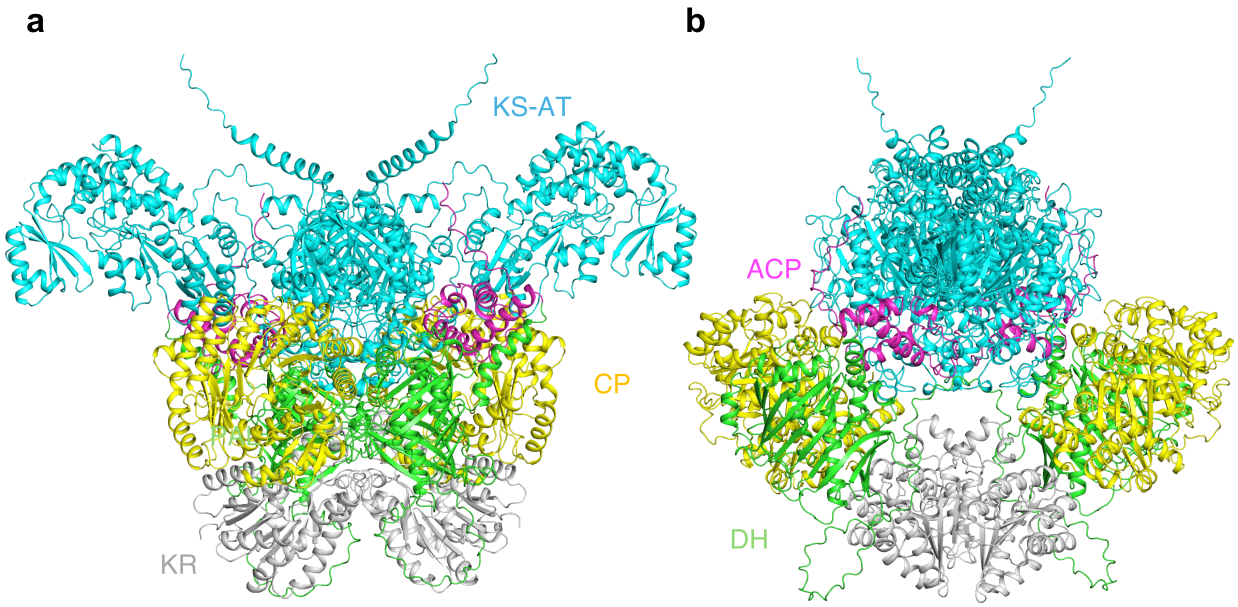

Supplementary Figure 2. Predicted structure of Jawsamycin iPKS-CP-KR complex. Downstream of the condensing region sits the POP-iPKS unique modifying region composed of the iPKS DH, iPKS ACP, stand-alone CP and stand-alone KR. AlphaFold 3 successfully predicted the iPKS-CP-KR complex structure from jawsamycin PKS, with CP predicted to directly interact with adjacent KR. Two macromolecules of CPs and KRs are recruited in close proximity to the iPKS DHs, together forming a modifying region and a cavity between the upstream condensing region and the downstream modifying region, where the iPKS ACP can gain access to all the catalytic domains. **a,b,** Two orthogonal views of the same protein structure, with **b** rotated 90° around the y-axis relative to **a**.

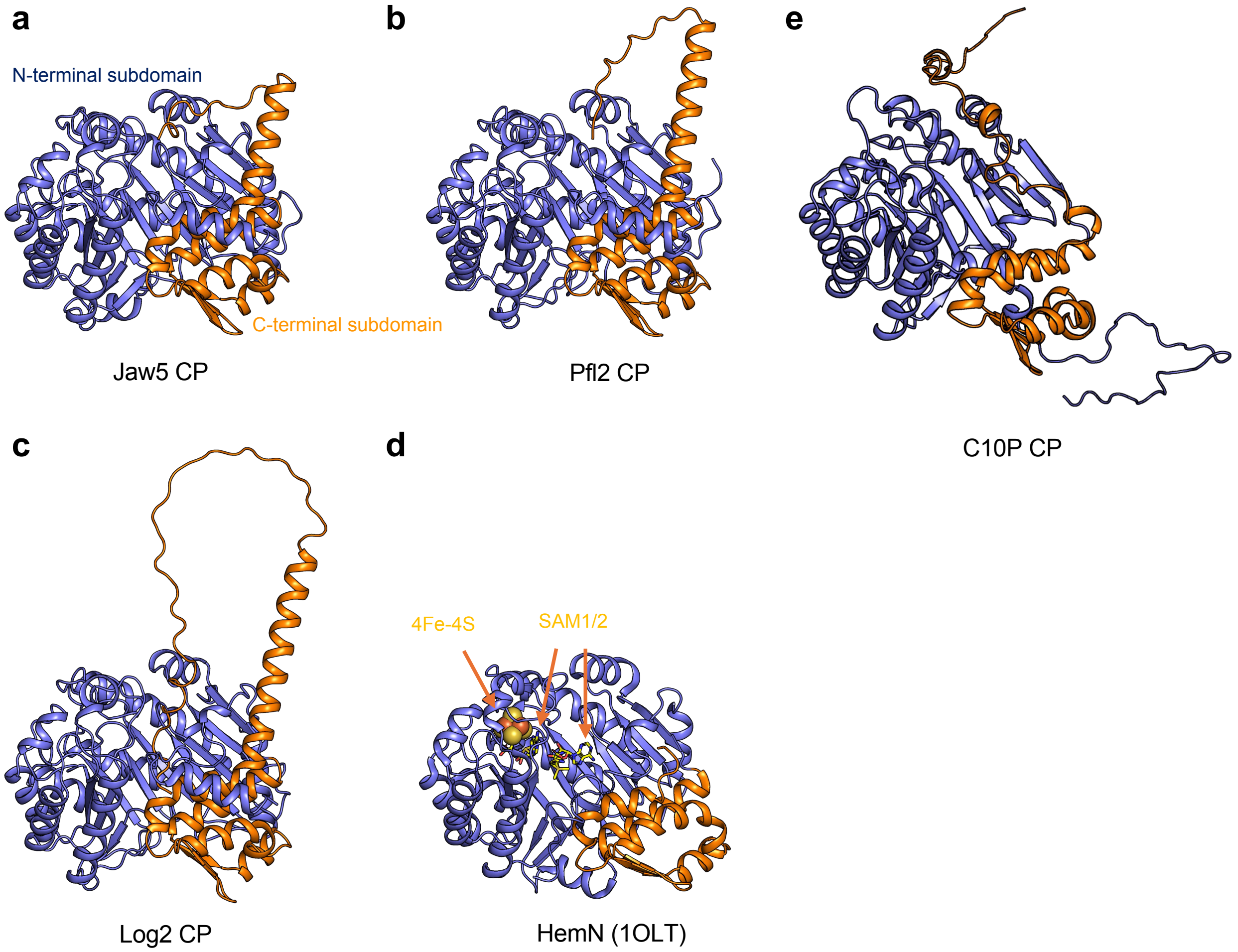

Supplementary Figure 3. Predicted structures of POP CPs. AlphaFold 3 predictions of **a,** Jaw5, **b,** Pfl2, and **c,** Log2 were compared with **d,** HemN oxygen-independent coproporphyrinogen III oxidase from *E. coli*. The C-terminal subdomain of POP CPs are notably longer than that of HemN. Alphafold 3 prediction of **e,** C10P, another CP from the HemN-like family of radical SAM enzymes from *Streptomyces zelensis* TG1402, is included for comparison.

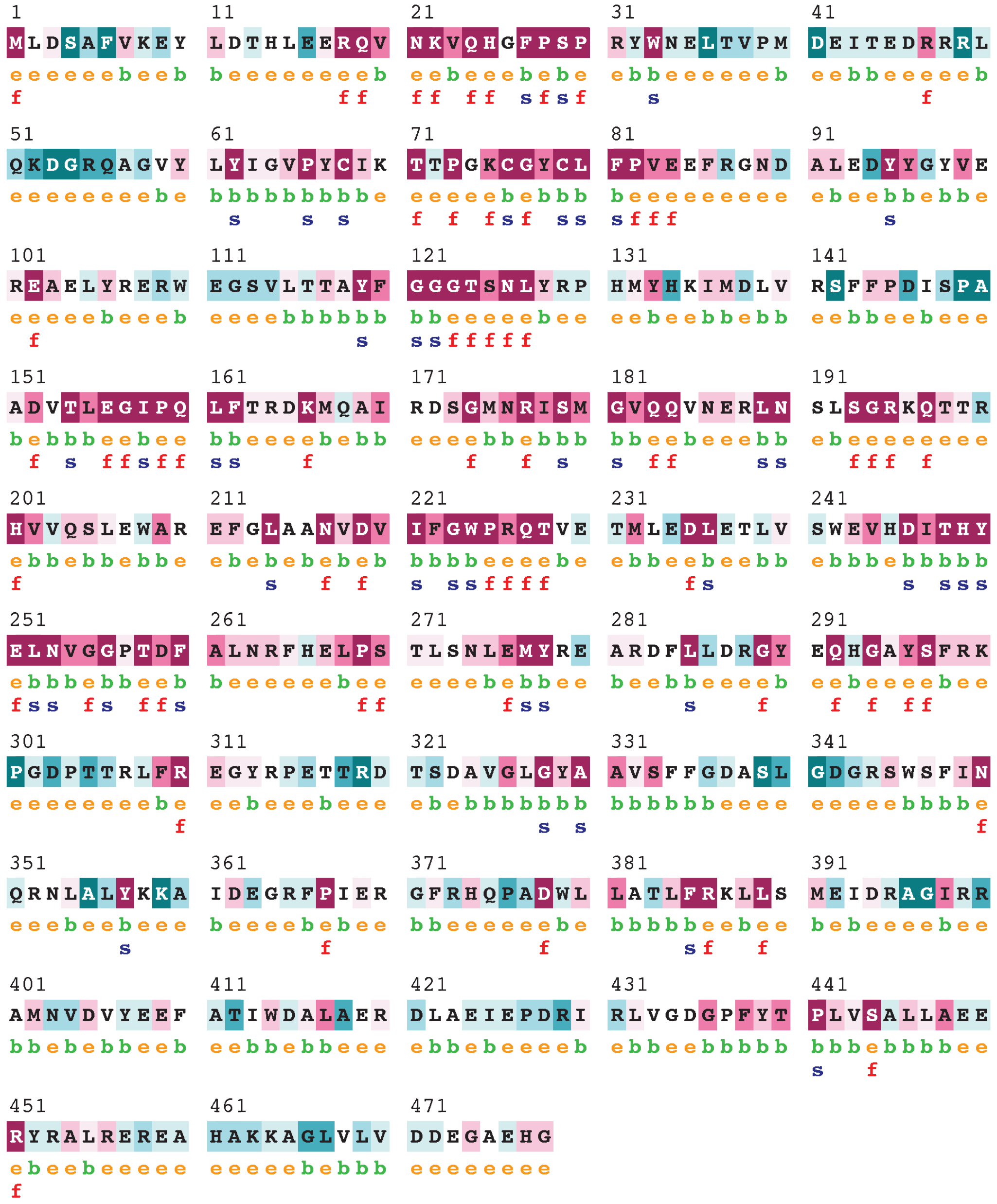

The conservation scale:

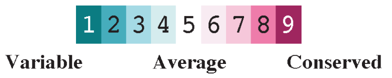

e - An exposed residue according to the neural network algorithm.

b - A buried residue according to the neural network algorithm.

f - A predicted functional residue (highly conserved and exposed).

s - A predicted structural residue (highly conserved and buried).

x - Insufficient data - the calculation for this site was performed on less than 10% of the sequences.

Supplementary Figure 4. Profile of evolutionarily conserved CP amino acid residues. ConSurf profile of 30 homologous POP-CP domains reveals a highly conserved N-terminal region. The reference sequence of Jaw5 CP is shown. The variable C-terminal region may be important for iPKS interaction and substrate recognition.

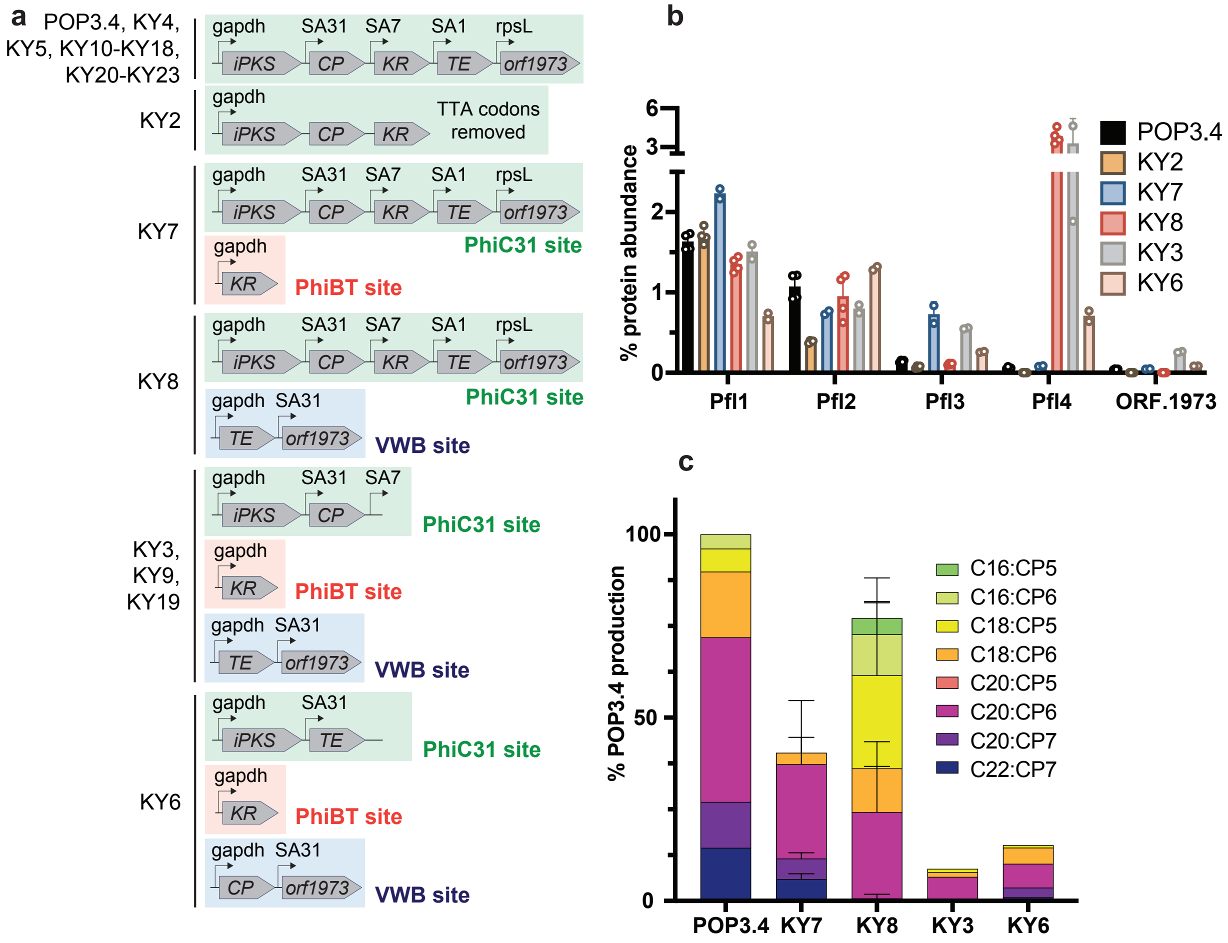

Supplementary Figure 5. Effect of genetic organization of Pfl pathway on POP-FA production. a, To test the effect of *pfl* expression levels on POP-FA production, we increased gene copy number, utilized secondary and tertiary integration sites, and optimized promoter/RBS selection. **b,** The resulting strains exhibited increased expression of Pfl3 and/or Pfl4 (*n* = 2 for KY7, KY3 and KY6; *n* = 4 for POP3.4, KY2, and KY8 where *n* = biological replicates). **c,** POP3.4 remained the most productive strain for POP-FAs (*n* = 2 for POP3.4, KY3 and KY6; *n* = 3 for KY8; *n* = 4 for KY7 where *n* = biological replicates). All data are presented as mean values; where *n* > 2, error bars indicate the s.d. of biological replicates. All strain information can be found in Supplementary Data 4. Source data for this figure are provided in the Source Data file.

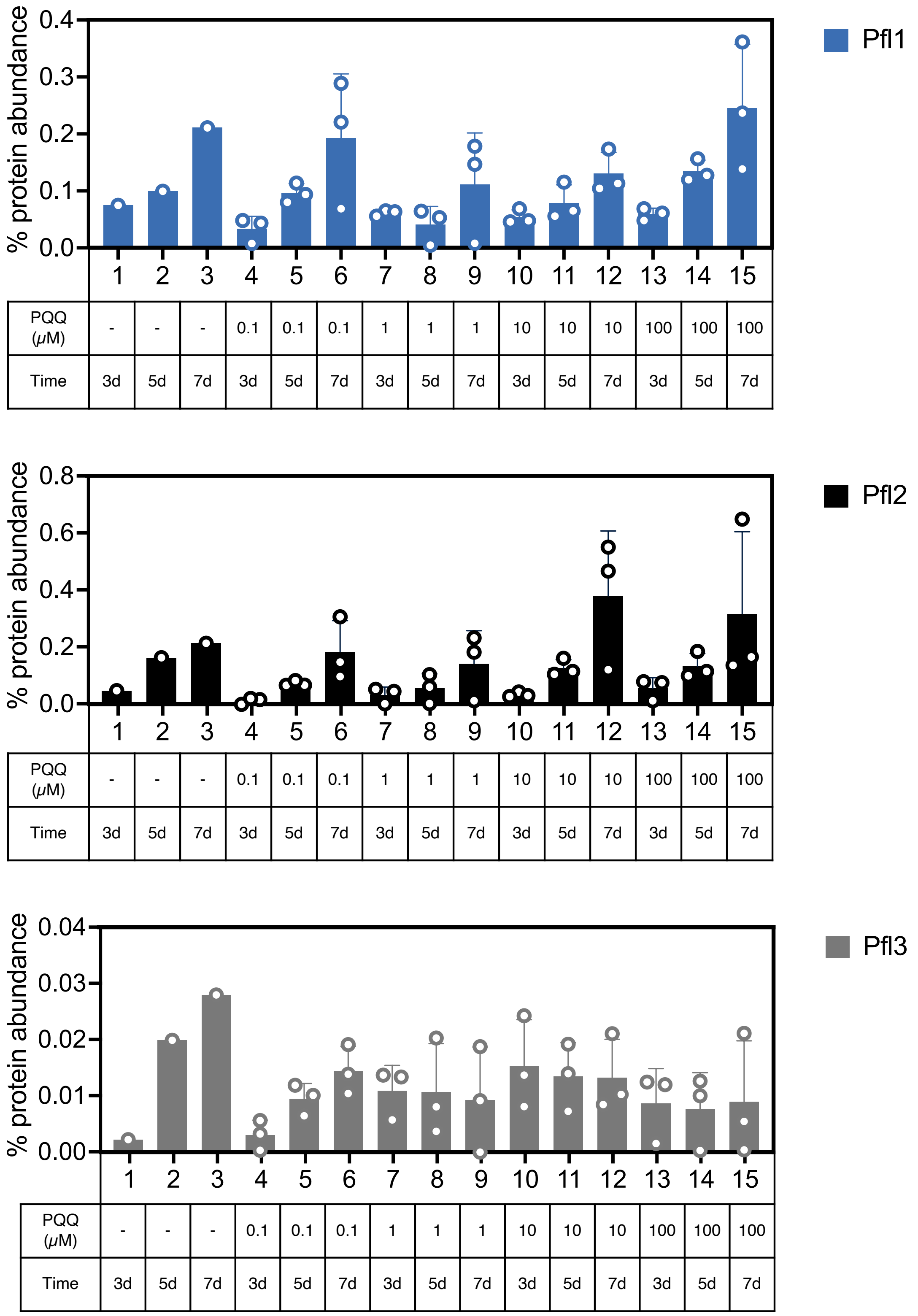

Supplementary Figure 6. Effect of exogenous pyrroloquinoline quinone (PQQ) addition on native pop protein expression. Relative abundance of Pfl1-3 proteins in *S. albireticuli NRRL* B-1670. Varying concentrations of PQQ (uM) were added, and protein expression was measured at different time points (3, 5, 7 days). All data are presented as mean values; where *n* > 2, error bars indicate the s.d. of biological replicates (*n* = 3 for all conditions except for the control with no PQQ addition, where *n* = 1). Source data for this figure are provided in the Source Data file.

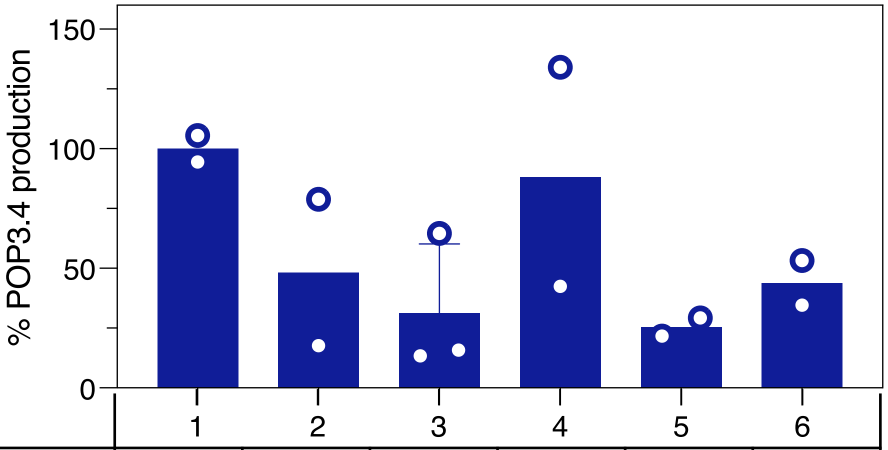

	1	2	3	4	5	6
Glucose	1%	1%	1%	4%	1%	1%
Malonate	-	20 mM	50 mM	-	-	-
PQQ	-	-	-	-	0.04 μM	0.4 μM

Supplementary Figure 7. Effect of exogenous additives on fuelimycin production. Total POP-FA production in *S. coelicolor* M1152 (POP3.4). Different concentrations of glucose, malonate, and PQQ were tested. All data are presented as mean values; where *n* > 2, error bars indicate the s.d. of biological replicates (*n* = 3 for 50 mM malonate condition; *n* = 2 for other groups). Source data for this figure are provided in the Source Data file.

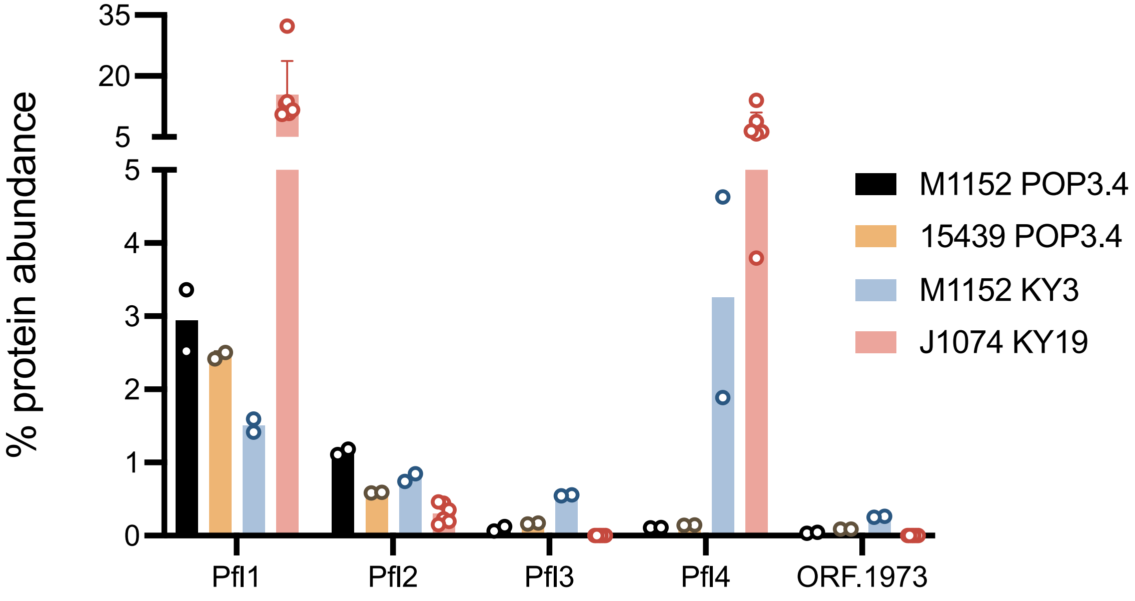

Supplementary Figure 8. Effect of host strain on fuelimycin protein expression. Relative protein abundance of *pfl*1-*pfl*4 and ORF.1973 in *Streptomyces* hosts: *S. coelicolor* M1152, *S. venezuelae* ATCC 15439, *S. albus* J1074. POP3.4 strains contain the full pathway in a single integration site; KY3 strains contain Pfl1, Pfl2 in a first integration site, Pfl3 in a second integration site, and Pfl4, ORF.1973 PPTase in a third integration site. All data are presented as mean values; where *n* > 2, error bars indicate the s.d. of biological replicates (*n* = 2 for M1152 POP3.4, 15439 POP3.4, M1152 KY3, and *n* = 6 for J1074 KY19). All strain information can be found in Supplementary Data 4. Source data for this figure are provided in the Source Data file.

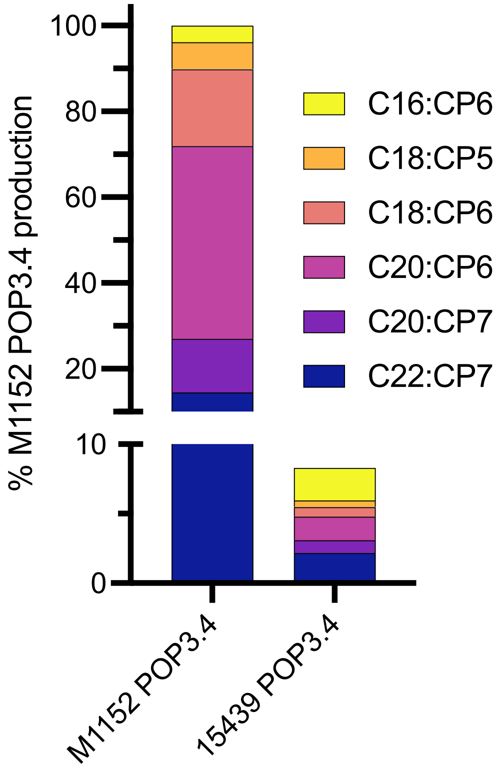

Supplementary Figure 9. Effect of host strain on fuelimycin production. Total POP-FA production in *Streptomyces venezuelae* ATCC 15439 POP3.4 compared to *S. coelicolor* M1152 POP3.4. All data are presented as mean values of biological replicates (n = 2). Source data for this figure are provided in the Source Data file.

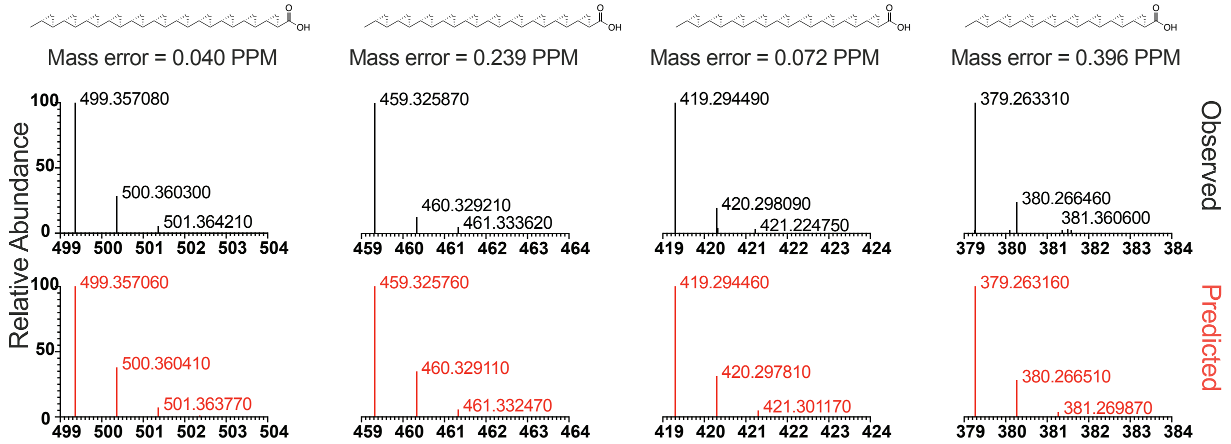

Supplementary Figure 10. Mass spectra of cyclopropane-saturated POP-FAs. Observed and predicted ions of C24:CP11, C22:CP10, C20:CP9 and C18:CP8 from LLLL (KY5) strain. All strain information can be found in Supplementary Data 4. Source data for this figure are provided in the Source Data file.

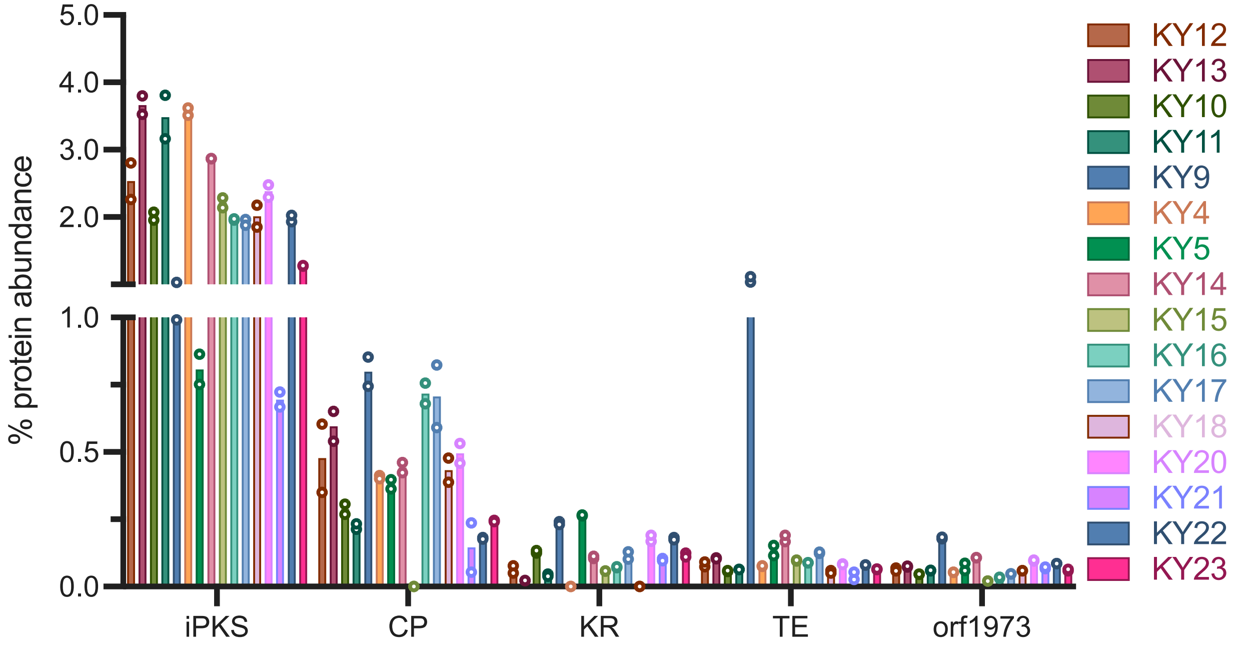

Supplementary Figure 11. Relative abundance of POP-iPKS proteins from chimeric strains. To characterize natural and chimeric pathway product diversity, the POP3.4 gene organization was used, with the exception of strain KY9 (PPJP) which used the KY3 gene organization with secondary and tertiary integration sites. iPKS pathway protein expression levels were consistent with those of POP3.4 and KY3, respectively. Depending on the chimeric strain, the iPKS protein was Pfl1/Jaw4/Log1; the CP protein was Pfl2/FerCP/Jaw5/KleCP/Log2, the KR protein was Pfl3/Jaw6/Log3, and the TE protein was Pfl4/Log4. All data are presented as mean values of biological replicates (n = 2). All strain information can be found in Supplementary Data 4. Source data for this figure are provided in the Source Data file.

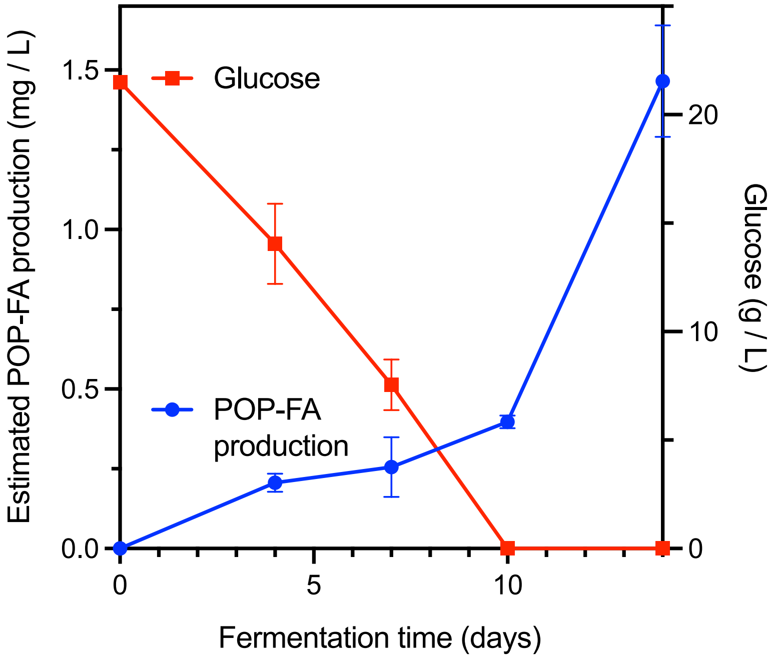

Supplementary Figure 12. POP-FA production and glucose consumption by strain KY16 (PLPP) in bioreactors. Although cell growth was not accurately measured via OD_{600} due to flocculation, *Streptomyces coelicolor* likely reached stationary phase by day 4, during which POP-FA production temporarily stagnated. After glucose depletion and a corresponding switch towards secondary metabolite production (Wang et al. 2019), POP-FA production increases again. All data are presented as mean values; error bars indicate the s.d. of biological replicates (n = 3). Source data for this figure are provided in the Source Data file.

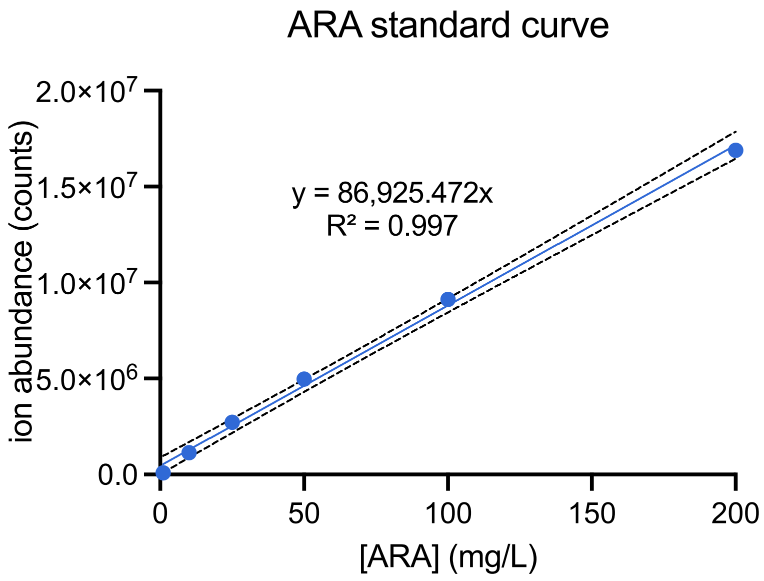

Supplementary Figure 13. Arachidonic acid standard curve. Varying concentrations of arachidonic acid from 10-200 mg/L were measured for the purpose of estimating POP-FA titers. Source data for this figure are provided in the Source Data file.

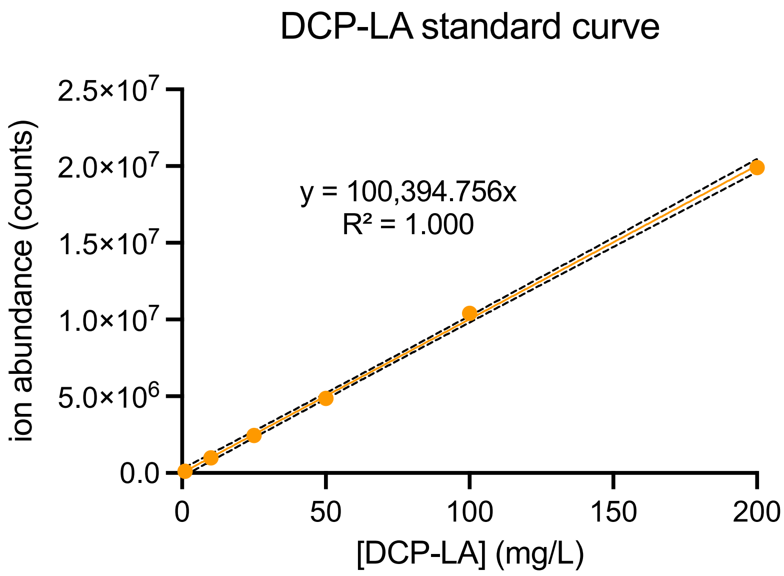

Supplementary Figure 14. DCP-LA standard curve. Varying concentrations of DCP-LA from 10-200 mg/L were measured for the purpose of estimating POP-FA titers. Source data for this figure are provided in the Source Data file.

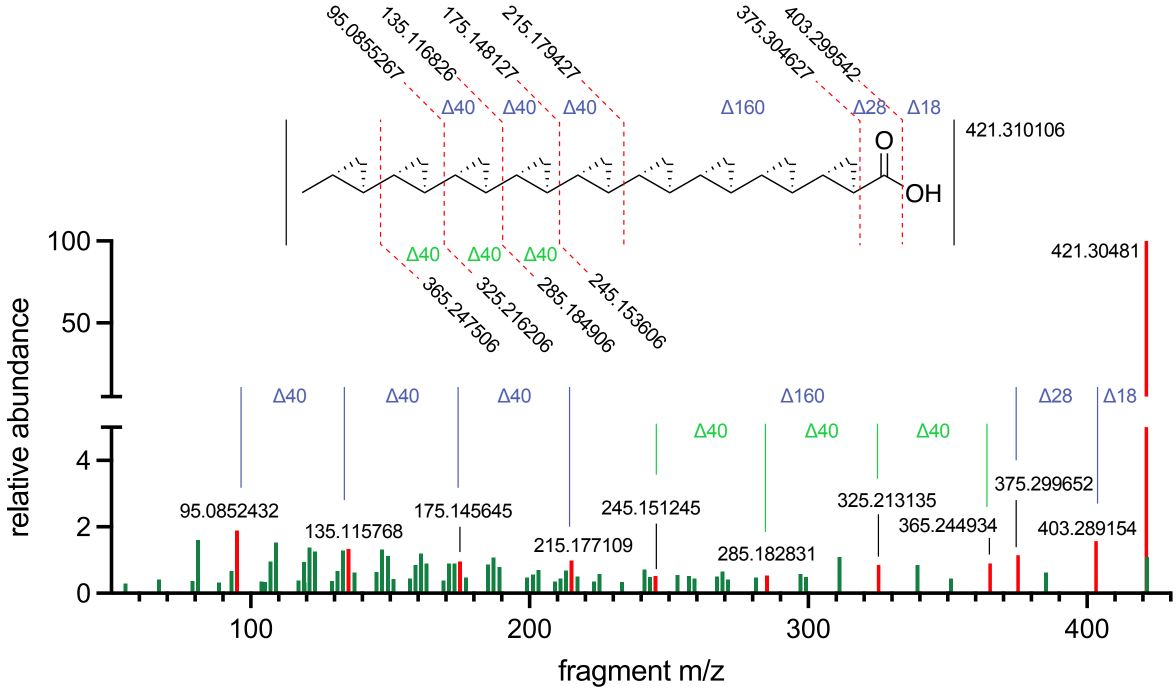

Supplementary Figure 15. Structural prediction of C20:CP9 using LC-MS/MS. The predicted structure of C20:CP9 is shown on top, with mass shifts shown between fragmentation sites. Fragments corresponding to ions detected are indicated with dashed lines. The full spectrum is shown in green with the corresponding ions highlighted in red. Source data for this figure are provided in the Source Data file.

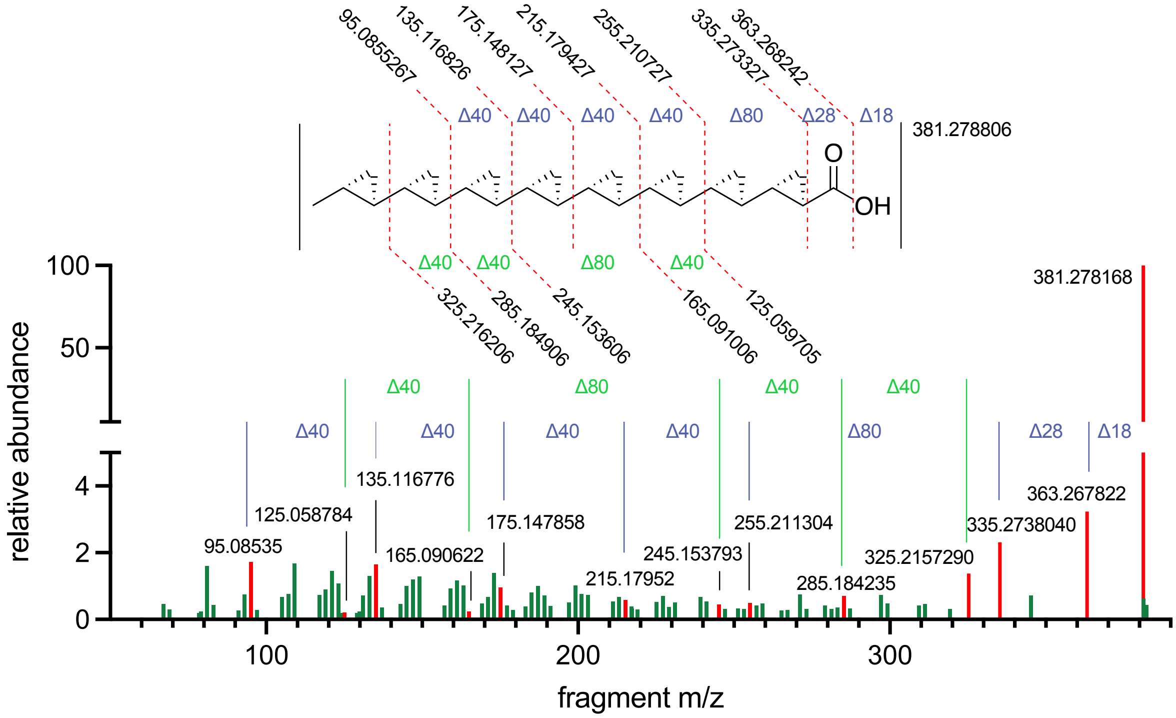

Supplementary Figure 16. Structural prediction of C18:CP8 using LC-MS/MS. The predicted structure of C18:CP8 is shown on top, with mass shifts shown between fragmentation sites. Fragments corresponding to ions detected are indicated with dashed lines. The full spectrum is shown in green with the corresponding ions highlighted in red. Source data for this figure are provided in the Source Data file.

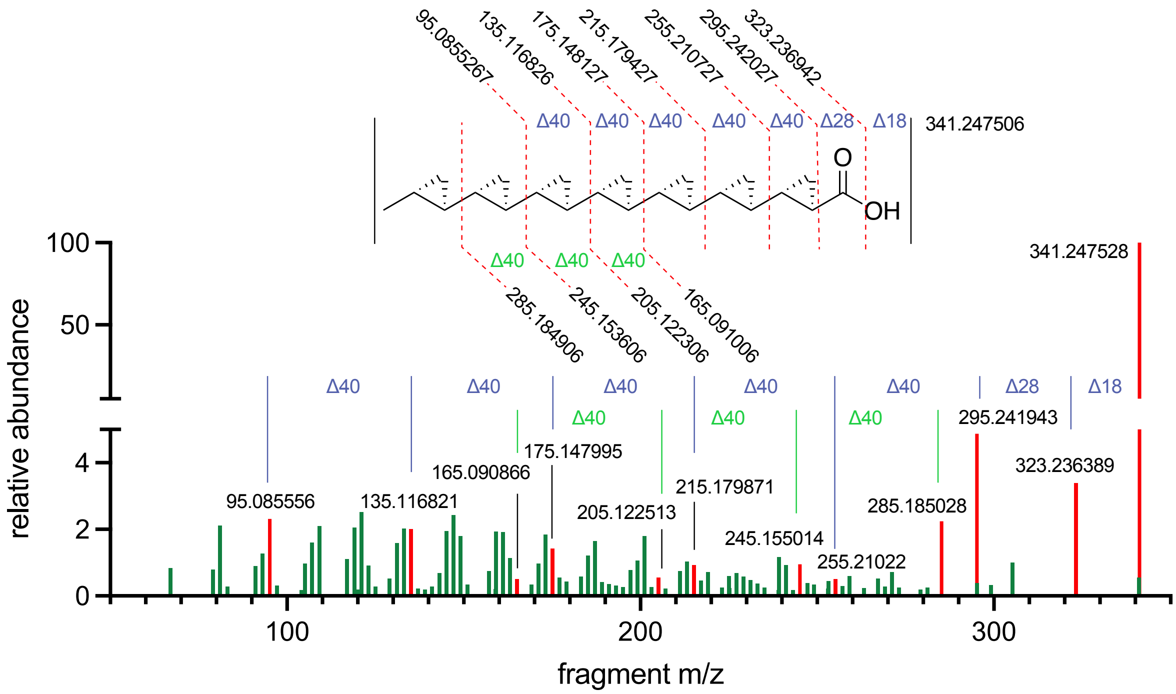

Supplementary Figure 17. Structural prediction of C16:CP7 using LC-MS/MS. The predicted structure of C16:CP7 is shown on top, with mass shifts shown between fragmentation sites. Fragments corresponding to ions detected are indicated with dashed lines. The full spectrum is shown in green with the corresponding ions highlighted in red. Source data for this figure are provided in the Source Data file.

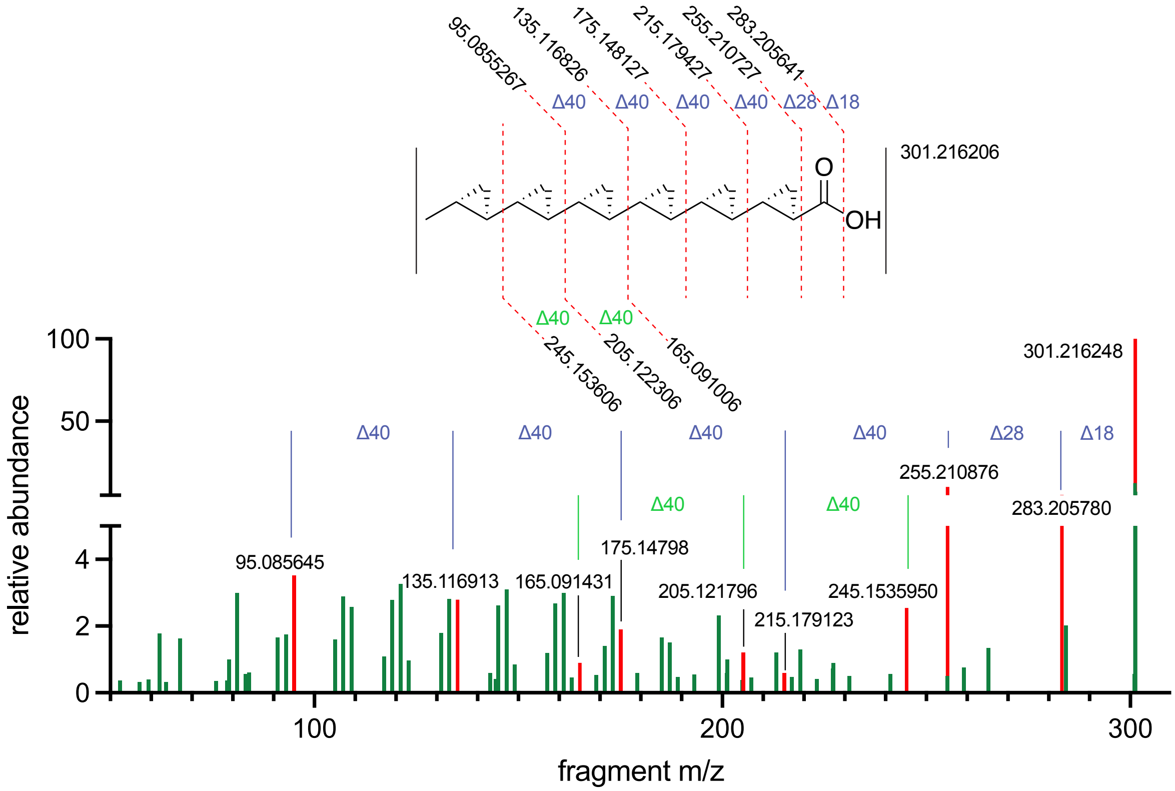

Supplementary Figure 18. Structural prediction of C14:CP6 using LC-MS/MS. The predicted structure of C14:CP6 is shown on top, with mass shifts shown between fragmentation sites. Fragments corresponding to ions detected are indicated with dashed lines. The full spectrum is shown in green with the corresponding ions highlighted in red. Source Data for this figure are provided in the Source Data file.

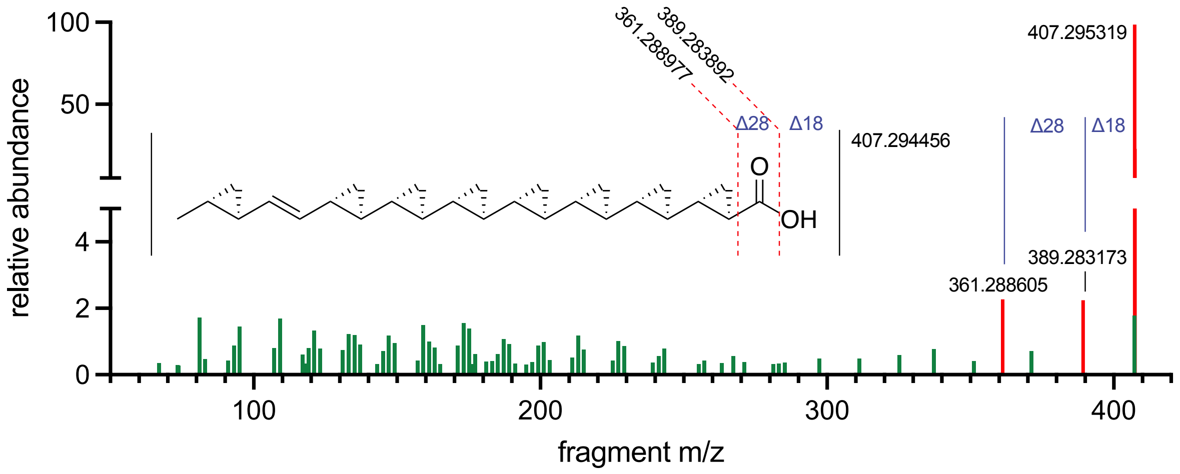

Supplementary Figure 19. Partial structural prediction of C20:CP8 using LC-MS/MS. The partial predicted structure of C20:CP8 is shown on top, with mass shifts show between fragmentation sites. The position of the carbon-carbon double bond is hypothetical and not determined experimentally. Fragments corresponding to ions detected are indicated with dashed lines. The full spectrum is shown in green with the corresponding ions highlighted in red. Source data for this figure are provided in the Source Data file.

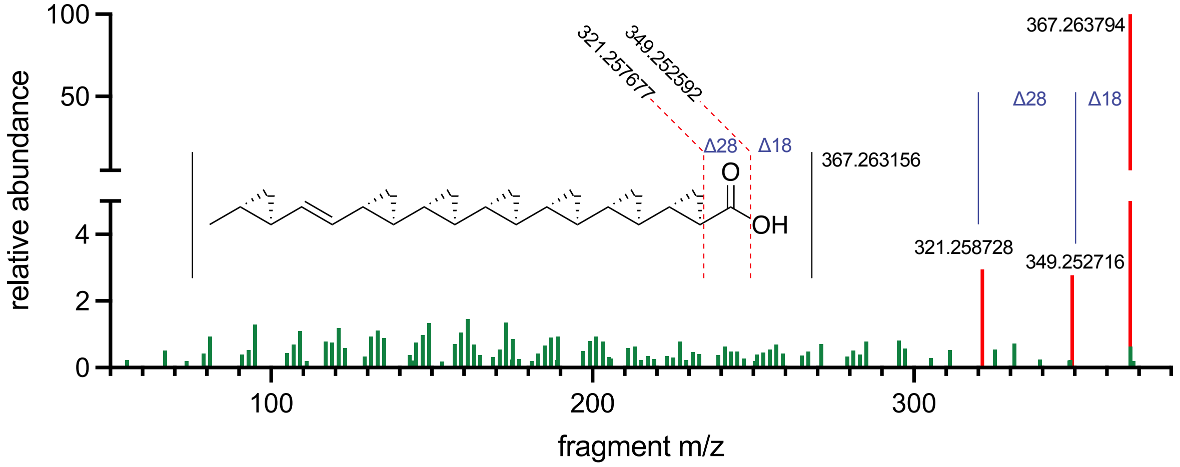

Supplementary Figure 20. Partial structural prediction of C18:CP7 using LC-MS/MS. The partial predicted structure of C18:CP7 is shown on top, with mass shifts show between fragmentation sites. The position of the carbon-carbon double bond is hypothetical and not determined experimentally. Fragments corresponding to ions detected are indicated with dashed lines. The full spectrum is shown in green with the corresponding ions highlighted in red. Source data for this figure are provided in the Source Data file.

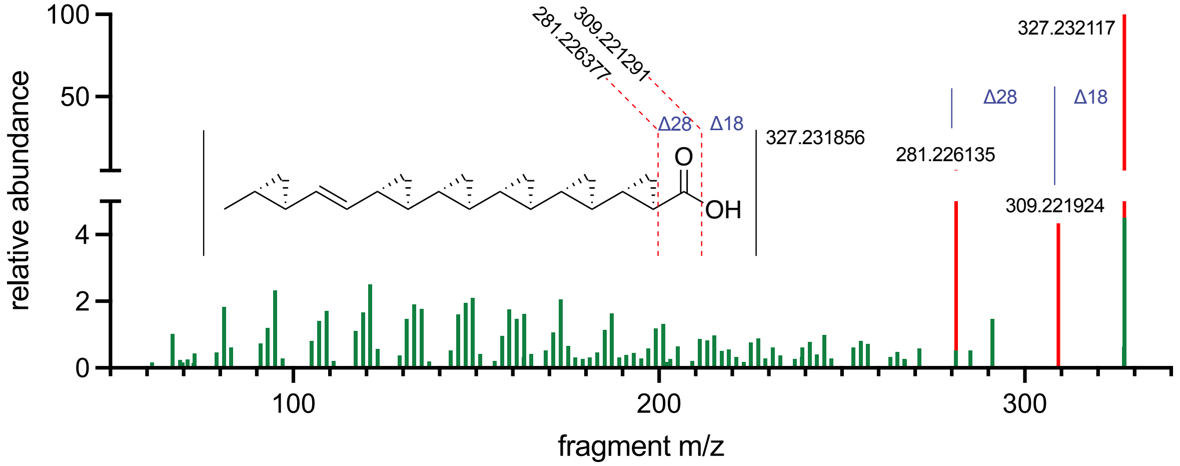

Supplementary Figure 21. Partial structural prediction of C16:CP6 using LC-MS/MS. The partial predicted structure of C16:CP6 is shown on top, with mass shifts show between fragmentation sites. The position of the carbon-carbon double bond is hypothetical and not determined experimentally. Fragments corresponding to ions detected are indicated with dashed lines. The full spectrum is shown in green with the corresponding ions highlighted in red. Source data for this figure are provided in the Source Data file.

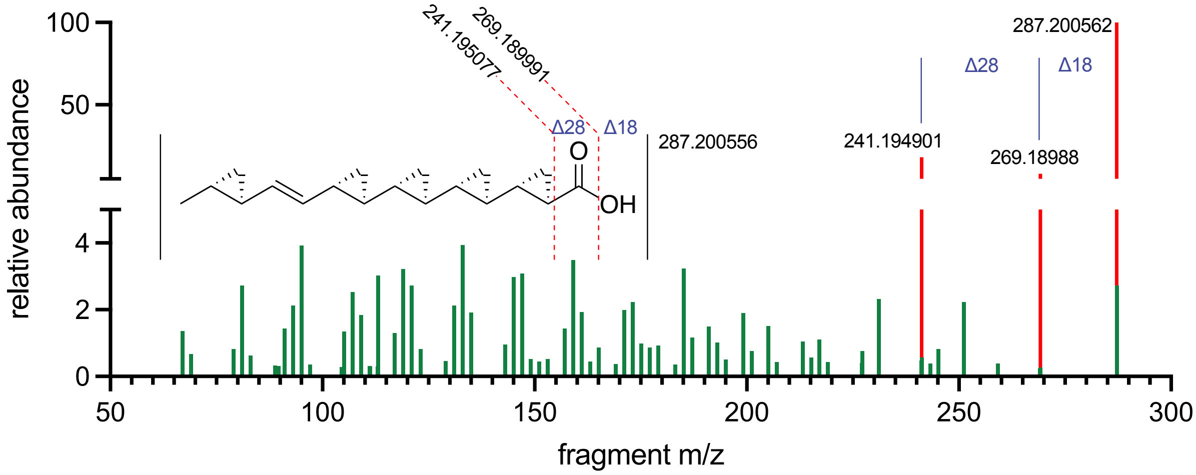

Supplementary Figure 22. Partial structural prediction of C14:CP5 using LC-MS/MS. The partial predicted structure of C14:CP5 is shown on top, with mass shifts show between fragmentation sites. The position of the carbon-carbon double bond is hypothetical and not determined experimentally. Fragments corresponding to ions detected are indicated with dashed lines. The full spectrum is shown in green with the corresponding ions highlighted in red. Source data for this figure are provided in the Source Data file.

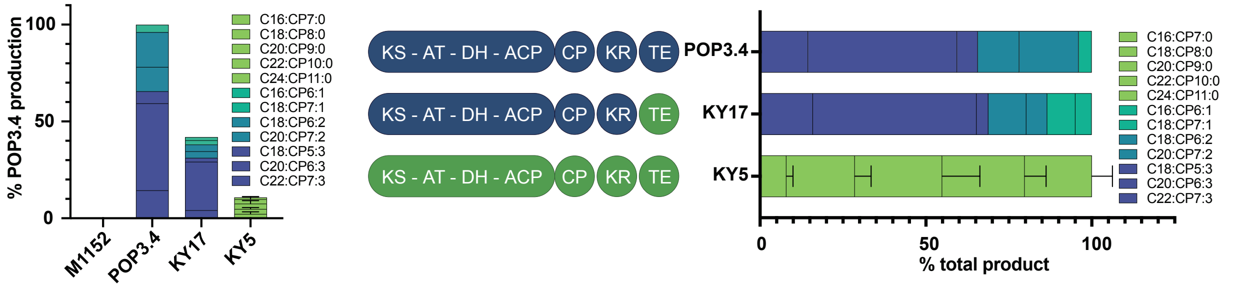

Supplementary Figure 23. Role of POP-TE on pathway product profile. Comparison of product profiles between POP3.4 (*pfl1-pfl4*), KY17 (PPPL; *pfl1-pfl3, log4* TE) and KY5 (LLLL; *log1-log4*) strains. Pfl (navy) and Log (green) domains are shown. Log4 TE does not significantly gatekeep cyclopropanation levels (color coded) of released POP-FAs. All data are presented as mean values; where $n > 2$, error bars indicate the s.d. of biological replicates ($n = 4$ for KY5; $n = 2$ for other groups). All strain information can be found in Supplementary Data 4. Source data for this figure are provided in the Source Data file.

```
log2        ----MLDAGFVHTYIDTHLEQRQVNKIQHGFPSPRYWSRTDVPVEEIAEDRRRVRAAGRD  56
pfl2        MPAIVLTPDFVNNYLDSHLAERQVNKIQHGFPSPRFWNELSVPLDEIGEDRRRLSETHNE  60
                :*  .**:.*:*:** :**************:*.. .**::**.*****:  : .:

log2        SFVNFYVGVPYCIQTDPGKCGYCLFPVEEFQGNAALENYFGYVEREADLYREALSGATLG  116
pfl2        SPVFLYIGVPYCIKTDPGKCGYCLFPVEEFQGNAALENYYGYVEREAEMYREQMEGVLLA  120
            * * :*:******:*************************:*******::*** :.*. *.

log2        AVYFGGGTSNLYREPLYHRLMDLVRGLFPDIAPQADITLEGIPQLFSRAKMQAIKDSGMN  176
pfl2        GAYFGGGTSNLYRPAVYHRIMDMVRRLFPEISDQADLTLEGIPQLFTREKMRAIADSGMN  180
            ..***********  :***:**:** ***:*: ***:*********:* **:** *****

log2        RVSMGIQQVDERLNKLSGRKQTTRHVVQSLEWARELGLAANVDLIFGWPQQTVGTMLKDL  236
pfl2        RISMGVQQINERLNSFSGRKQTTKHVIQSLEWARELGLAANVDLIFGWPQQTVDTLLEDL  240
            *:***:**::****.:*******:**:**************************.*:*:**

log2        QTLVSWNVYDITHYELNVGGPTDFALNRFHELPSTLANLEMYRASRDFLTDQGYEQITAY  296
pfl2        ETLVSWDVYDITHYELNVGGPTDFALNRYHELPSTLANLELYRAGRDFLVDHGYEQLSTY  300
            :*****:*********************:***********:***.****.*:****:::*

log2        NFRKPGDPAGRGYEEGVNRFLDSMDTVGLGYAAVSFFGNSAIGTDRSWSFINHLSLPRYK  356
pfl2        NFRRPGDPTTRDFREGYTTRFDHVDSLGLGYAAITFFGNPALPSGRSWSFINHRSLPQYK  360
            ***:****: *.:.** .  :* :*::******::**** *: :.******** ***:**

                            boundary 1
log2        QALEEGRFPVERGFSHEAADWRLAMLFRSLFGLTVNRADYRAAFGTDVYEEFATVWDGLG  416
pfl2        AAIDNGRFPVERGFRHTPDDWLLMLLFRSLISTDIDRTRYRTALGLDIYEKFATIWDALA  420
             *:::********* *   ** * :*****:.  ::*: **:*:* *:**:***:**.*.

                                          boundary 2
log2        EYGFVEVSDEEVGLVGDGPFYTPMVQALLAEERYRALRERETRAAQARRAARRARRTGTD  476
pfl2        ERGLAKVTPERIKLVDDGAFYAPMISALVAEERYRELREQAARHRRESRGHA--------  472
            * *:.:*: *.: **.** **:**:.**:****** ***: :*  :  *.

log2        GDGAGTEEVVASPGTGAPADTEAPADAEAAAATPARG 513
pfl2        ---AAAGVTLPVPGVGSGG------------------ 488
               *.:  .:  **.*: .
```

Supplementary Figure 24. Alignment of Pfl2 and Log2 and design of chimeric cyclopropanases. L-L-P: N-terminal Log2 and C-terminal Pfl2 chimera with boundary 2; L-P-P: N-terminal Log2 and C-terminal Pfl2 chimera with boundary 1; P-L-L: N-terminal Pfl2 and C-terminal Log2 chimera with boundary 1; P-P-L: N-terminal Pfl2 and C-terminal Log2 chimera with boundary 2.

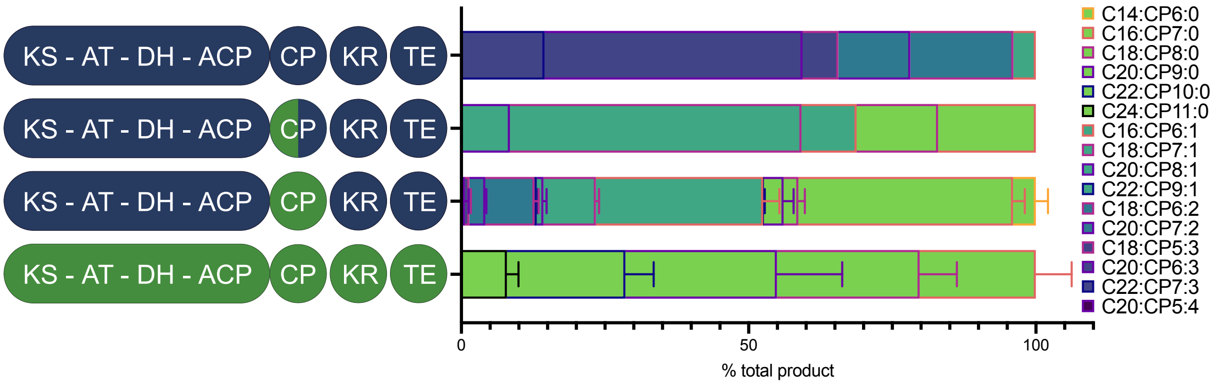

Supplementary Figure 25. POP-FA production profile from KY20 P(L-L-P)PP strain. Strain KY20 expresses *pfl1* iPKS, a chimeric CP containing the *log2* N-terminal subdomain and *pfl2* C-terminal subdomain, *pfl3* KR, and *pfl4* TE. The resulting product profile resembles that of strain KY16 (PLPP). All data are presented as mean values; where *n* > 2, error bars indicate the s.d. of biological replicates (*n* = 2 for POP3.4 and KY20; *n* = 3 for KY16; *n* = 4 for KY5). All strain information can be found in Supplementary Data 4. Source data for this figure are provided in the Source Data file.

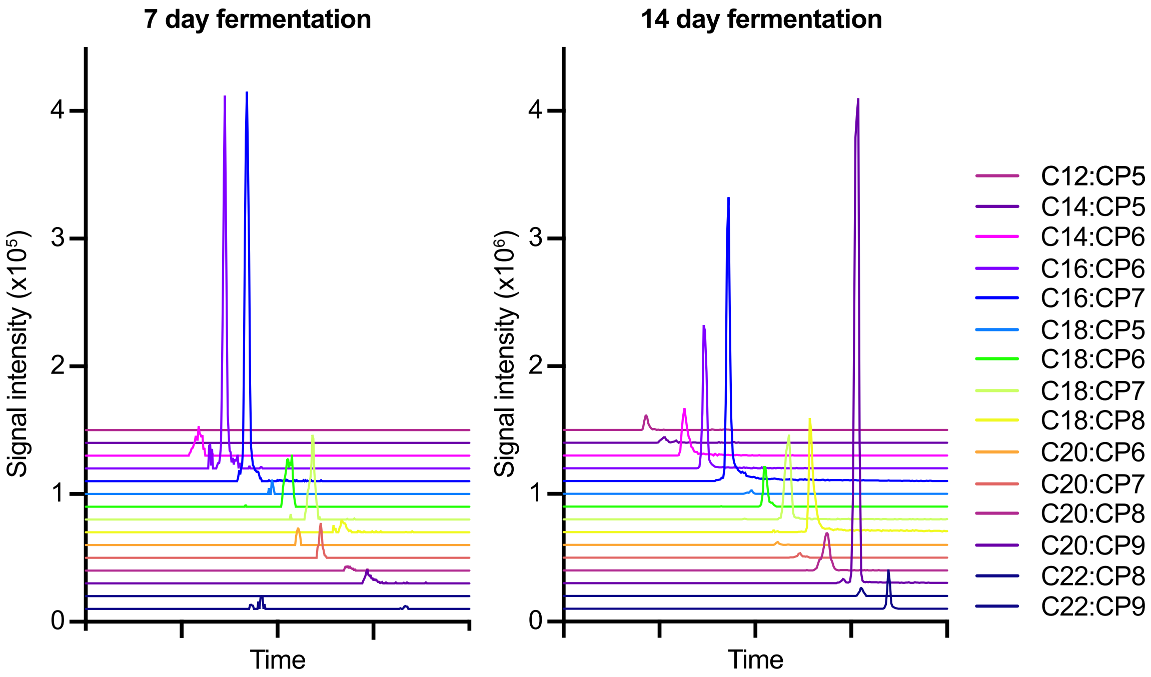

Supplementary Figure 26. Extracted ion chromatograms for PLPP (KY16) products at 7- and 14-day fermentations. Chromatograms are adjusted along the x and y axis for visibility and do not precisely represent retention time or signal intensity. All strain information can be found in Supplementary Data 4. Source data for this figure are provided in the Source Data file.

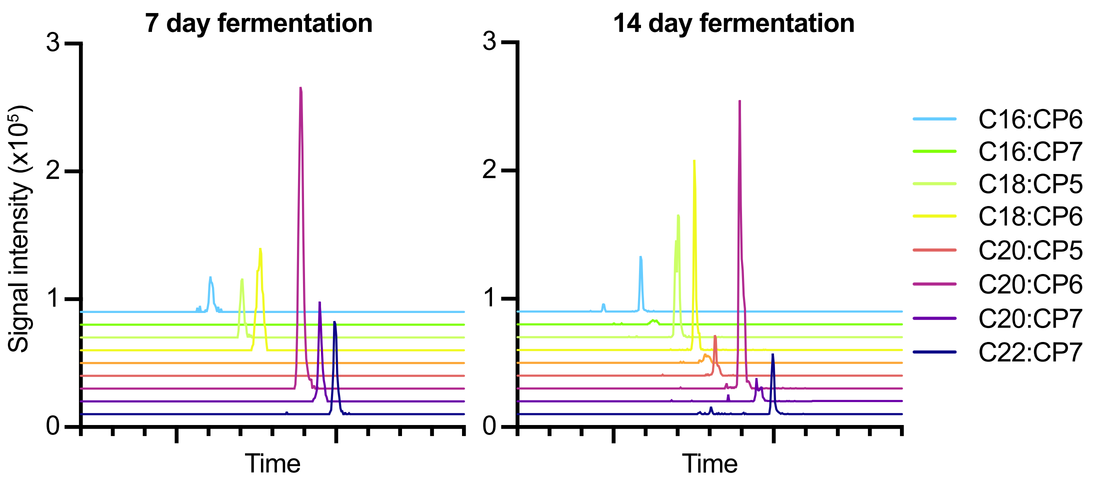

Supplementary Figure 27. Extracted ion chromatograms for PPPP (POP3.4) products at 7- and 14-day fermentations. Chromatograms are adjusted along the x and y axis for visibility and do not precisely represent retention time or signal intensity. All strain information can be found in Supplementary Data 4. Source data for this figure are provided in the Source Data file.

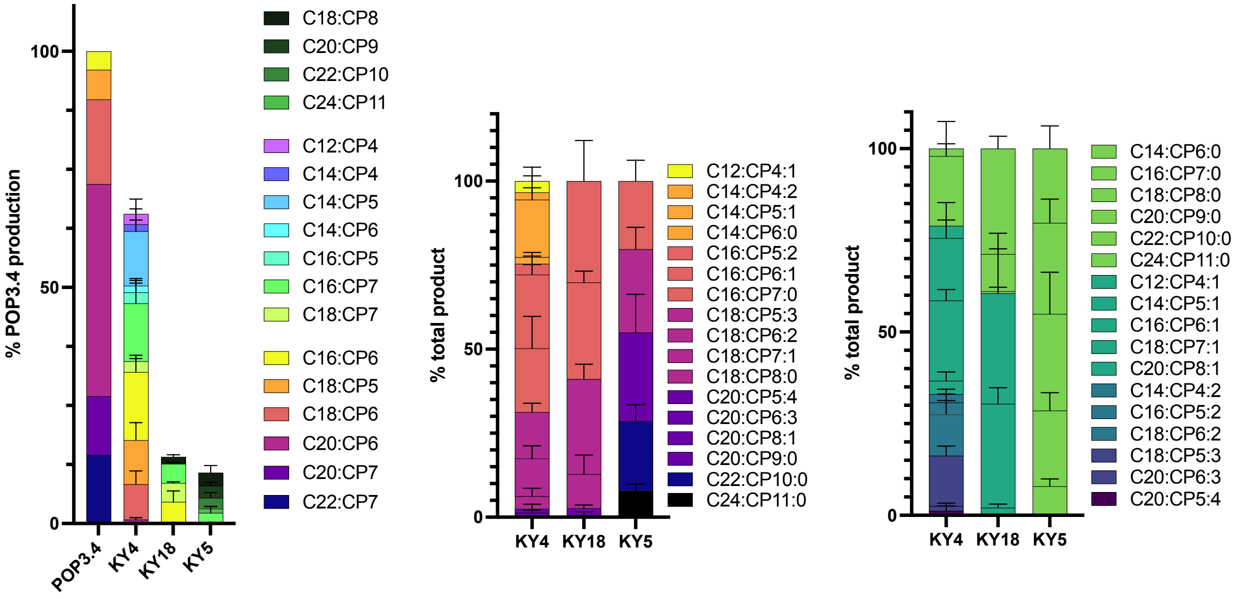

Supplementary Figure 28. POP-FA production titers and profile from JLJP (KY18) strain. Chimeric strain KY18 (JLJP; *jaw4* iPKS, *log2* CP, *jaw6* KR, *pop4* TE) matches the production titers (left) of KY5 (LLLL; *log1-log4*), but approaches the chain length profile (middle) and cyclopropanation profile (right) of KY4 (JJJP; *jaw4-jaw6, pfl4*). All data are presented as mean values; where $n > 2$, error bars indicate the s.d. of biological replicates ($n = 2$ for POP3.4; $n = 3$ for KY18; $n = 4$ for KY5; $n = 5$ for KY4) All strain information can be found in Supplementary Data 4. Source data for this figure are provided in the Source Data file.

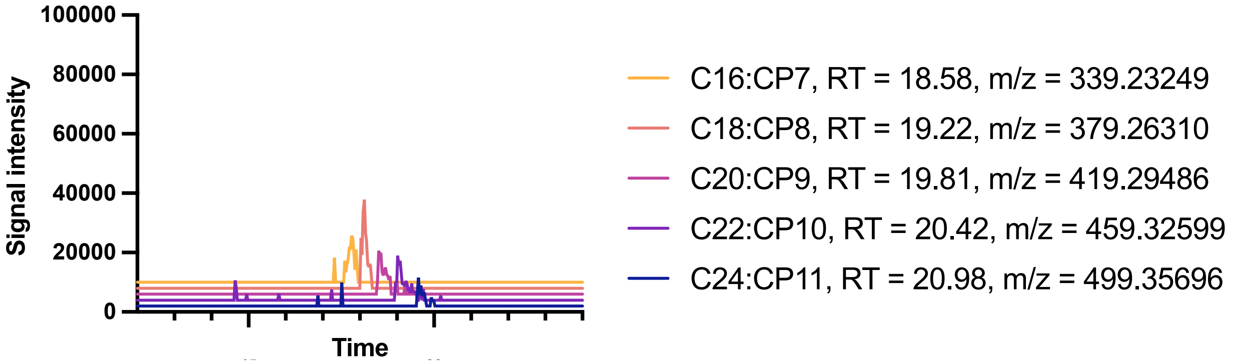

Supplementary Figure 29. Extracted ion chromatograms for LLLL (KY5) products. Chromatograms are adjusted along the x and y axis for visibility and do not precisely represent retention time or signal intensity. All strain information can be found in Supplementary Data 4. Source data for this figure are provided in the Source Data file.

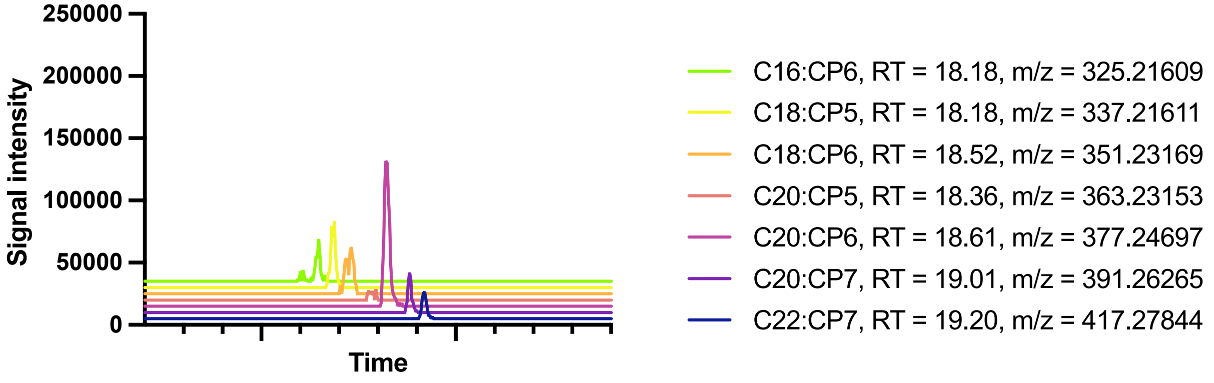

Supplementary Figure 30. Extracted ion chromatograms for PPJP (KY9) products. Chromatograms are adjusted along the x and y axis for visibility and do not precisely represent retention time or signal intensity. All strain information can be found in Supplementary Data 4. Source data for this figure are provided in the Source Data file.

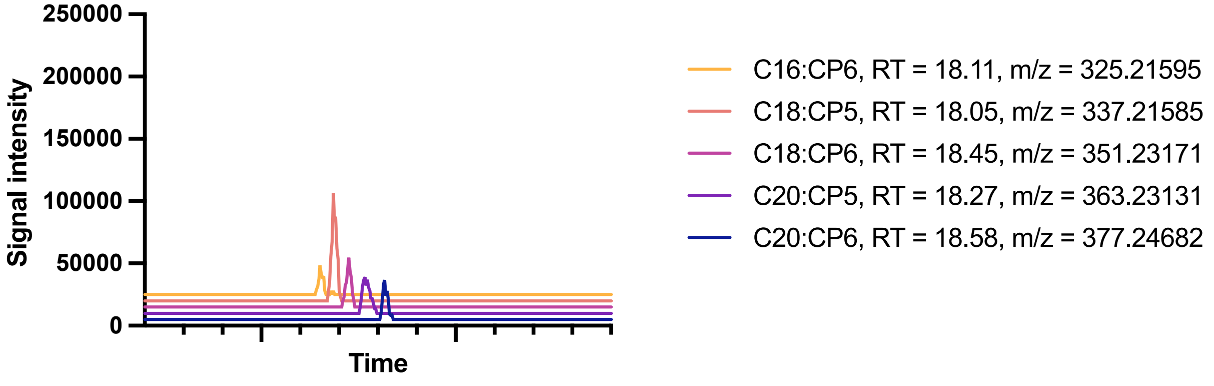

Supplementary Figure 31. Extracted ion chromatograms for JPPP (KY10) products. Chromatograms are adjusted along the x and y axis for visibility and do not precisely represent retention time or signal intensity. All strain information can be found in Supplementary Data 4. Source data for this figure are provided in the Source Data file.

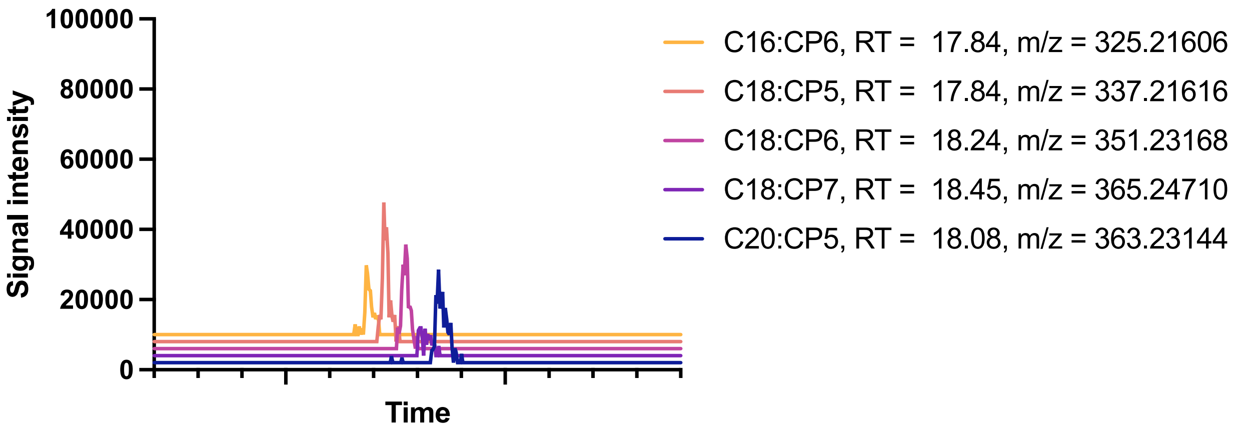

Supplementary Figure 32. Extracted ion chromatograms for JPJP (KY11) products. Chromatograms are adjusted along the x and y axis for visibility and do not precisely represent retention time or signal intensity. All strain information can be found in Supplementary Data 4. Source data for this figure are provided in the Source Data file.

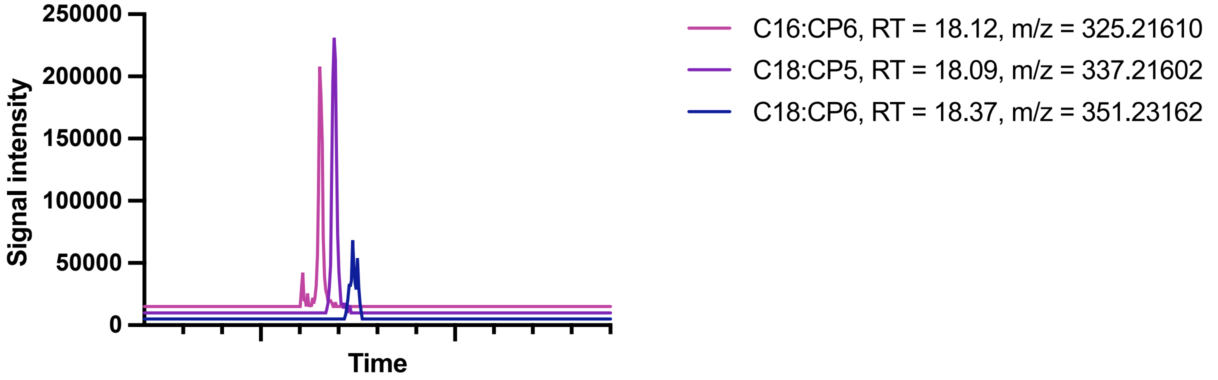

Supplementary Figure 33. Extracted ion chromatograms for PJPP (KY12) products. Chromatograms are adjusted along the x and y axis for visibility and do not precisely represent retention time or signal intensity. All strain information can be found in Supplementary Data 4. Source data for this figure are provided in the Source Data file.

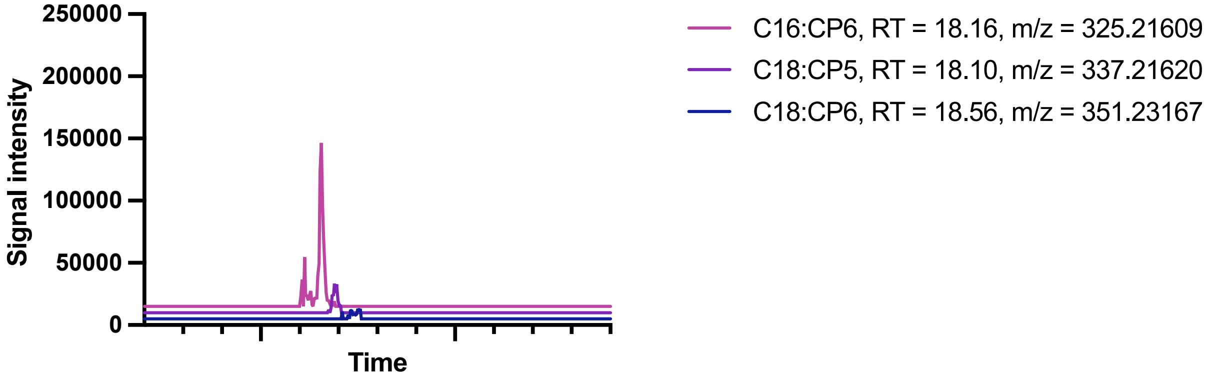

Supplementary Figure 34. Extracted ion chromatograms for PJJP (KY13) products. Chromatograms are adjusted along the x and y axis for visibility and do not precisely represent retention time or signal intensity. All strain information can be found in Supplementary Data 4. Source data for this figure are provided in the Source Data file.

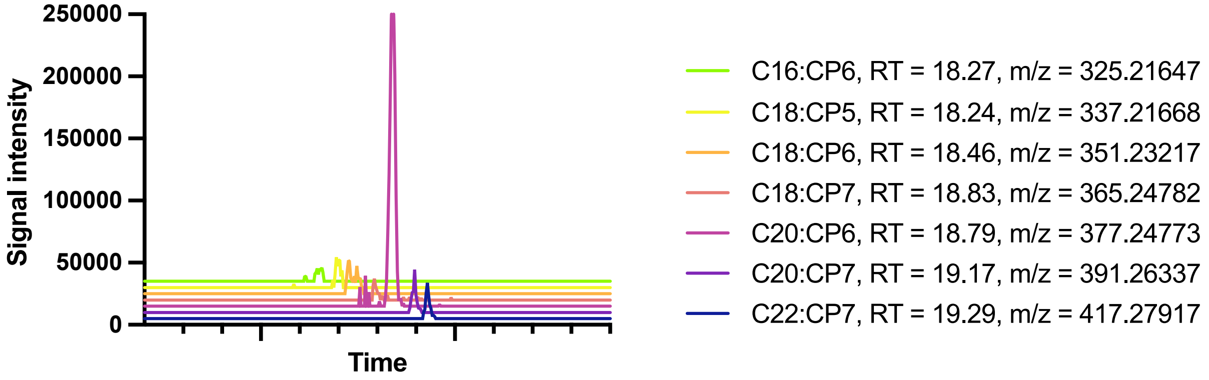

Supplementary Figure 35. Extracted ion chromatograms for PPPL (KY17) products. Chromatograms are adjusted along the x and y axis for visibility and do not precisely represent retention time or signal intensity. All strain information can be found in Supplementary Data 4. Source data for this figure are provided in the Source Data file.

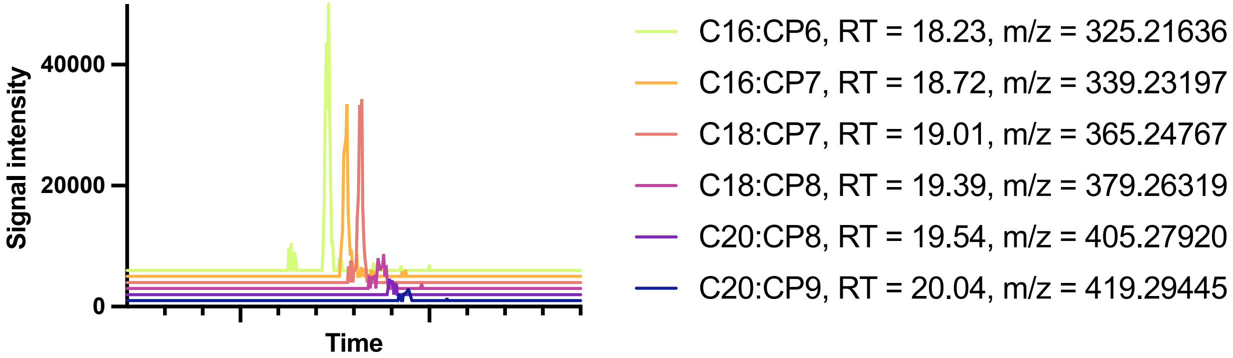

Supplementary Figure 36. Extracted ion chromatograms for JLJP (KY18) products. Chromatograms are adjusted along the x and y axis for visibility and do not precisely represent retention time or signal intensity. All strain information can be found in Supplementary Data 4. Source data for this figure are provided in the Source Data file.

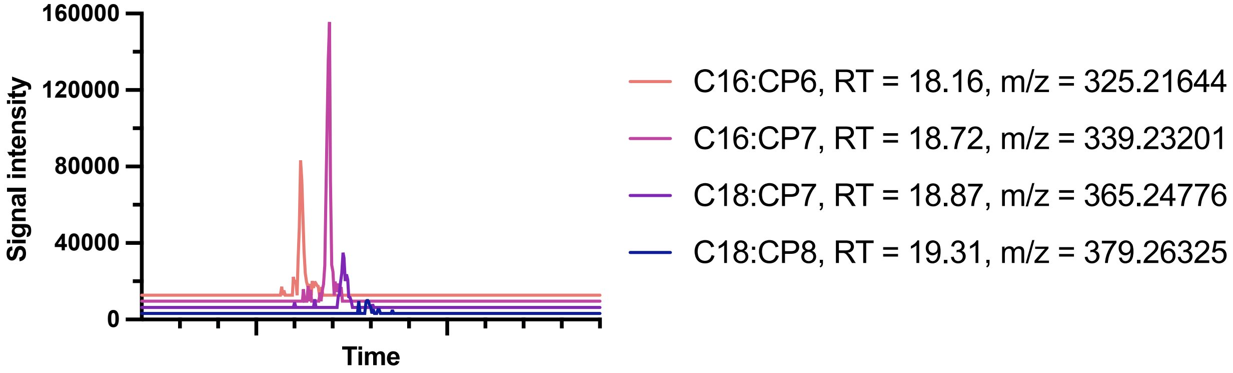

Supplementary Figure 37. Extracted ion chromatograms for P(L-L-P)PP (KY20) products. Chromatograms are adjusted along the x and y axis for visibility and do not precisely represent retention time or signal intensity. All strain information can be found in Supplementary Data 4. Source data for this figure are provided in the Source Data file.

Supplementary Table 1. Estimation of POP-FA titers using reference molecules.

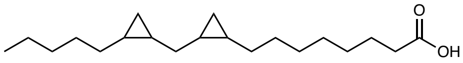

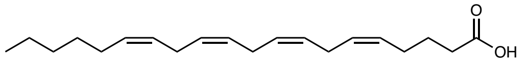

Compound	Peak area	Concentration (mg/L)	Estimated 100x sample concentration (mg/L)	Estimated titer (µg/L)
			POP3.4	POP3.4
DCPLA	4863824	50	-	-
ARA	4984015	50	-	-
Sum of POP peak areas	5889275	-	59.0816 – 60.5416	590.816 – 605.416

Compound	Peak area	concentration	**Estimated 100x sample concentration (mg/L)**	**Estimated titer (µg/L)**
			**POP3.4**	**POP3.4**
DCPLA	4863824	50 mg / L	-	-
ARA	4984015	50 mg / L	-	-
Sum of POP peak areas	5889275	-	59.0816 - 60.5416	**590.816 - 605.416**

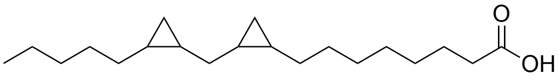

DCPLA

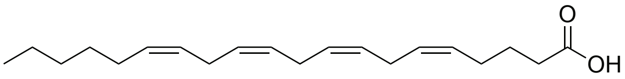

ARA

Supplementary Table 2. Evaluation of the performance of the initial freezing point model against measured freezing points

Molecular structure	Name/Notes	Literature freezing point, ºC	Initial model freezing point, ºC	Final freezing point model, ºC
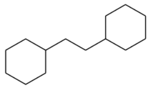		11.5	11.7	
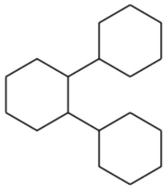	1,1’:2’,1’’-Tercyclohexane	18.85	52.08	5.5
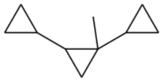	syntin	-73	7.33	-51.91

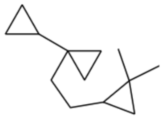	Cyclopropanated myrcene	<-70	29.5	-40.47

Supplementary References

1. Herbst, D.A., Jakob, R.P., Zähringer, F., and Maier, T. (2016). Mycocerosic acid synthase exemplifies the architecture of reducing polyketide synthases. Nature *531*, 533–537.
2. Wang, W., Li, S., Li, Z., Zhang, J., Fan, K., Tan, G., Ai, G., Lam, S.M., Shui, G., Yang, Z., et al. (2019). Harnessing the intracellular triacylglycerols for titer improvement of polyketides in Streptomyces. Nat. Biotechnol. *38*, 76–83.
3. Gollis, M. H., Belenyessy, L. I., Gudzinowics, B. J., Koch, S. D., Smith, J. O., Wineman, R. J. (1962). Evaluation of pure hydrocarbons as jet fuels, J. Chem. Eng. Data *7*, 311-316.
4. Xie, J., Zhang, X., Pan, L., Nie, G., E, X., Liu, Q., Wang, P., Li, Y., Zou, J-J. (2017). Renewable high-density spiro-fuels from lignocellulose-derived cyclic ketone, Chem. Commun. *53*, 10303. 10.1039/c7cc05101h.
5. Li., Z., Pan, L., Nie, G., Xie, J., Xie, J., Zhang, X., Wang, L., Zou, J-J. (2018). Synthesis of high-performance jet fuel blends from biomass-derived 4-ethylphenol and phenylmethanol, Chem. Eng. Sci. *191*, 343-349. 10.1016/j.ces.2018.07.001.
6. Zhang, X., Pan, L., Wang, L., Zou, J-J. (2018) Review on synthesis and properties of high-energy-density liquid fuels: Hydrocarbons, nanofluids and energetic ionic liquids, Chem. Eng. Sci. *180*, 95-125. 10.1016/j.ces.2017.11.044.
7. Zarezin, D. P., Rudakova, M. A., Shorunov, S. V., Sultanova, M. U., Samoilov, V. O., Maximov, A. L., Bermeshev, M. V. (2022) Design and preparation of liquid polycyclic norboranes as potential high performance fuels for aerospace propulsion, Fuel Process. Technol. *225*, 107056. 10.1016/j.fuproc.2021.107056.
8. Wang, W., Liu, Y., Shi, C., Pan, L., Zhang, X., Zou, J-J. (2022) High energy density renewable fuels based on multicyclic sesquiterpene: Synthesis and performance, Fuel *318*, 123665. 10.1016/j.fuel.2022.123665
9. Muldoon, J. A., Harvey, B. G. (2020) Bio-Based cycloalkanes: The missing link to high-performance sustainable jet fuels, ChemSusChem *13*, 5777-5807. 10.1002/cssc.202001641.
10. GOX/Sintin http://www.astronautix.com/g/goxsintin.html.

11. Cruz-Morales, P., Yin, K., Landera, A., Cort, J.R., Young, R.P., Kyle, J.E., Bertrand, R., Iavarone, A.T., Acharya, S., Cowan, A., et al. (2022). Biosynthesis of polycyclopropanated high energy biofuels. Joule *6*, 1590–1605.
12. Barrett, A.G.M., Kasdorf, K., Tustin, G.J., and Williams, D.J. (1995). Determination of the full structure and absolute stereochemistry of the antifungal agent FR-900848: an X-ray crystallographic study of (1 R ,3 S ,4 R ,6 S ,7 S ,9 R ,10 S ,12 R)-quatercyclopropyl-1,12-dimethanediyl di-4-bromobenzoate. Journal of the Chemical Society, Chemical Communications *0*, 1143–1144.
13. Yoshida, M., Ezaki, M., Hashimoto, M., Yamashita, M., Shigematsu, N., Okuhara, M., Kohsaka, M., and Horikoshi, K. (1990). A novel antifungal antibiotic, FR-900848. I. Production, isolation, physico-chemical and biological properties. J. Antibiot. *43*, 748–754.
14. Hiratsuka, T., Suzuki, H., Minami, A., and Oikawa, H. (2017). Stepwise cyclopropanation on the polycyclopropanated polyketide formation in jawsamycin biosynthesis. Org. Biomol. Chem. *15*, 1076–1079.
