# Supplementary material for: Polyketide synthase-based controlled synthesis of polycyclopropanated fuel molecules
Source: Nat Commun. 2026 May 27;17:6904. doi: 10.1038/s41467-026-73172-3 (PMC13389473; doi:10.1038/s41467-026-73172-3)
Supplement: Supplementary file 2 — Description of Additional Supplementary Files [file 41467_2026_73172_MOESM2_ESM.pdf]

Description of Additional Supplementary Files

Supplementary Data 1: Structures, melting points, and references for the additional molecules used in the re-training of the melting point ML model

Supplementary Data 2: Calculated fuel properties for postulated and experimentally produced POP-derived molecules

Supplementary Data 3: Proposed POP-FA natural diversity and classifications. Class I POP-FAs produced by POP3.4 (PPPP) strain contain no cyclopropanation skips.[11] Class II POP-FAs produced by KY4 (JJJP) strain may contain the same distinctive cyclopropanation skip in the second ketide unit as characterized in jawsamycin.[12,13,14] Class III POP-FAs predominantly produced by KY5 (LLLL) and KY16 (PLPP) strains, with minor production from KY4 (JJJP), are fully cyclopropane-saturated. Unsaturated products from KY16 (PLPP) have unknown carbon-carbon double bond positions and may be class I, class II, and/or an undescribed class. All strain information can be found in Supplementary Data 4.

Supplementary Data 4: Strains and plasmids used in this study

Supplementary Data 5: Primers used in this study

Supplementary Data 6: Genes and promoters used in this study

Supplementary Data 7: LC-MS/MS instrumentation and parameters

Supplementary Data 8: Source data for supplementary LC-MS extracted ion chromatograms
