# Supplementary material for: Polyketide synthase-based controlled synthesis of polycyclopropanated fuel molecules
Source: Nat Commun. 2026 May 27;17:6904. doi: 10.1038/s41467-026-73172-3 (PMC13389473; doi:10.1038/s41467-026-73172-3)
Supplement: Supplementary file 11 — Reporting Summary [file 41467_2026_73172_MOESM11_ESM.pdf]

nature portfolio

Corresponding author(s): Qingyun Dan, Jay D. Keasling

Last updated by author(s): Mar 7, 2026

Reporting Summary

Nature Portfolio wishes to improve the reproducibility of the work that we publish. This form provides structure for consistency and transparency in reporting. For further information on Nature Portfolio policies, see our Editorial Policies and the Editorial Policy Checklist.

Statistics

For all statistical analyses, confirm that the following items are present in the figure legend, table legend, main text, or Methods section.

n/a	Confirmed	
☐	☒	The exact sample size (*n*) for each experimental group/condition, given as a discrete number and unit of measurement
☐	☒	A statement on whether measurements were taken from distinct samples or whether the same sample was measured repeatedly
☒	☐	The statistical test(s) used AND whether they are one- or two-sided<br>*Only common tests should be described solely by name; describe more complex techniques in the Methods section.*
☒	☐	A description of all covariates tested
☒	☐	A description of any assumptions or corrections, such as tests of normality and adjustment for multiple comparisons
☐	☒	A full description of the statistical parameters including central tendency (e.g. means) or other basic estimates (e.g. regression coefficient) AND variation (e.g. standard deviation) or associated estimates of uncertainty (e.g. confidence intervals)
☒	☐	For null hypothesis testing, the test statistic (e.g. *F*, *t*, *r*) with confidence intervals, effect sizes, degrees of freedom and *P* value noted<br>*Give P values as exact values whenever suitable.*
☒	☐	For Bayesian analysis, information on the choice of priors and Markov chain Monte Carlo settings
☒	☐	For hierarchical and complex designs, identification of the appropriate level for tests and full reporting of outcomes
☒	☐	Estimates of effect sizes (e.g. Cohen's *d*, Pearson's *r*), indicating how they were calculated

Our web collection on statistics for biologists contains articles on many of the points above.

Software and code

Policy information about availability of computer code

Data collection	XCalibur 2.0.6
Data analysis	XCalibur 2.0.6, DIA-NN, AlphaFold 3, CLC Genomics Workbench, SignalMap, PyMOL 2.5.2, CORASON, MUSCLE, Jalview, IQ-tree

For manuscripts utilizing custom algorithms or software that are central to the research but not yet described in published literature, software must be made available to editors and reviewers. We strongly encourage code deposition in a community repository (e.g. GitHub). See the Nature Portfolio guidelines for submitting code & software for further information.

Data

Policy information about availability of data

All manuscripts must include a data availability statement. This statement should provide the following information, where applicable:

- Accession codes, unique identifiers, or web links for publicly available datasets
- A description of any restrictions on data availability
- For clinical datasets or third party data, please ensure that the statement adheres to our policy

The DNA sequences of the fuelimycin BGC from Streptomyces albireticuli NRRL-B1670 are available at MiBig: BGC0002138 with Protein IDs PAU45552.1 (pfl1), PAU45553.1 (pfl2), PAU45554.1 (pfl3), and PAU45555.1 (pfl4).
The DNA sequences of plasmids and strains used in this study have been deposited in the public version of JBEI registry (https://public-registry.jbei.org/). Strains and plasmids used in this study, and listed with JPUB_IDs in Supplementary Data 4, are available upon request through the Joint BioEnergy Institute Strain Registry

(https://public-registry.jbei.org/folders/946).

The RNAseq data generated in this study have been deposited in the Genome Sequence Archive in National Genomics Data Center, China National Center for Bioinformation / Beijing Institute of Genomics, Chinese Academy of Sciences, under the GSA accession code CRA039551 [https://ngdc.cncb.ac.cn/gsa/browse/CRA039551].

The protein mass spectrometry data have been deposited to the ProteomeXchange Consortium via the PRIDE partner repository with the dataset identifier PXD057441 [https://www.ebi.ac.uk/pride/archive/projects/PXD057441].

The processed LC-MS data generated in this study are provided in the Source Data file and Supplementary Data 8.

Research involving human participants, their data, or biological material

Policy information about studies with human participants or human data. See also policy information about sex, gender (identity/presentation), and sexual orientation and race, ethnicity and racism.

Reporting on sex and gender	n/a
Reporting on race, ethnicity, or other socially relevant groupings	n/a
Population characteristics	n/a
Recruitment	n/a
Ethics oversight	n/a

Note that full information on the approval of the study protocol must also be provided in the manuscript.

Field-specific reporting

Please select the one below that is the best fit for your research. If you are not sure, read the appropriate sections before making your selection.

☒ Life sciences ☐ Behavioural & social sciences ☐ Ecological, evolutionary & environmental sciences

For a reference copy of the document with all sections, see nature.com/documents/nr-reporting-summary-flat.pdf

Life sciences study design

All studies must disclose on these points even when the disclosure is negative.

Sample size	No statistical methods were used to pre-determine sample sizes but our sample sizes are similar to those reported in previous publications. Transcriptomics, proteomics analysis and cultivation experiments were conducted in at least triplicate, unless specifically mentioned otherwise in the manuscript.
Data exclusions	The quantity of the targeted proteins in the cell culture samples was plotted using the average mass spectrometry signal response of the three most intense tryptic peptides of each identified protein (Top3 method).
Replication	All experiments were replicated at least two times unless specifically stated otherwise in the manuscript, e.g., transcriptomics and bioreactor experiments. All replication attempts were successful.
Randomization	Data points were not randomized from the analyses.
Blinding	Blinding is not applicable to this study because the experimental design and data analysis involve objective biological measurements or computational analyses that are not subject to observer bias.

Reporting for specific materials, systems and methods

We require information from authors about some types of materials, experimental systems and methods used in many studies. Here, indicate whether each material, system or method listed is relevant to your study. If you are not sure if a list item applies to your research, read the appropriate section before selecting a response.

Materials & experimental systems

n/a	Involved in the study
☒	☐ Antibodies
☒	☐ Eukaryotic cell lines
☒	☐ Palaeontology and archaeology
☒	☐ Animals and other organisms
☒	☐ Clinical data
☒	☐ Dual use research of concern
☒	☐ Plants

Methods

n/a	Involved in the study
☒	☐ ChIP-seq
☒	☐ Flow cytometry
☒	☐ MRI-based neuroimaging

Plants

Seed stocks	n/a
Novel plant genotypes	n/a
Authentication	n/a
