# Supplementary material for: Polyketide synthase-based controlled synthesis of polycyclopropanated fuel molecules
Source: Nat Commun. 2026 May 27;17:6904. doi: 10.1038/s41467-026-73172-3 (PMC13389473; doi:10.1038/s41467-026-73172-3)
Supplement: Supplementary file 12 — Transparent Peer Review file [file 41467_2026_73172_MOESM12_ESM.pdf]

nature portfolio

Peer Review File

Polyketide synthase-based controlled synthesis of polycyclopropanated fuel molecules

Corresponding Author: Professor Jay Keasling

This file contains all reviewer reports in order by version, followed by all author rebuttals in order by version.

Version 0:

Reviewer comments:

Reviewer #1

(Remarks to the Author)
In developing new jet fuel, authors have been conducting research based on the advantages of microbial polycyclopropanated fatty acids (POP-FA). In 2022, they succeeded in genome mining of a known jawsamycin-like BGC and found a promising research target named fuelimycin. Following this finding, in this study, they initially developed a prediction program to identify suitable POP-FA, that hardly solidify at low temperatures and has high energy density, is C12:CP5 or C14:CP6, and set the goal of producing them as the research goal.
In addition to BGCs for the two POP-FAs, fuelimycin (class I) and jawsamycin (class II), authors added the third BGC from S. longispororuber, which produces a new POP-FA (Class III) in which all unsaturated bonds of the parent polyunsaturated fatty acid are cyclopropanated. Using this three-type iPKS system, they tried to elucidate the complex regulatory mechanism of POP-FA formation. Since the iPKS system involved in the production of POP-FA has independent enzymatic functions for CP, KR, and TE, they succeeded in preparing strains that produce the desired POP-FAs by repeatedly swapping each domain of the three BGCs.
Although the mechanism by which POP-FA skeletal construction is carried out has not yet been fully elucidated, important academic discoveries have been made in the course of this research that give rise to expectations for further developments in the future, including that the iPKS system that gives rise to the POP structure is not an equimolar complex, but that the reaction proceeds with a high iPKS/CP ratio, and that the two domains, stand-alone iPKS/CP, play a central role in controlling the chain length and number of cyclopropanation.
The paper is clearly written, properly cited, and the data is of high quality. I believe this manuscript provides sufficient novelty and rigor to warrant publication in Nature Communications and would have impact in the natural products field and garner general interest from synthetic biologists. I have a few suggestions and comments I would ask the authors to address. Please find my suggestions below.

Comments
1) In this paper, unlike ref. 24, all calculations of titers and structure determinations were based on LC-MS data. All discussions of swapping experiments use this data, so referring to ref. 24 is insufficient. It is necessary to clarify whether the experiment was performed using crude extracts or after enriching POP-FA fractions. In addition, the pretreatment procedures for the samples to be analyzed should also be described.
2) Regarding the structure determination, the data presented in the SI (Figures S16-S24) can be considered to be inferred only from the ion chromatogram and HR-MS data for M+. It cannot be denied that the CP skip may really occur on a site other than the carboxyl group, and I believe that the structure of the new compounds needs to be determined by LC-MS/MS analysis as shown in the SI of ref. 24. Furthermore, utilizing UV data may help to determine the terminal conjugated system by the absorption wavelength, which may enable more accurate identification of the position of the double bonds.
3) Regarding the titer, Supplemental Table 1 lists three types of internal standard substances. Without detailed explanation, it is difficult to understand how to use the three types of standard substances. In what concentration range can reliable data be obtained? In particular, the peaks of trace components are split in a complex manner, and it would be necessary to show how they are processed to calculate accurate peak areas. In ref. 24, a standard POP-FAs fuelimycins were isolated and characterized, using these samples, it seems possible to verify the validity of the use of the three standard substances.

Minor points
P13, Figure 5 caption; (pop1, log2, pop3, pop4; PLPP). Popshould be Pfl.

Reviewer #2

(Remarks to the Author)
In this manuscript, Yin and coworkers investigate the determinants of chain length, degree of cylclopropanation, and saturation of polycyclopropanated (POP) fuel molecules produced in an engineered Streptomyces strain by an iterative polyketide synthase (iPKS). This work is a nice follow-up of this group's discovery of the encoding biosynthetic gene clusters a few years ago. They start by computationally predicting fuel energy density and freezing point of modified POPs to guide modifications to the pathway to improve these properties, identifying shorter chains and a higher degree of cylclopropanation as targets. Based on this, they combinatorially profile pathway variants with different iPKSs, cyclopropanases, and thioesterases to demonstrated production of cyclopropane-saturated molecules. Overall, the work is comprehensive, well executed, the results clearly presented, and the narrative logical. The authors are applauded for a high-quality, rigorous study. My primary concern is the impact of the work, as described below.

The authors indicate (correctly) that the identified molecules are some of the most energy-dense molecules produced in a biological system. However, the titer is so low that they cannot even be structurally characterized (estimated 42-94 µg/L, Table S1). And, they are always produced as a mixture of several different chain lengths. This is despite quite a bit of effort on the part of the authors to improve titer, for example by exploring different hosts, refactoring the gene cluster, supplementing PQQ, and extending the fermentation time. Given this, it is hard to imagine that these could be successfully scaled to produce aviation fuel molecules economically.

As such, while the biosynthetic aspects of the study are highly interesting, the broader impact this work will have on sustainable energy is questionable. The authors acknowledge this in a couple of sentences in the Discussion, but I think that: 1) the titers achieved should be more openly reported in the main manuscript so the reader can more easily get a sense of the current state of the technology, and 2) a more frank and extensive discussion about whether it will even be possible to achieve the g/L scales needed for this to be commercially viable should also be included.

Reviewer #3

(Remarks to the Author)
This manuscript addresses the important issue of generating efficient bio-derived fuels with high energy content. The authors' approach to solving this problem is clearly innovative and warrants publication. However, there are several deficiencies that must be addressed before the manuscript can be accepted:
1. The authors claim to have developed a machine learning (ML) model capable of predicting the physical properties of fuels, including (but not limited to) freezing point. Unfortunately, the details of the model are so sparse that it is impossible to assess the scientific validity of the method. Apart from the mention of PaDEL fingerprints, there is no information provided about the algorithm used to fit the data, the independent variables, or even the Python packages employed. This lack of transparency must be addressed before publication. Additionally, because PaDEL generates 2D fingerprints, these descriptors may not adequately represent compounds with features that differ significantly from those used in training the model.
2. The effect of stereochemistry on freezing point is not discussed in the manuscript. It is well known that irregularities in stereochemical configurations—such as those resulting from replacing double bonds with cyclopropyl groups—can significantly influence freezing points. Irregular or inconsistent chains can reduce the freezing point while maintaining the same energy density, potentially making the fuel more desirable. This aspect warrants discussion.
3. The advantages and limitations of the chosen DFT basis set for geometry optimization are not addressed. Given the unique geometries of the studied compounds, is the B3LYP/6-311G** basis set an appropriate choice? A brief justification of this selection, along with a discussion of potential limitations, would strengthen the manuscript.

Reviewer #4

(Remarks to the Author)
This manuscript reports on the biosynthesis of polycyclopropanated (POP) fatty acids mediated by iterative polyketide synthases (iPKS), building on prior studies of the natural product jawsamycin (Hiratsuka et al., Angew. Chem. Int. Ed., 2014) and the authors' own work on fuelmycin (Cruz-Morales et al., Joule, 2022). As the authors note, this class of molecules shows strong potential as high-energy-density fuels, especially for applications such as aviation where batteries are impractical. To identify desirable molecular features, the authors employ thermochemical estimates of the energy content, and use machine learning to predict melting points of various polycyclopropanated hydrocarbon structures. Key properties of interest include chemical stability, energy density, and melting/freezing point, which require control of the chain length, minimizing the number of double bonds, and maximizing the number of cyclopropane rings. Their bioinformatics study revealed three clades of related POP iPKSs: one associated with jawsamycin (an acyl-pyrimidine conjugate), one with fuelmycin (a free fatty acid), and a third uncharacterized clade. As the enzymes were not amenable to expression in E. coli, the authors undertook a laborious effort to establish a reliable recombinant system in S. coelicolor, which enabled subsequent biochemical studies. This showed that the three iPKS types produce distinct POP fatty acyl products, the first two differing in double bond positions as observed in jawsamycin / fuelmycin, while the third is fully cyclopropanated. Most intriguing are the combinatorial studies of iPKS, cyclopropanase (CP), and thioesterase (TE), which suggest that product

distribution is governed by competition between cyclopropanation, chain elongation, and termination, rationalizing the observed variation in chain length and double bond positions.

Building on previous research, this study provides the enzymatic components and molecular insights needed for controlling the chain length and cyclopropane content of POP fatty acid products, which represents a significant advance toward the biotechnological production of polycyclopropanated fuels. As a unique class of iPKS, the findings also have broad interest for the natural products and mechanistic biochemistry communities. The biochemical experiments and data analyses are rigorous, with interpretations and discussions that are both thoughtful and insightful. Regarding the machine learning section, the potential accuracy of the freezing point predictions is difficult to assess due to the structural uniqueness of the polycyclopropanated compounds compared to other molecules in the training set, but nonetheless provides a useful framework for relating molecular structure to desired properties.

To optimize titers, the authors noticed that an extended fermentation time led to a significant improvement in yields. It is noteworthy that typical fermentation periods for actinobacteria range from 2 to 14 days; have the authors considered experimenting with even longer durations? Additionally, given that time is a critical factor, have previous modifications been assessed at these extended fermentation periods?

For Table 1, the authors assign the structures of the POP-FA products based on LC-MS analysis. However, additional details are needed, particularly regarding how the positions of the double bonds were determined. Further data, such as NMR, would be necessary to confirm the structure of the novel class III POP-FAs.

In Figures 4b and 5c, the chosen color maps make it difficult to distinguish between the different cyclopropanation levels and chain lengths. An alternative color map should be considered for greater contrast.

Figure 4d, 5e, Given the nature of the compounds, further details are essential, particularly concerning the determination of the positions of the double bonds. If these are indeed new compounds in literature, NMR analysis would be preferable.

The paragraph lines 489-505: The results from the chimeric POP-FA strains provide valuable insights into the mechanistic basis of the distinct product distributions. Based on the mechanism proposed by Oikawa et al. (2017), it can be inferred that the balance between iPKS substrate specificities and the rate of cyclopropanation is critical for controlling chain length and the cyclopropane ratio, as illustrated by conclusions from Figures 4, 5, and S14. However, the current description in the manuscript is challenging to follow for audience who have not read the previous study. A schematic illustration would enhance understanding, along with a concise discussion of the necessary kinetic properties of the various PKS components required to achieve the target molecular structure.

Minor suggestions:
Line 136 “we predict these molecules to be precursors for fuels with energy density improvements and comparable freezing points to cyclopropanated myrcene.”
Does this statement refer to the decarboxylated hydrocarbon derivatives? As presented in the introduction, this could be interpreted as referring to the FAME derivative.

Line 153 “...predicted energy density is 45.14 MJ/L, despite having ten fewer main-chain carbons and two fewer cyclopropane rings.”
This statement is misleading, as it seems to imply similar energy content per mole, whereas the numbers are referring to energy content per unit volume.

The paragraph lines 199-209, again this seems to confuse energy content per mole vs. per unit volume. We may expect chain length of fully cyclopropanated hydrocarbon to affect melting point, with minor effect on density and energy content per unit mass or volume.

Line 409 – is Figure S11 the correct reference?

Reviewer #5

(Remarks to the Author)
I co-reviewed this manuscript with one of the reviewers who provided the listed reports. This is part of the Nature Communications initiative to facilitate training in peer review and to provide appropriate recognition for Early Career Researchers who co-review manuscripts.

Version 1:

Reviewer comments:

Reviewer #1

(Remarks to the Author)
Appropriate corrections have been made to comments 1-3. In particular, comment 2 has been suitably corrected by improving the yield through further experiments, collecting additional data, and clearly stating that the position of the double bond could not be identified. I have therefore recommended that this paper be accepted.

Reviewer #2

(Remarks to the Author)
The authors have responded very well to my comments regarding scaling/titers, and I have no further issues. It is very nice work

Reviewer #3

(Remarks to the Author)
My concerns were addressed. I am now ok with publishing this manuscript.

Reviewer #4

(Remarks to the Author)
The addition of LC-MS/MS data substantially strengthens the structural assignments, particularly for the fully cyclopropane-saturated class III products, which represent a central focus of the study. Although full NMR characterization was not feasible, the authors have appropriately moderated their structural claims and clearly acknowledged the limitations regarding double bond positioning. The main conclusions concerning programmable control of chain length and cyclopropane saturation are well supported by the revised data, and the previous concerns have been adequately addressed.

Reviewer #5

(Remarks to the Author)
I co-reviewed this manuscript with one of the reviewers who provided the listed reports. This is part of the Nature Communications initiative to facilitate training in peer review and to provide appropriate recognition for Early Career Researchers who co-review manuscripts.

Open Access This Peer Review File is licensed under a Creative Commons Attribution 4.0 International License, which permits use, sharing, adaptation, distribution and reproduction in any medium or format, as long as you give appropriate credit to the original author(s) and the source, provide a link to the Creative Commons license, and indicate if changes were made.
In cases where reviewers are anonymous, credit should be given to 'Anonymous Referee' and the source.
The images or other third party material in this Peer Review File are included in the article's Creative Commons license, unless indicated otherwise in a credit line to the material. If material is not included in the article's Creative Commons license and your intended use is not permitted by statutory regulation or exceeds the permitted use, you will need to obtain permission directly from the copyright holder.

To view a copy of this license, visit https://creativecommons.org/licenses/by/4.0/

Reviewer #1 (Remarks to the Author):

In developing new jet fuel, authors have been conducting research based on the advantages of microbial polycyclopropanated fatty acids (POP-FA). In 2022, they succeeded in genome mining of a known jawsamycin-like BGC and found a promising research target named fuelimycin. Following this finding, in this study, they initially developed a prediction program to identify suitable POP-FA, that hardly solidify at low temperatures and has high energy density, is C12:CP5 or C14:CP6, and set the goal of producing them as the research goal.

In addition to BGCs for the two POP-FAs, fuelimycin (class I) and jawsamycin (class II), authors added the third BGC from S. longispororuber, which produces a new POP-FA (Class III) in which all unsaturated bonds of the parent polyunsaturated fatty acid are cyclopropanated. Using this three-type iPKS system, they tried to elucidate the complex regulatory mechanism of POP-FA formation. Since the iPKS system involved in the production of POP-FA has independent enzymatic functions for CP, KR, and TE, they succeeded in preparing strains that produce the desired POP-FAs by repeatedly swapping each domain of the three BGCs.

Although the mechanism by which POP-FA skeletal construction is carried out has not yet been fully elucidated, important academic discoveries have been made in the course of this research that give rise to expectations for further developments in the future, including that the iPKS system that gives rise to the POP structure is not an equimolar complex, but that the reaction proceeds with a high iPKS/CP ratio, and that the two domains, stand-alone iPKS/CP, play a central role in controlling the chain length and number of cyclopropanation.

The paper is clearly written, properly cited, and the data is of high quality. I believe this manuscript provides sufficient novelty and rigor to warrant publication in Nature Communications and would have impact in the natural products field and garner general interest from synthetic biologists. I have a few suggestions and comments I would ask the authors to address. Please find my suggestions below.

Comments

1) In this paper, unlike ref. 24, all calculations of titers and structure determinations were based on LC-MS data. All discussions of swapping experiments use this data, so referring to ref. 24 is insufficient. It is necessary to clarify whether the experiment was performed using crude extracts or after enriching POP-FA fractions. In addition, the pretreatment procedures for the samples to be analyzed should also be described.

RESPONSE:

- We thank the reviewer for this comment; these experiments were performed using crude extracts. We have made revisions to clarify in the Methods Line 709 that

"The remaining dense liquid product was resuspended in methanol, vortexed for 10 min and incubated at 20 °C for 8-12 h before solids were separated by centrifugation and removed. These crude extracts were analyzed using the previously reported LC-MS method".

- In the meantime, we have performed additional experiments to support our discussions of titers and structure determination, as we detail in our later responses.

2) Regarding the structure determination, the data presented in the SI (Figures S16-S24) can be considered to be inferred only from the ion chromatogram and HR-MS data for M+. It cannot be denied that the CP skip may really occur on a site other than the carboxyl group, and I believe that the structure of the new compounds needs to be determined by LC-MS/MS analysis as shown in the SI of ref. 24. Furthermore, utilizing UV data may help to determine the terminal conjugated system by the absorption wavelength, which may enable more accurate identification of the position of the double bonds.

RESPONSE:

- We thank the reviewer and agree with this insight. In our original study (ref. 24), LC-MS/MS analysis of class I POP-FAs was carried out using crude extracts from large amounts of PPPP cultures (up to 300 L). Such a large volume of cultures was needed due to the low POP-FA titers (42-94 µg/L) and the nature of a highly mixed POP-FA product profile. While we cannot examine our hybrid strain products at a similar large scale, we have performed additional production studies (in shake flasks of 500 mL combined total volumes or 1-L bioreactors, (**Figure S12**))

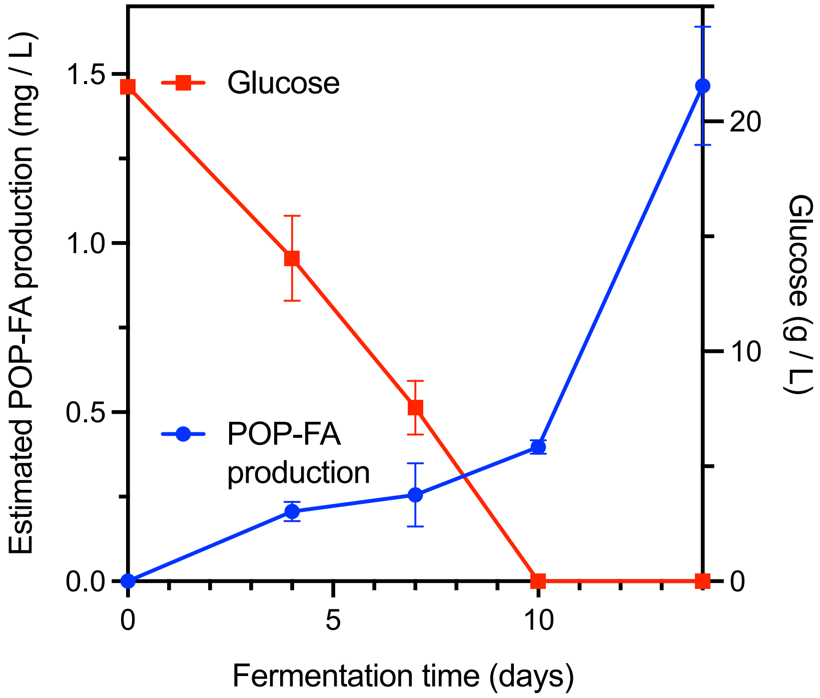

-

Figure S12. POP-FA production and glucose consumption by strain KY16 (PLPP) in bioreactors. Although cell growth was not accurately measured via OD_{600} due to flocculation, *Streptomyces coelicolor* likely reached stationary phase by day 4, during which POP-FA production temporarily stagnated. After glucose depletion and a corresponding switch towards secondary metabolite production (Wang et al. 2019), POP-FA production increases again. All data are presented as mean values; error bars indicate the s.d. of biological replicates (*n* = 3).

and LC-MS/MS analysis of crude extracts from JJJP, PJPP, and PLPP in an effort to identify the CP skip/double bond positions for class II and class III POP-FAs. We have also included two new coauthors who contributed to these experiments in the updated manuscript.

- Based on the editor's and reviewer No.4's suggestions, we first increased typical production time from 4 days to 14 days, resulting in up to 18-fold increase in POP-FA titers. The significant titer increase enabled us to drastically decrease the culture volume needed for generating enough crude extracts for LC-MS/MS analysis. As we performed LC-MS/MS analysis, we found that the relative productivity of JJJP and PJPP pathways was still too low to reliably obtain fragmentation data for characterizing class II (jawsamycin) POP-FA structures. Furthermore, as demonstrated in Figure S19, the TE domain does not notably affect pathway product profiles. This is a strong indication that the JJJP hybrid pathway (in strain KY4) should not produce a different polyketide structure than the native jawsamycin polyketide structure as previously elucidated [1–3]. For this manuscript, we thus prioritized elucidating the previously uncharacterized log2 CP homolog on cyclopropane position. As shown in Figure 5, LLLL product titer was only 10% of that achieved using PLPP, thus we focused on the scaled up production and LC-MS/MS characterization of PLPP products, which also included class III POP-FAs of great interest given both their shorter chain lengths and higher cyclopropane saturation levels.
- We thus optimized our LC-MS/MS analysis of PLPP products over multiple rounds of PLPP production studies. Initially, as a result of high collision energies, our MS/MS data were too fragmented to resolve any class III POP-FA structures, as illustrated in the Figure_Response 1 below.

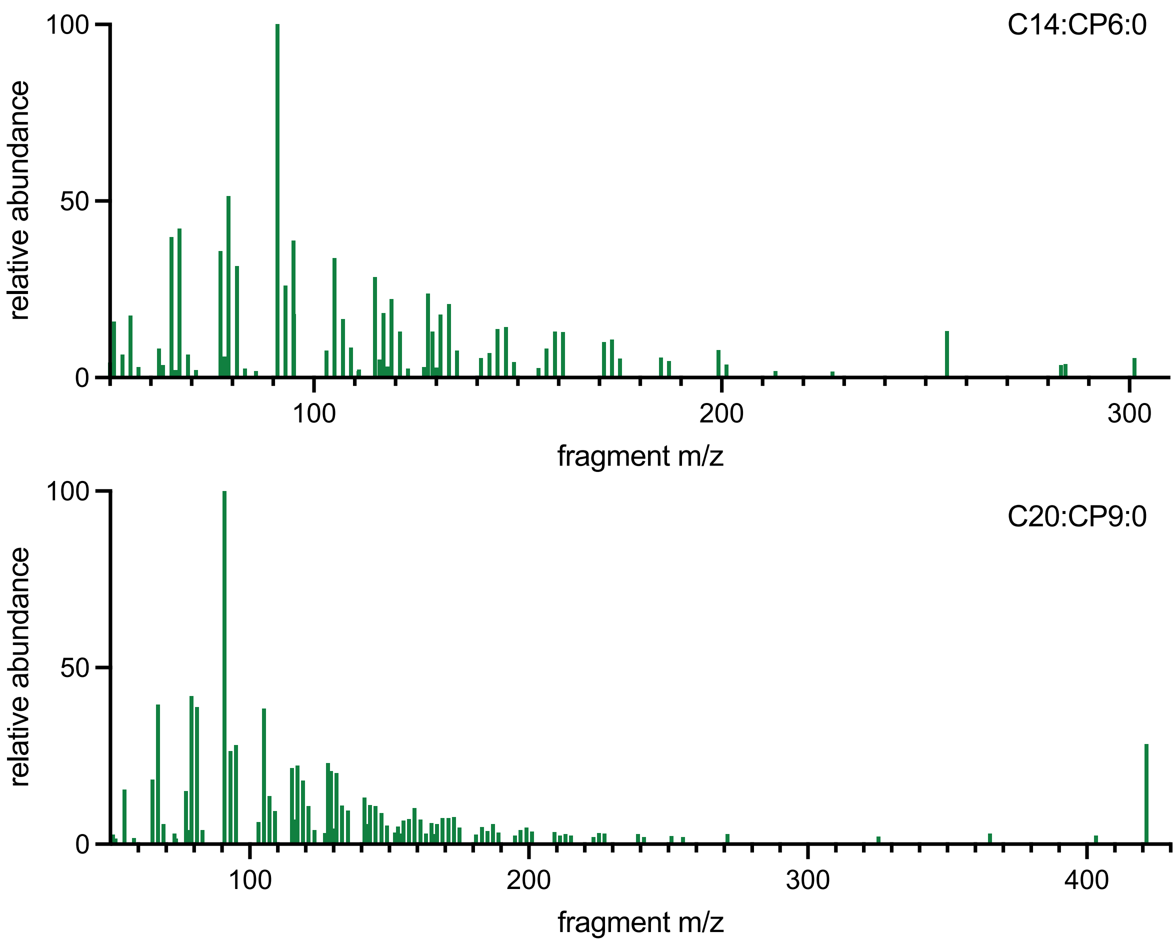

Figure_Response 1. Initial MS/MS analysis of PLPP products. Due to high collision energies, a significant majority of fragment ions had low m/z values from which the structures of class III POP-FAs could not be validated.

- After decreasing the collision energy, we observed ions with lower signal intensity, but with m/z values that validate our previously uncharacterized, fully cyclopropanated class III POP-FAs, e.g. C14:CP6, C16:CP7, C18:CP8, and C20:CP9 (now in updated **Figures S15-18**). We also partially validate unsaturated products from PLPP, however the double bond position remains unsolved (now in updated **Figures S19-22**) as various double bond positions produce similar fragmentation patterns. Our current evidence suggests the possibility of a mixture of class I, class II, and potentially new classes of POP-FAs among the unsaturated products of PLPP.
- Additionally, as we optimized our LC methods (**Table S9**), we observed increasingly split chromatogram peaks that may correspond to class I and class II structural isomers resulting from different double bond positions; it is noteworthy that the EICs of POP-FAs that are saturated with CP rings and thus lack double bonds or any possibility for structural isomers,

retained clean singular peaks as illustrated in Figure_Response 2. To confirm this hypothesis, class II jawsamycin POP-FA standard is needed for further LC peak interpretation; we attempted to obtain a class II POP-FA standard, but none were available.

- Finally, following the reviewer's suggestion, we examined the 214, 254, and 280 nm UV absorbances in LC analysis, but failed to detect signals corresponding to the POP-FA terminal conjugated system, possibly partly due to relatively low POP-FA titers.
- We have thus revised the language of the manuscript to clarify that the proposed structure of class II POP-FAs requires further experimental confirmation:
    - Abstract Line 42-47: "Leveraging both natural and engineered pathway product diversity, we demonstrate targeted production of new POP-FAs, namely shortened POP-FAs with predicted superior freezing point properties for aviation, as well as fully cyclopropane-saturated POP-FAs which should have superior energy-density. These precise and controllable modifications to POP-FA structure open the door for bioproduction of designer POP fuels."
    - Line 376-377: "We name the products of this modified jawsamycin pathway (JJJP), which we expect would share the skipped-cyclopropane pattern, class II POP-FAs."
    - Line 476-489: "Given its combined titer, cyclopropanation, and chain length advantages, we selected strain KY16 (PLPP) for 14-day cultivations (**Figure 3F**), scaled up to 3 liters, for the purpose of HPLC purification and structural characterization of products. In bioreactor format, we again observed a late-stage production increase corresponding to glucose depletion[4] (**Figure S12**); however, relative production titers remained higher when strains were cultivated in flasks. Based on our standard curves (**Table S1; Figures S13-14**), strain KY16 produced an estimated 5.40 - 6.24 mg/L total POP-FAs in 50 mL flask cultures and 1.37 - 1.58 mg/L total POP-FAs in 1 L bioreactor cultures. Subsequent LC-MS/MS analysis resulted in fragmentation spectra that confirm the cyclopropane-saturated structures of class III POP-FAs (**Figures S15-S18**); however, our MS/MS analysis confirmed only the number and not the position of carbon-carbon double bonds in unsaturated products from KY16 (PLPP) (**Figures S19-22**). In total, our engineered strains present an increase to POP-FA diversity from 6 to at least 18 distinct molecules (**Table 1; Table S5**), without including potential variations in carbon-carbon double bond position."
    - Line 508-510: "This further supports that the addition of a POP-TE into the jawsamycin pathway, e.g., strain KY4 (JJJP), should

produce class II POP-FAs and not modify the distinct cyclopropanation pattern of jawsamycins."

- Line 611-613: "By contrast, we have systematically shown that a POP-iPKS pathway can be engineered towards targeted chain lengths, cyclopropane saturation levels, and potentially cyclopropane positions."

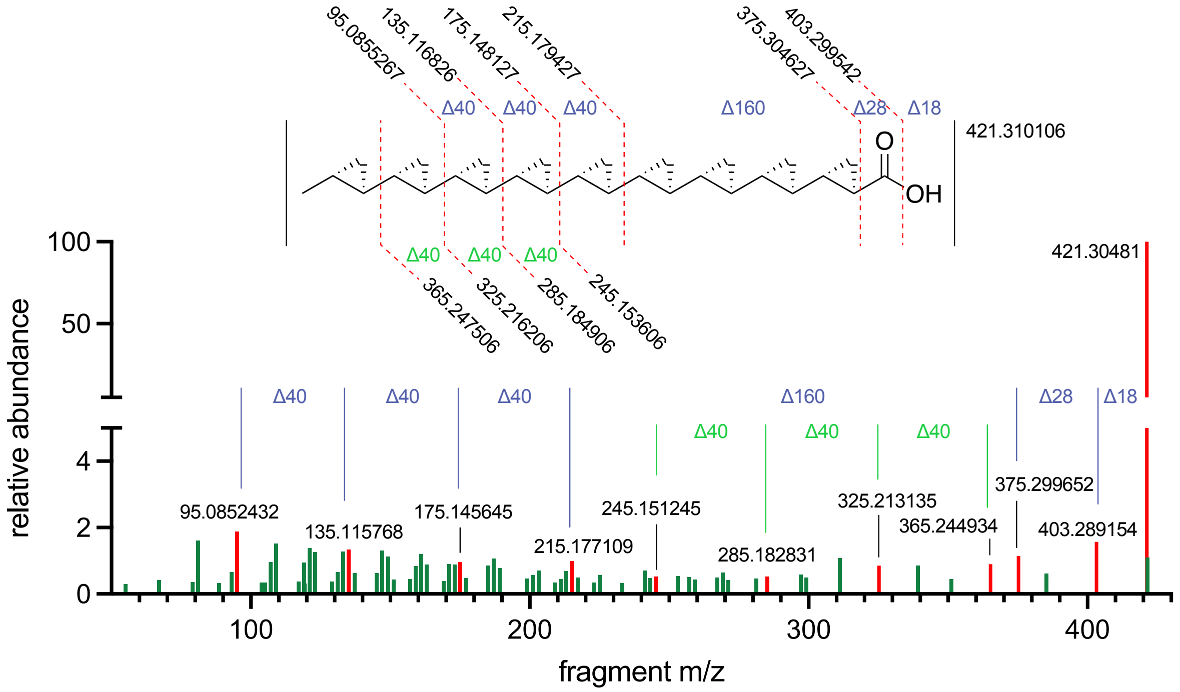

Figure S15. Structural prediction of C20:CP9 using LC-MS/MS. The predicted structure of C20:CP9 is shown on top, with mass shifts shown between fragmentation sites. Fragments corresponding to ions detected are indicated with dashed lines. The full spectrum is shown in green with the corresponding ions highlighted in red.

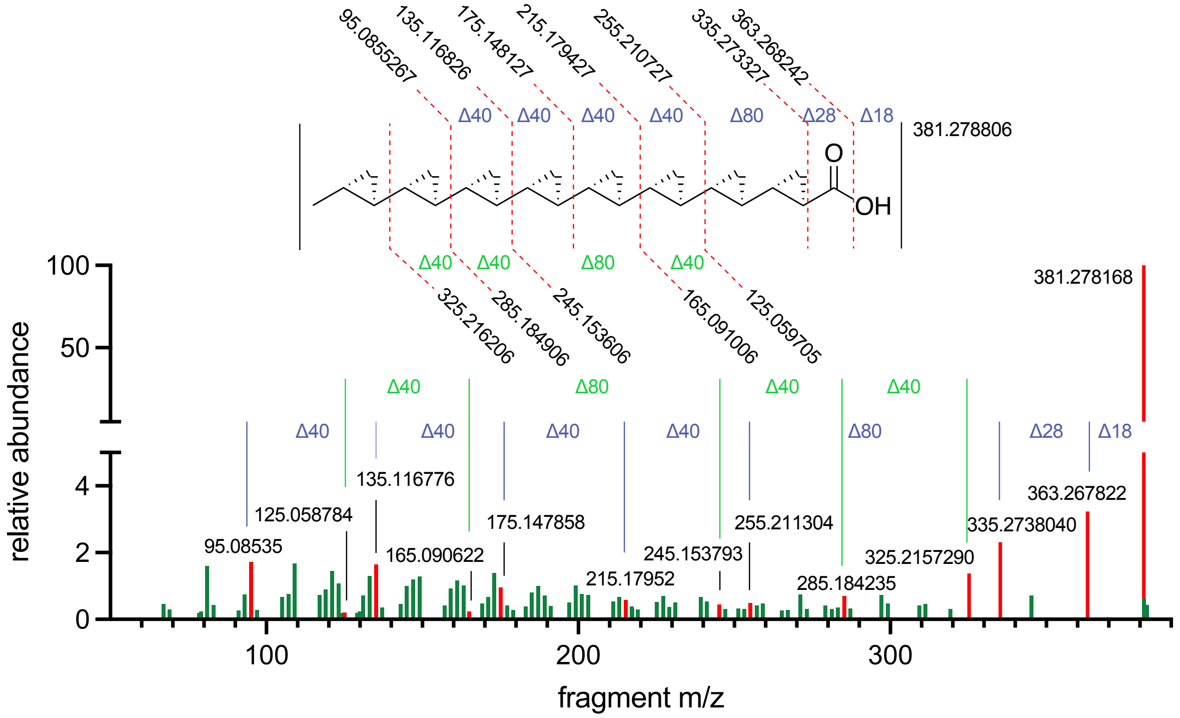

Figure S16. Structural prediction of C18:CP8 using LC-MS/MS. The predicted structure of C18:CP8 is shown on top, with mass shifts shown between fragmentation sites. Fragments corresponding to ions detected are indicated with dashed lines. The full spectrum is shown in green with the corresponding ions highlighted in red.

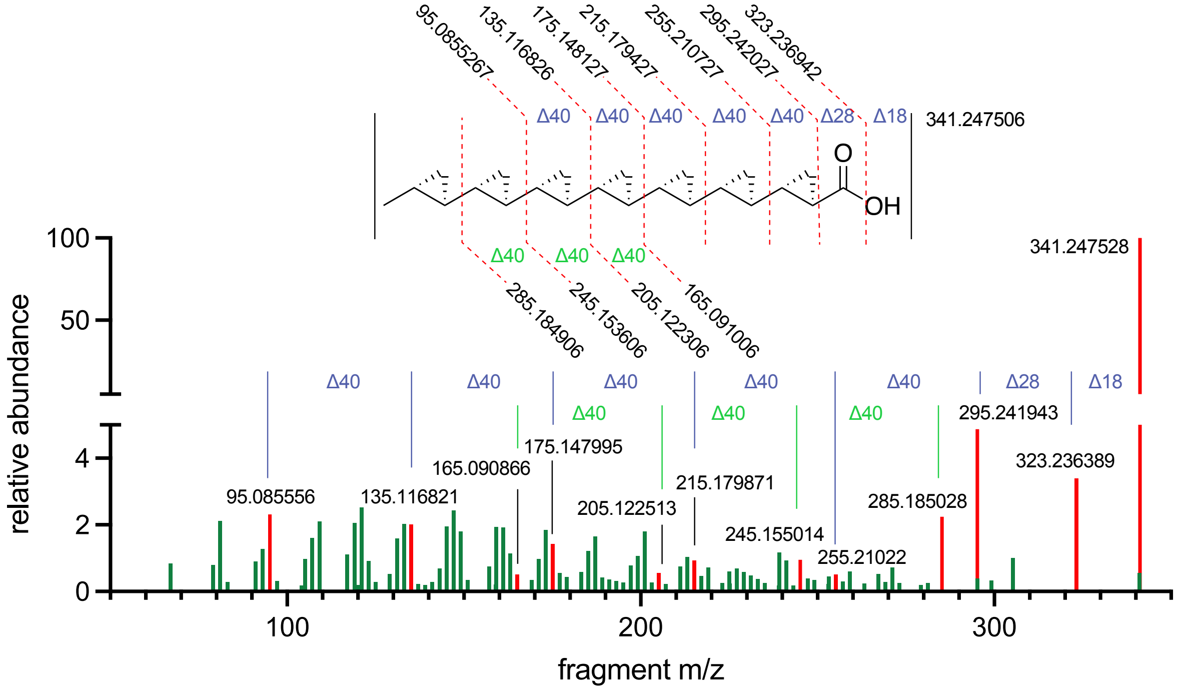

Figure S17. Structural prediction of C16:CP7 using LC-MS/MS. The predicted structure of C16:CP7 is shown on top, with mass shifts shown between fragmentation sites. Fragments corresponding to ions detected are indicated with dashed lines. The full spectrum is shown in green with the corresponding ions highlighted in red.

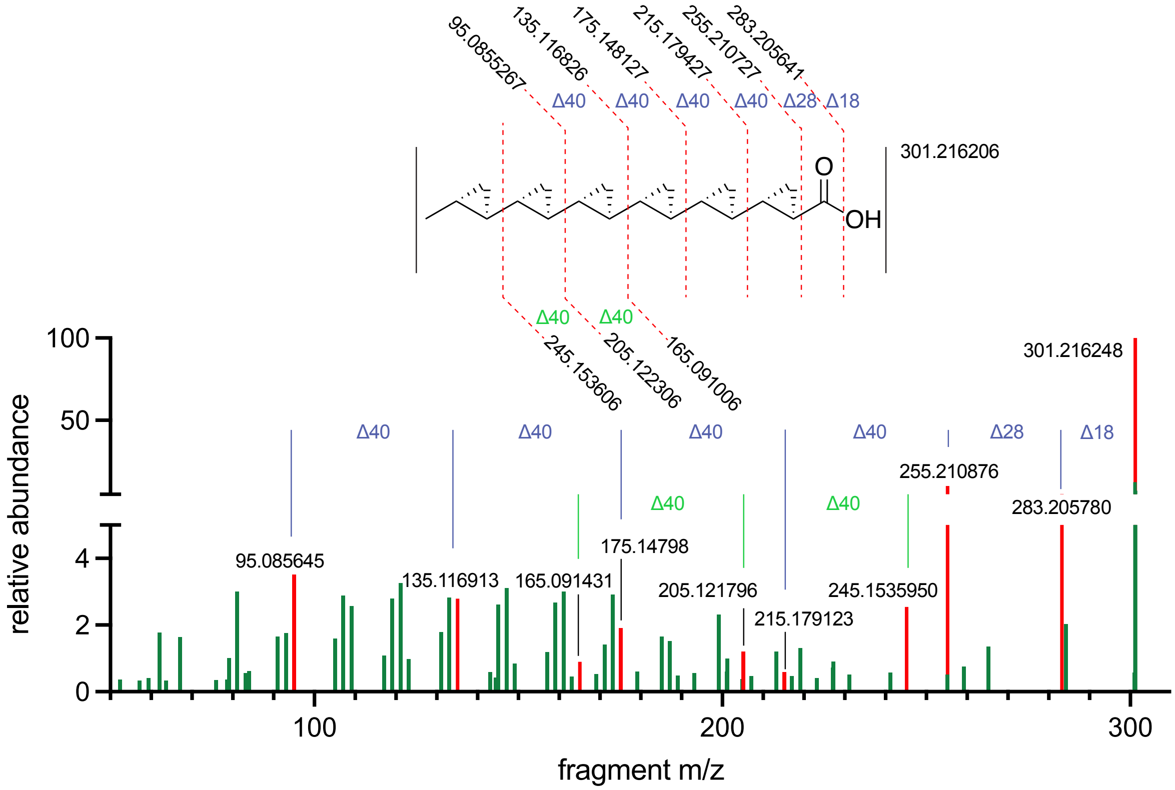

Figure S18. Structural prediction of C14:CP6 using LC-MS/MS. The predicted structure of C14:CP6 is shown on top, with mass shifts shown between fragmentation sites. Fragments corresponding to ions detected are indicated with dashed lines. The full spectrum is shown in green with the corresponding ions highlighted in red.

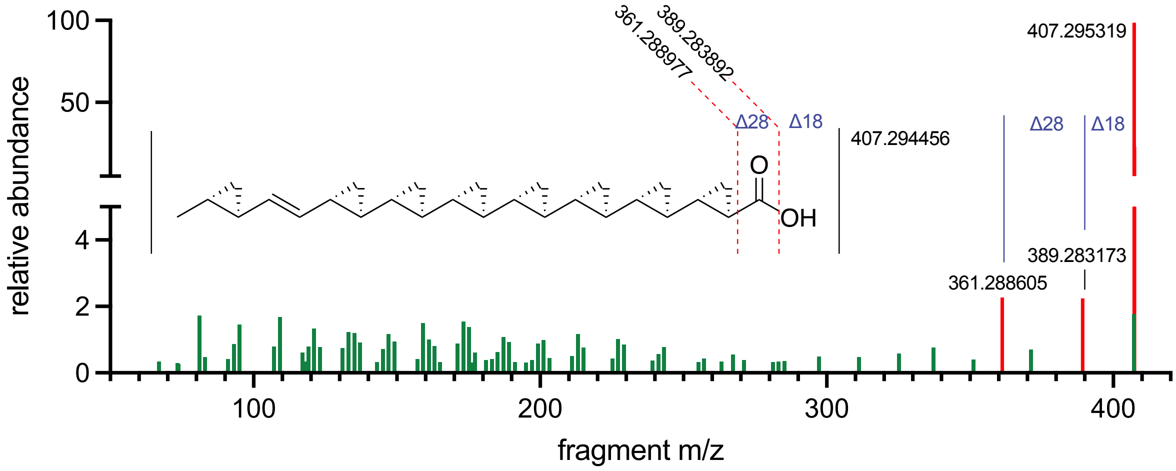

Figure S19. Partial structural prediction of C20:CP8 using LC-MS/MS. The partial predicted structure of C20:CP8 is shown on top, with mass shifts show between fragmentation sites. The position of the carbon-carbon double bond is hypothetical and not determined experimentally. Fragments corresponding to ions detected are indicated with dashed lines. The full spectrum is shown in green with the corresponding ions highlighted in red.

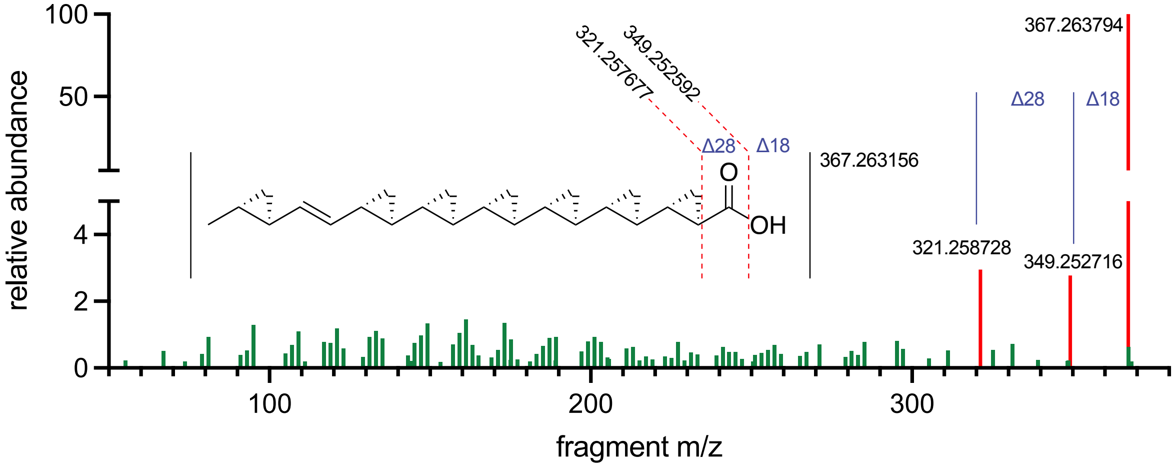

Figure S20. Partial structural prediction of C18:CP7 using LC-MS/MS. The partial predicted structure of C18:CP7 is shown on top, with mass shifts show between fragmentation sites. The position of the carbon-carbon double bond is hypothetical and not determined experimentally. Fragments corresponding to ions detected are indicated with dashed lines. The full spectrum is shown in green with the corresponding ions highlighted in red.

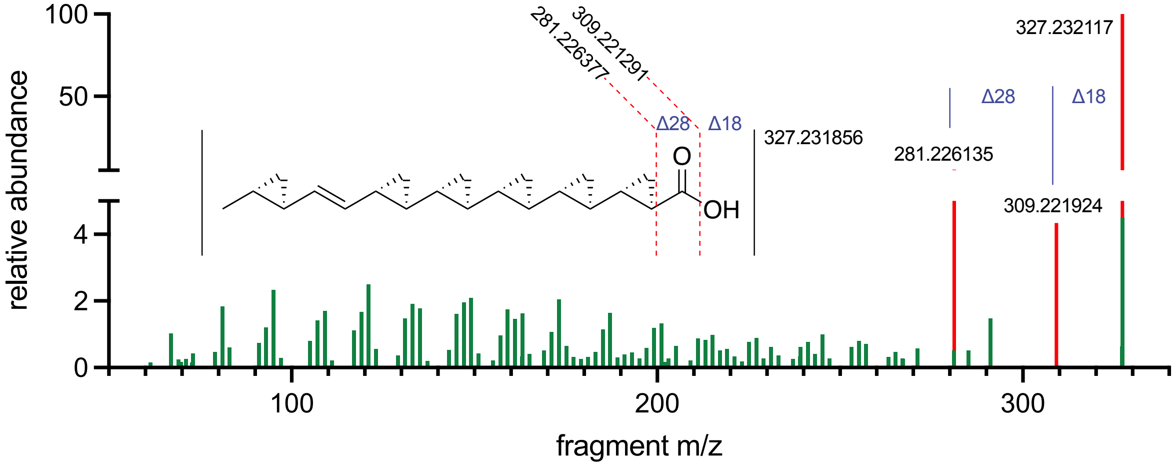

Figure S21. Partial structural prediction of C16:CP6 using LC-MS/MS. The partial predicted structure of C16:CP6 is shown on top, with mass shifts show between fragmentation sites. The position of the carbon-carbon double bond is hypothetical and not determined experimentally. Fragments corresponding to ions detected are indicated with dashed lines. The full spectrum is shown in green with the corresponding ions highlighted in red.

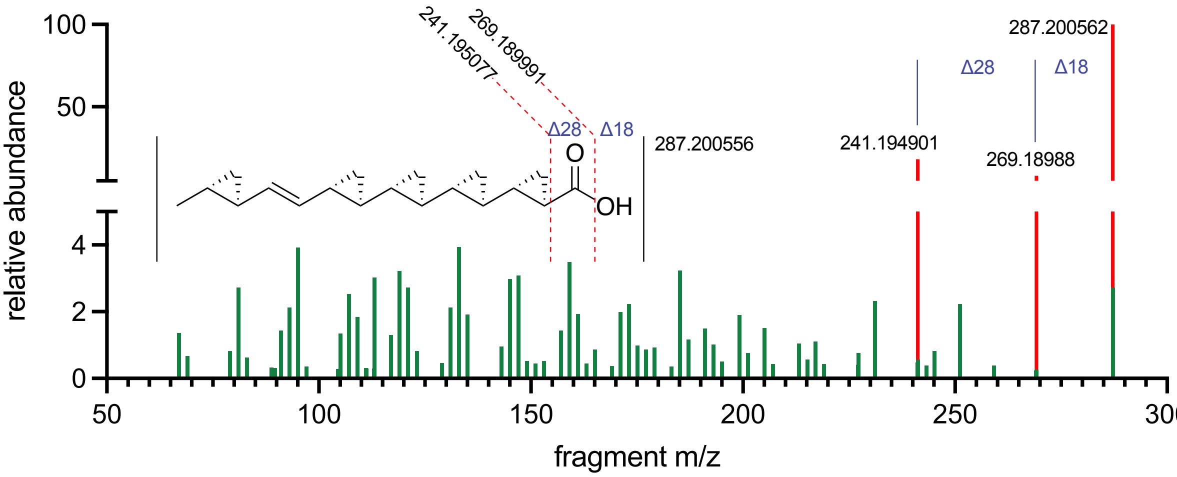

Figure S22. Partial structural prediction of C14:CP5 using LC-MS/MS. The partial predicted structure of C14:CP5 is shown on top, with mass shifts show between fragmentation sites. The position of the carbon-carbon double bond is hypothetical and not determined experimentally. Fragments corresponding to ions detected are indicated with dashed lines. The full spectrum is shown in green with the corresponding ions highlighted in red.

Chemical Structure	Name	KY4 (JJJP)	KY5 (LLLL)	KY16 (PLPP)
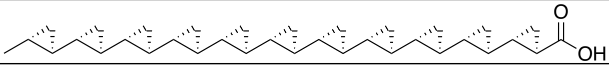	C24:CP11	-	+	-
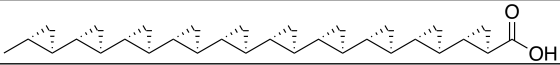	C22:CP10	-	+	-
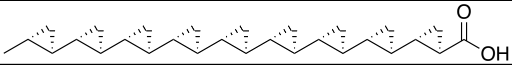	C20:CP9	-	+	+
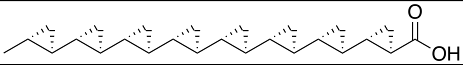	C18:CP8	-	+	+
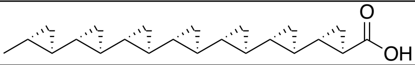	C16:CP7	+	+	+
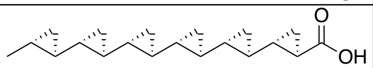	C14:CP6	+	-	+
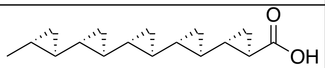	C12:CP5	-	-	+

Table 1. Cyclopropane-saturated (class III) POP-FAs produced in this study.

Class	Chemical Structure	Name	POP3.4 (PPPP)	KY4 (JJJP)	KY5 (LLLL)	KY16 (PLPP)
Class I 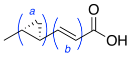	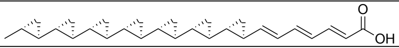	C22:CP7	+	-	-	-
	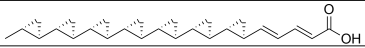	C20:CP7	+	-	-	?
	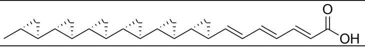	C20:CP6	+	-	-	?
	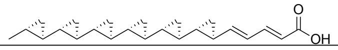	C18:CP6	+	-	-	?
	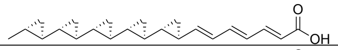	C18:CP5	+	-	-	?
	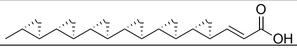	C16:CP6	+	-	-	?
Class II 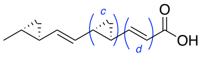	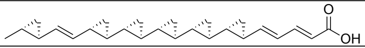	C20:CP6	-	+	-	?
	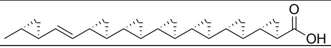	C18:CP7	-	+	-	?
	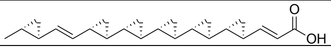	C18:CP6	-	+	-	?
	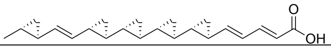	C18:CP5	-	+	-	?
	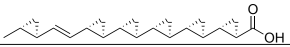	C16:CP6	-	+	-	?
	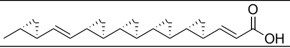	C16:CP5	-	+	-	-
	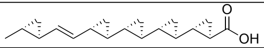	C14:CP5	-	+	-	?
	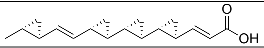	C14:CP4	-	+	-	-
	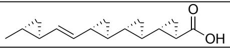	C12:CP4	-	+	-	-
Class III 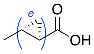	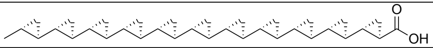	C24:CP11	-	-	+	-
	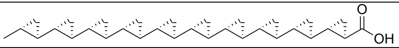	C22:CP10	-	-	+	-
	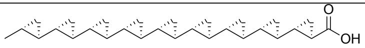	C20:CP9	-	-	+	+
	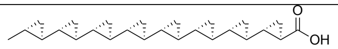	C18:CP8	-	-	+	+
	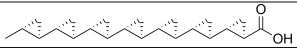	C16:CP7	-	+	+	+
	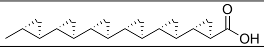	C14:CP6	-	+	-	+
	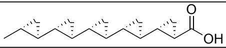	C12:CP5	-	-	-	+

Table S5. Proposed POP-FA natural diversity and classifications.
Class I POP-FAs produced by POP3.4 (PPPP) strain contain no cyclopropanation skips [5]. Class II POP-FAs produced by KY4 (JJJP) strain may contain the same distinctive cyclopropanation skip in the second ketide unit as characterized in jawsamycin [1–3]. Class III POP-FAs predominantly produced by KY5 (LLLL) and KY16 (PLPP) strains, with minor production from KY4 (JJJP), are fully cyclopropane-saturated. Unsaturated products from KY16 (PLPP) have unknown carbon-carbon double bond positions and may be class I, class II, and/or an undescribed class. All strain information can be found in Table S6.

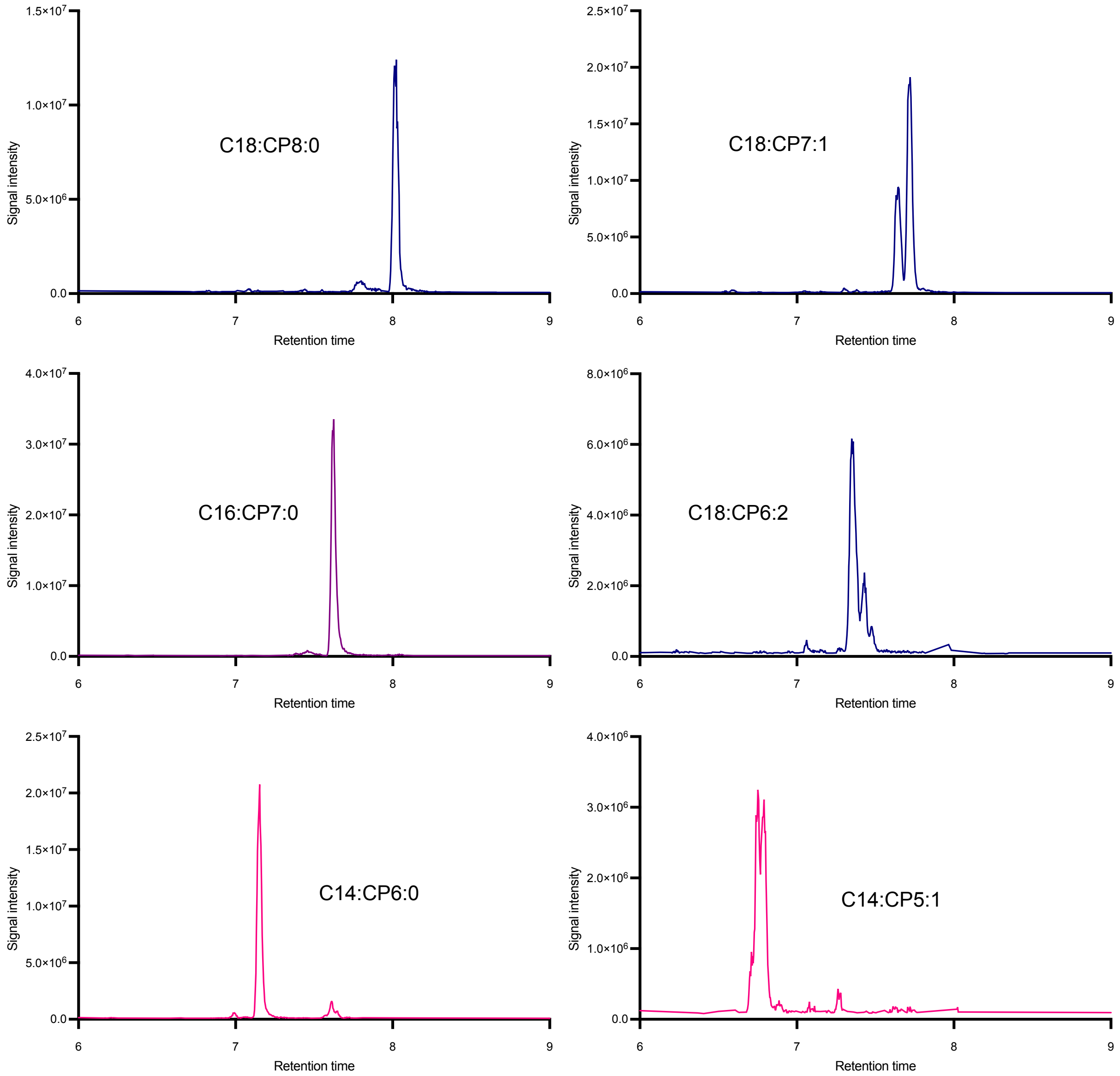

Figure_Response 2. Extracted Ion Chromatograms of POP-FAs from PLPP. POP-FAs with one or more carbon-carbon double bonds were observed to have split peaks, which may signify structural isomers as a result of differing double bond position. Class III POP-FAs lack carbon-carbon double bonds and thus are not expected to have any structural isomers.

3) Regarding the titer, Supplemental Table 1 lists three types of internal standard substances. Without detailed explanation, it is difficult to understand how to use the three types of standard substances. In what concentration range can reliable data be obtained? In particular, the peaks of trace components are split in a complex manner, and it would be necessary to show how they are processed to calculate accurate peak areas. In ref. 24, a standard POP-FAs fuelimycins were isolated and characterized, using these samples, it seems possible to verify the validity of the use of the three standard substances.

RESPONSE:

- We agree that the three listed internal standards are not optimal for the measurement of fuelimycin titers.
- However, in ref. 24, fuelimycins were only partially purified and thus could not be used as a preferred standard. Therefore, to address these concerns, we prepared standard curves for ARA and DCP-LA from 1 mg/L to 200 mg/L, corresponding to the fuelimycin concentration range we expect from our crude extracts. Furthermore, previously the peak areas for our standard substances fluctuated between experiments despite using the same LC-MS methods and instrumentation; we reasoned that this was a consequence of our sample preparation method (via dilution of a concentrated ARA/DCP-LA stock solution). For more precise standard curves, we more accurately prepared our samples with direct weight measurements.

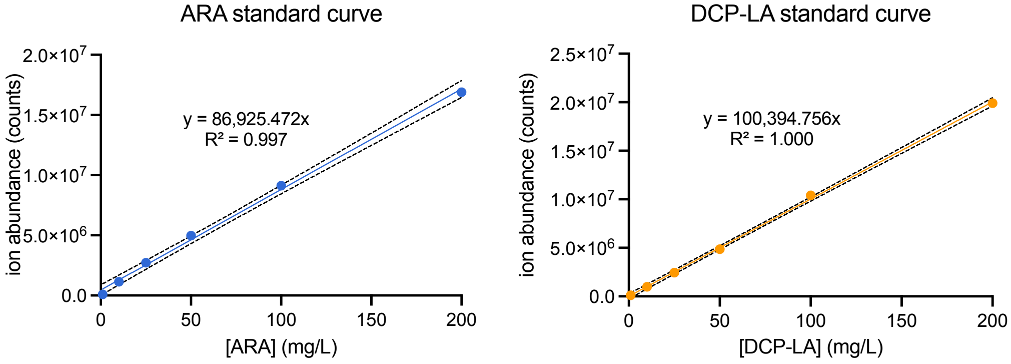

- We observed that within this range, an ARA standard curve with an equation of:

$$sample\ concentration\ = \frac{peak\ area}{86925.472}$$

could provide an estimate within an order of magnitude for the concentration of cyclopropanated fatty acids like DCP-LA:

Table_Response 1.

Measured DCP-LA (mg/L)	DCP-LA estimate from ARA standard curve (mg/L)
1	1.32
10	11.42
25	28.18
50	55.95
100	120.10
200	229.23

- We have updated Supplementary Table 1 based on our standard curve results. As the reviewer described, the peaks of trace components are split and complex; thus, we revised the manuscript to more frankly reflect that for minor products, we accepted a rough estimate of peak area, as they do not impact total titer nor

product profile estimates as significantly as the major products with simpler peaks and more reliable peak area calculations. As we added in Line 714:

"[5] of each POP-FA product was calculated by their extracted-ion chromatogram (EIC) peak area; because minor products do not impact total titer nor product profile estimates as significantly as major products, we accepted a rough estimate of minor product abundances when their EICs were more complex or contained split peaks."

Minor points

P13, Figure 5 caption; (pop1, log2, pop3, pop4; PLPP). Pop should be Pfl.

RESPONSE:

- We thank the reviewers for catching this error; we have corrected it to Pfl.

Reviewer #2 (Remarks to the Author):

In this manuscript, Yin and coworkers investigate the determinants of chain length, degree of cylclopropanation, and saturation of polycyclopropanated (POP) fuel molecules produced in an engineered Streptomyces strain by an iterative polyketide synthase (iPKS). This work is a nice follow-up of this group's discovery of the encoding biosynthetic gene clusters a few years ago. They start by computationally predicting fuel energy density and freezing point of modified POPs to guide modifications to the pathway to improve these properties, identifying shorter chains and a higher degree of cylclopropanation as targets. Based on this, they combinatorially profile pathway variants with different iPKSs, cyclopropanases, and thioesterases to demonstrated production of cyclopropane-saturated molecules. Overall, the work is comprehensive, well executed, the results clearly presented, and the narrative logical. The authors are applauded for a high-quality, rigorous study. My primary concern is the impact of the work, as described below.

The authors indicate (correctly) that the identified molecules are some of the most energy-dense molecules produced in a biological system. However, the titer is so low that they cannot even be structurally characterized (estimated 42-94 µg/L, Table S1). And, they are always produced as a mixture of several different chain lengths. This is despite quite a bit of effort on the part of the authors to improve titer, for example by exploring different hosts, refactoring the gene cluster, supplementing PQQ, and extending the fermentation time. Given this, it is hard to imagine that these could be successfully scaled to produce aviation fuel molecules economically.

As such, while the biosynthetic aspects of the study are highly interesting, the broader impact this work will have on sustainable energy is questionable. The authors acknowledge this in a couple of sentences in the Discussion, but I think that: 1) the titers

achieved should be more openly reported in the main manuscript so the reader can more easily get a sense of the current state of the technology, and 2) a more frank and extensive discussion about whether it will even be possible to achieve the g/L scales needed for this to be commercially viable should also be included.

RESPONSE:

- We thank the reviewers for this feedback. As the reviewers acknowledged, these polycyclopropanes and the control aspects of their iterative biosynthesis remain interesting to study from a purely scientific standpoint. Nevertheless, to address questions regarding feasibility as an aviation fuel, we scaled up fermentations to 3 Liters for 14-days and achieved an estimated titer of ~1 mg/L. These current titers are still exceedingly below commercial viability, and would require an increase of at least 1000-fold to reach a g/L scale. Although this is a significant metabolic engineering challenge, such 1000-fold titer increases [6–8], have been previously achieved. Furthermore, heterologous production on the g/L scale has been demonstrated in *Streptomyces* [9] as well as in other production hosts.[10] We have integrated this discussion into the main text of the manuscript beginning in Line 591 for better clarity.

Reviewer #3 (Remarks to the Author):

This manuscript addresses the important issue of generating efficient bio-derived fuels with high energy content. The authors' approach to solving this problem is clearly innovative and warrants publication. However, there are several deficiencies that must be addressed before the manuscript can be accepted:

1. The authors claim to have developed a machine learning (ML) model capable of predicting the physical properties of fuels, including (but not limited to) freezing point. Unfortunately, the details of the model are so sparse that it is impossible to assess the scientific validity of the method. Apart from the mention of PaDEL fingerprints, there is no information provided about the algorithm used to fit the data, the independent variables, or even the Python packages employed. This lack of transparency must be addressed before publication. Additionally, because PaDEL generates 2D fingerprints, these descriptors may not adequately represent compounds with features that differ significantly from those used in training the model.

RESPONSE:

- We developed an ensemble-based machine learning model (XGBoost) to predict the melting point. The features (collected using PaDEL) are passed through a pipeline of data wrangling processes (for instance, handling missing data, feature engineering, etc.) before being fed to the model. The MAE error (the absolute difference between real and inferred data) filters the compounds whose MAEs are very close. The model is retrained using the filtered dataset whose errors (MAE) are very close to each other to improve the overall inference loss. The details of

the method applied are discussed in great detail in [11](10.26434/chemrxiv-2024-0jfmb).

2. The effect of stereochemistry on freezing point is not discussed in the manuscript. It is well known that irregularities in stereochemical configurations—such as those resulting from replacing double bonds with cyclopropyl groups—can significantly influence freezing points. Irregular or inconsistent chains can reduce the freezing point while maintaining the same energy density, potentially making the fuel more desirable. This aspect warrants discussion.

RESPONSE:

- Thanks for pointing this out. We have added some discussion on how stereochemistry affects freezing point from Lines 200-217:
  "The stereochemistry of a molecule can have profound effects on its melting point. Rotational isomers without bulky chemical groups are able to effectively access all rotational degrees of freedom. These molecules can have lower melting points because it can be harder to adopt a preferred melting point orientation until the temperature drops low enough that molecules are able to align and form crystals. Those with bulky groups can only sample a restricted range of rational angles and thus can more easily conform to a melting point orientation at higher temperature. A simple example of this can be seen from analyzing the trend in melting points for halo-ethanes. The melting points of fluoroethane, chloroethane, bromoethane, and iodoethane are -142.8°C, -138.7°C, -119°C, and -111.1°C, respectively. As the halo group becomes bulkier, the melting point increases because steric hindrance does not allow for free rotation of the CH2-X group, and the preferred orientation for melting is more easily obtained. Of course, there are many other factors that can affect melting point, like atomic charge and dipole moments, and this trend in melting point as a function of bulky chemical groups is only meant to illustrate one factor that affects the melting point of rotational isomers. Our melting point model is trained on a large dataset with a variety of rotational isomers. However, when making predictions it takes in a SMILES notation, which does not include rotational isomer data, and so only takes into account rotational isomerism implicitly."

3. The advantages and limitations of the chosen DFT basis set for geometry optimization are not addressed. Given the unique geometries of the studied compounds, is the B3LYP/6-311G** basis set an appropriate choice? A brief justification of this selection, along with a discussion of potential limitations, would strengthen the manuscript.

RESPONSE:

- A brief description of the DFT method and basis set was added to the manuscript, Lines 739-745:
  "B3LYP was developed in 1993 and has enjoyed wide success in predicting accurate molecular geometries, even for strained ring systems.[12–15] While the basis set chosen can have an effect on molecular geometry, research indicates that, in most cases, the choice of basis set has only a small impact on molecular

geometry.[16,17] The 6-311G** basis set is a split valence triple ζ basis set, and was chosen as a compromise between efficiency and accuracy."

Reviewer #4 (Remarks to the Author):

This manuscript reports on the biosynthesis of polycyclopropanated (POP) fatty acids mediated by iterative polyketide synthases (iPKS), building on prior studies of the natural product jawsamycin (Hiratsuka et al., Angew. Chem. Int. Ed., 2014) and the authors' own work on fuelmycin (Cruz-Morales et al., Joule, 2022). As the authors note, this class of molecules shows strong potential as high-energy-density fuels, especially for applications such as aviation where batteries are impractical. To identify desirable molecular features, the authors employ thermochemical estimates of the energy content, and use machine learning to predict melting points of various polycyclopropanated hydrocarbon structures. Key properties of interest include chemical stability, energy density, and melting/freezing point, which require control of the chain length, minimizing the number of double bonds, and maximizing the number of cyclopropane rings. Their bioinformatics study revealed three clades of related POP iPKSs: one associated with jawsamycin (an acyl-pyrimidine conjugate), one with fuelmycin (a free fatty acid), and a third uncharacterized clade. As the enzymes were not amenable to expression in E. coli, the authors undertook a laborious effort to establish a reliable recombinant system in S. coelicolor, which enabled subsequent biochemical studies. This showed that the three iPKS types produce distinct POP fatty acyl products, the first two differing in double bond positions as observed in jawsamycin / fuelmycin, while the third is fully cyclopropanated. Most intriguing are the combinatorial studies of iPKS, cyclopropanase (CP), and thioesterase (TE), which suggest that product distribution is governed by competition between cyclopropanation, chain elongation, and termination, rationalizing the observed variation in chain length and double bond positions.

Building on previous research, this study provides the enzymatic components and molecular insights needed for controlling the chain length and cyclopropane content of POP fatty acid products, which represents a significant advance toward the biotechnological production of polycyclopropanated fuels. As a unique class of iPKS, the findings also have broad interest for the natural products and mechanistic biochemistry communities. The biochemical experiments and data analyses are rigorous, with interpretations and discussions that are both thoughtful and insightful. Regarding the machine learning section, the potential accuracy of the freezing point predictions is difficult to assess due to the structural uniqueness of the polycyclopropanated compounds compared to other molecules in the training set, but nonetheless provides a useful framework for relating molecular structure to desired properties.

To optimize titers, the authors noticed that an extended fermentation time led to a significant improvement in yields. It is noteworthy that typical fermentation periods for actinobacteria range from 2 to 14 days; have the authors considered experimenting with

even longer durations? Additionally, given that time is a critical factor, have previous modifications been assessed at these extended fermentation periods?

RESPONSE:

- We thank the reviewer for these helpful suggestions. We have repeated production studies with an extended 14-day period and indeed observed a notable increase in POP-FA titers: We revised Figure 3E as well as the corresponding main text to report the following in Line 341:

  "We thus extended the cultivation time from previously reported 4 days to 7 days, and observed 1.9- to 4.4-fold increased and more consistent product titers. Further extending cultivation time to 14 days reduced consistency, but presented an additional increase in titers by 1.8- to 4.8-fold, to a total increase of 4- to 18-fold as compared to 4 day cultivations (**Figure 3E**). We determined that together with optimized POP-FA extraction methods,[18] a 7-day cultivation time enabled a balance of consistent, increased titers and rapid Design-Build-Test cycles to explore other *pop* gene clusters in *S. coelicolor*, detect a wide range of POP-FA products with diverse chain length, and systematically compare product profiles despite a potentially limited titer for minor POP-FA products. Given the increased yet inconsistent titers, we reserved scaled-up 14 day cultivations for compound isolation and more detailed analysis of products from selected pathways."

For Table 1, the authors assign the structures of the POP-FA products based on LC-MS analysis. However, additional details are needed, particularly regarding how the positions of the double bonds were determined. Further data, such as NMR, would be necessary to confirm the structure of the novel class III POP-FAs.

RESPONSE:

- We thank the reviewer for this suggestion. Given both the low titers and mixed nature of POP pathway products, we found through multiple rounds of preparative HPLC method optimization that separation of unique POP-FA species from each other (e.g., separating a C14:CP5 from a C14:CP6 POP-FA) for NMR characterization was particularly challenging. However, leveraging the increased production titers of a 14-day fermentation as well as an increased culture volume, we have extracted, partially purified and analyzed via MS/MS (now in updated **Figures S15-18**) the structure of the novel class III POP-FAs from the PLPP pathway (strain KY16). We have additionally revised the language of the manuscript to clarify that class II POP-FA structures remain hypothetical and require further experimental validation (See Response to Reviewer 1, comment #2).

In Figures 4b and 5c, the chosen color maps make it difficult to distinguish between the different cyclopropanation levels and chain lengths. An alternative color map should be considered for greater contrast.

RESPONSE:

- We thank the reviewer for this feedback. While the color maps for Figures 4b and 5c were originally chosen with the intent to focus on only chain length or only cyclopropanation levels, respectively, we agree that it lacks contrast between products of the same chain length or the same cyclopropanation level. We have thus recolored the borders of the bar graphs in Figures 5b, 5c, and S25 to indicate chain length alongside the cyclopropanation color scheme. However, after finding that a similar approach appeared too distracting and cluttered for the inverse color scheme in Figures 4a and 4b, we maintained the original color map for Figure 4. Nevertheless, several key product profiles from Figures 4a and 4b, e.g., POP3.4 and KY12 data, are covered again in Figure 5. Furthermore, a more granular analysis of the relative signal of individual POP-FA species remains possible with the supplemental chromatograms (now **Figures S26-27** and **S29-S37**).

Figure 4d, 5e, Given the nature of the compounds, further details are essential, particularly concerning the determination of the positions of the double bonds. If these are indeed new compounds in literature, NMR analysis would be preferable.

RESPONSE:

- We thank the reviewer for this comment and agree that further details concerning the position of double bonds are essential. We have similarly responded to Reviewer 1's 2nd comment that after rounds of LC-MS/MS method optimization and significant production titer increase and culture scale up, we observed fragment ions with m/z values that partially validate our previously uncharacterized, fully cyclopropanated class III POP-FAs from strain PLPP (**Figures S15-18**). Additionally, as we optimized our LC methods, we observed increasingly split chromatogram peaks that may correspond to class I and class II structural isomers resulting from different double bond positions; it is noteworthy that the EICs of POP-FAs that are saturated with CP rings and thus lack double bonds or any possibility for structural isomers, retained clean singular peaks as illustrated in Figure_Response 2.

The paragraph lines 489-505: The results from the chimeric POP-FA strains provide valuable insights into the mechanistic basis of the distinct product distributions. Based on the mechanism proposed by Oikawa et al. (2017), it can be inferred that the balance between iPKS substrate specificities and the rate of cyclopropanation is critical for controlling chain length and the cyclopropane ratio, as illustrated by conclusions from Figures 4, 5, and S14. However, the current description in the manuscript is challenging to follow for audience who have not read the previous study. A schematic illustration would enhance understanding, along with a concise discussion of the necessary kinetic properties of the various PKS components required to achieve the target molecular structure.

RESPONSE:

We thank the reviewer for this suggestion. To enhance understanding, we have added a schematic illustration, Scheme 1:

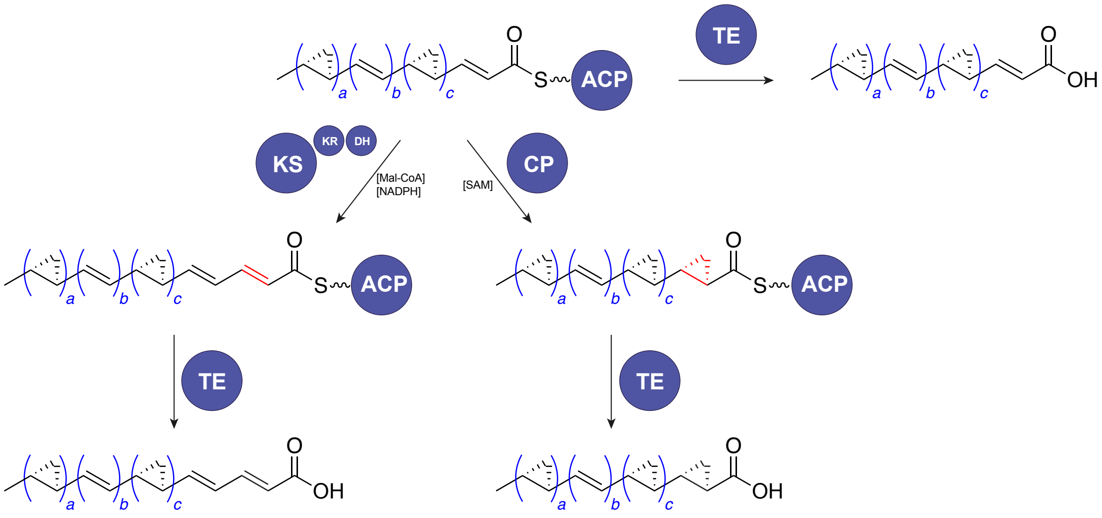

Scheme 1. Proposed competition-driven product programming mechanism. During each extension cycle, three reactions compete for the ACP-bound intermediate. Depending on the specificity and kinetics of competing enzymes, as well as on substrate flux and likely other factors, each intermediate may undergo either a cyclopropanation-skipping extension (in which the KS outcompetes the CP and TE), a cyclopropanation (in which the CP outcompetes the KS and TE), or chain release (in which the TE outcompetes the KS and CP).

Because during our revision we observed an unexpected increase in the relative abundance of C20:CP9 when performing 14-day cultivations with strain KY16 (PLPP), and because we did not observe a similar change to product profile between 7- and 14-day cultivations with strain POP3.4 (PPPP), we included substrate flux in our discussion of the proposed product control mechanism, beginning in Line 576:

"Intriguingly however, when increasing cultivation time to 14 days, the product profile of KY16 (PLPP) shifted with a notable increase in the longer C20:CP9 product (**Figure S26)**. Here, late-stage mobilization of cellular triacylglycerol (TAG)[4] increases carbon flux towards polyketide biosynthesis; this may improve the kinetics of the Pfl1 KS reaction to outcompete the TE and perform additional extensions. Finally, the specificity of Pfl1 KS may strictly disfavor substrates longer than C20-length intermediates, as a similar increase in cultivation time to 14 days did not significantly shift the product profile of the native fuelimycin (POP3.4, PPPP) pathway (**Figure S27**). If purification of these enzymatic subunits is achieved and an *in vitro* POP-FA production platform is established, this complex interplay of competing enzyme kinetics and substrate flux can be more deeply explored."

Lastly, we added a concise discussion of necessary kinetic changes to achieve our target molecules, beginning in Line 586:

"Additionally, as more efficient protein engineering methods[19,20] become applicable to megasynthases, the redesign of KS and CP specificities toward shorter substrates may provide the framework for stricter programming of POP-iPKS products."

Minor suggestions:
Line 136 "we predict these molecules to be precursors for fuels with energy density improvements and comparable freezing points to cyclopropanated myrcene."
Does this statement refer to the decarboxylated hydrocarbon derivatives? As presented in the introduction, this could be interpreted as referring to the FAME derivative.

RESPONSE:

- We thank the reviewer for this suggestion and have made clarifying edits accordingly, now beginning in Line 146:
  "we predict decarboxylated hydrocarbon derivatives of these molecules to be fuels with energy density improvements and comparable freezing points to cyclopropanated myrcene."

Line 153 "...predicted energy density is 45.14 MJ/L, despite having ten fewer main-chain carbons and two fewer cyclopropane rings."
This statement is misleading, as it seems to imply similar energy content per mole, whereas the numbers are referring to energy content per unit volume.

RESPONSE:

- We have revised the text to clarify as follows:

  "Based on our previous computational model, the predicted energy density as volumetric net heat of combustion (NHOC) of fuelimycin A-derived fatty acyl methyl ester (FAME), which contains three double bonds (C22:CP7-FAME), is 40.6 MJ/L. In comparison, C12:CP5-FAME's predicted volumetric NHOC is 45.14 MJ/L, despite having ten fewer main-chain carbons and two fewer cyclopropane rings."

The paragraph lines 199-209, again this seems to confuse energy content per mole vs. per unit volume. We may expect chain length of fully cyclopropanated hydrocarbon to affect melting point, with minor effect on density and energy content per unit mass or volume.

RESPONSE:

- We have similarly revised this section to clarify energy content per unit mass vs. per unit volume. We agree with the reviewer that chain length minorly affects energy content (whether per unit mass or volume), and emphasize that by contrast, chain length can more significantly reduce energy and substrate

consumption associated with bioproduction. It is precisely this factor, alongside the reduced melting point of shorter products, that motivates our study of chain length control.

Line 409 – is Figure S11 the correct reference?

RESPONSE:

Yes this is the correct reference; Figure S11 provides the proteomics data that confirms expression of the chimeric PKS pathways described. The text has been edited to clarify this (Now beginning in Line 451):

"To test this hypothesis, from our earlier phylogenetic analysis, we selected three CP genes from *S. ferralitis* (*ferCP*), *S. klenkii* (*kleCP*), and *S. longispororuber* (*log2*) (**Figure 5A**) based on evolutionary distance, substituted them into POP3.4, and confirmed their expression with proteomics analysis (**Figure S11**)."

Reviewer #5 (Remarks to the Author):

I co-reviewed this manuscript with one of the reviewers who provided the listed reports. This is part of the Nature Communications initiative to facilitate training in peer review and to provide appropriate recognition for Early Career Researchers who co-review manuscripts.

References

1. Barrett, A. G. M., Kasdorf, K., Tustin, G. J. & Williams, D. J. Determination of the full structure and absolute stereochemistry of the antifungal agent FR-900848: an X-ray crystallographic study of (1 R ,3 S ,4 R ,6 S ,7 S ,9 R ,10 S ,12 R)-quatercyclopropyl-1,12-dimethanediyl di-4-bromobenzoate. *Journal of the Chemical Society, Chemical Communications* **0**, 1143–1144 (1995).
2. Yoshida, M. *et al.* A novel antifungal antibiotic, FR-900848. I. Production, isolation, physico-chemical and biological properties. *J. Antibiot.* **43**, 748–754 (1990).
3. Hiratsuka, T., Suzuki, H., Minami, A. & Oikawa, H. Stepwise cyclopropanation on the polycyclopropanated polyketide formation in jawsamycin biosynthesis. *Org. Biomol. Chem.* **15**, 1076–1079 (2017).
4. Wang, W. *et al.* Harnessing the intracellular triacylglycerols for titer improvement of polyketides in Streptomyces. *Nat. Biotechnol.* **38**, 76–83 (2019).
5. Cruz-Morales, P. *et al.* Biosynthesis of polycyclopropanated high energy biofuels. *Joule* **6**, 1590–1605 (2022).
6. Tan, G.-Y. *et al.* Heterologous biosynthesis of spinosad: An omics-guided large polyketide synthase gene cluster reconstitution in Streptomyces. *ACS Synth. Biol.* **6**, 995–1005 (2017).
7. Du, G. *et al.* Influence of cluster-situated regulator PteF in filipin biosynthetic cluster on avermectin biosynthesis in Streptomyces avermitilis. *Biology (Basel)* **13**, 344 (2024).

8. Zhang, J. *et al.* A microbial supply chain for production of the anti-cancer drug vinblastine. *Nature* **609**, 341–347 (2022).
9. Wang, X. *et al.* Heterologous production of chlortetracycline in an industrial grade Streptomyces rimosus host. *Appl. Microbiol. Biotechnol.* **103**, 6645–6655 (2019).
10. Banerjee, D. *et al.* Genome-scale and pathway engineering for the sustainable aviation fuel precursor isoprenol production in Pseudomonas putida. *Metab. Eng.* **82**, 157–170 (2024).
11. Kamruzzaman, M., Landera, A., Menon, N. C., Allendorf, M. D. & Poorey, K. P2MAT: A machine learning (ML) driven software for Property Prediction of MATerial. (2024) doi:10.26434/chemrxiv-2024-0jfmb.
12. Hou, X.-J. & Huang, M.-B. Internal rotations of vinylcyclopropane, cyclopropanecarboxaldehyde, and cyclopropanecarboxylic acid: a DFT B3LYP study. *Theochem* **585**, 93–104 (2002).
13. Bernardi, F., Bottoni, A. & Miscione, G. P. DFT study of the palladium-catalyzed cyclopropanation reaction. *Organometallics* **20**, 2751–2758 (2001).
14. Bachrach, S. M. DFT study of [2.2]-, [3.3]-, and [4.4]paracyclophanes: strain energy, conformations, and rotational barriers. *J. Phys. Chem. A* **115**, 2396–2401 (2011).
15. Novak, I. Ring strain in [n]ladderanes. *J. Phys. Chem. A* **112**, 10059–10063 (2008).
16. El-Azhary, A. A. & Suter, H. U. Comparison between optimized geometries and vibrational frequencies calculated by the DFT methods. *J. Phys. Chem.* **100**, 15056–15063 (1996).
17. Wang, M. *et al.* Molecular geometries and vibrational contributions to reaction thermochemistry are surprisingly insensitive to the choice of basis sets. *J. Chem. Theory Comput.* **19**, 5036–5046 (2023).
18. Yin, K., Cruz-Morales, P., Whitford, C. M. & Keasling, J. D. Heterologous production of polycyclopropanated fatty acids and their methyl esters in Streptomyces. *STAR Protoc* **4**, 102190 (2023).
19. Jiang, K. *et al.* Rapid in silico directed evolution by a protein language model with EVOLVEpro. *Science* **387**, eadr6006 (2025).
20. Singh, N. *et al.* A generalized platform for artificial intelligence-powered autonomous enzyme engineering. *Nat. Commun.* **16**, 5648 (2025).

Reviewer #1 (Remarks to the Author):

Appropriate corrections have been made to comments 1-3. In particular, comment 2 has been suitably corrected by improving the yield through further experiments, collecting additional data, and clearly stating that the position of the double bond could not be identified. I have therefore recommended that this paper be accepted.

Response: We thank the reviewer for their helpful suggestions, comments, and critical evaluation throughout the revision process.

Reviewer #2 (Remarks to the Author):

The authors have responded very well to my comments regarding scaling/titers, and I have no further issues. It is very nice work

Response: We thank the reviewer for their helpful suggestions, in particular with regards to the longer fermentation times to improve production titers.

Reviewer #3 (Remarks to the Author):

My concerns were addressed. I am now ok with publishing this manuscript.

Response: We thank the reviewer for their helpful suggestions, comments, and critical evaluation throughout the revision process.

Reviewer #4 (Remarks to the Author):

The addition of LC-MS/MS data substantially strengthens the structural assignments, particularly for the fully cyclopropane-saturated class III products, which represent a central focus of the study. Although full NMR characterization was not feasible, the authors have appropriately moderated their structural claims and clearly acknowledged the limitations regarding double bond positioning. The main conclusions concerning programmable control of chain length and cyclopropane saturation are well supported by the revised data, and the previous concerns have been adequately addressed.

Response: We thank the reviewer for their critical evaluation of our structural claims and of our LC-MS/MS results.

Reviewer #5 (Remarks to the Author):

I co-reviewed this manuscript with one of the reviewers who provided the listed reports. This is part of the Nature Communications initiative to facilitate training in peer review and to provide appropriate recognition for Early Career Researchers who co-review manuscripts.

Response: We thank the reviewer for their helpful suggestions, comments, and critical evaluation throughout the revision process.
